# Ethylene-responsive CmERF3−CmMYB4 cascade suppresses anthocyanin biosynthesis in *Chrysanthemum* ×*morifolium*

**DOI:** 10.1093/hr/uhag132

**Published:** 2026-04-07

**Authors:** MengLing Li, WenJing Zhao, ShuangDa Li, XiaoHui Wen, Xiang Song, BoHao Wang, YuanYuan Wei, BoXiao Fu, LuYao Wang, YuanQi Dou, PengRui Jiang, He Huang, SiLan Dai, Yan Hong

**Affiliations:** Beijing Key Laboratory of Ornamental Plants Germplasm Innovation and Molecular Breeding, National Engineering Research Center for Floriculture, Beijing Laboratory of Urban and Rural Ecological Environment, Key Laboratory for Genetics and Breeding of Forest Trees and Ornamental Plants of Ministry of Education, School of Landscape Architecture, Beijing Forestry University, Beijing 100083, China; South China Botanical Garden, Chinese Academy of Sciences, Guangzhou 510650, China; Beijing Key Laboratory of Ornamental Plants Germplasm Innovation and Molecular Breeding, National Engineering Research Center for Floriculture, Beijing Laboratory of Urban and Rural Ecological Environment, Key Laboratory for Genetics and Breeding of Forest Trees and Ornamental Plants of Ministry of Education, School of Landscape Architecture, Beijing Forestry University, Beijing 100083, China; Beijing Key Laboratory of Ornamental Plants Germplasm Innovation and Molecular Breeding, National Engineering Research Center for Floriculture, Beijing Laboratory of Urban and Rural Ecological Environment, Key Laboratory for Genetics and Breeding of Forest Trees and Ornamental Plants of Ministry of Education, School of Landscape Architecture, Beijing Forestry University, Beijing 100083, China; Zhejiang Institute of Landscape Plants and Flowers, Zhejiang Academy of Agricultural Sciences, Hangzhou 311251, China; Beijing Key Laboratory of Ornamental Plants Germplasm Innovation and Molecular Breeding, National Engineering Research Center for Floriculture, Beijing Laboratory of Urban and Rural Ecological Environment, Key Laboratory for Genetics and Breeding of Forest Trees and Ornamental Plants of Ministry of Education, School of Landscape Architecture, Beijing Forestry University, Beijing 100083, China; Beijing Key Laboratory of Ornamental Plants Germplasm Innovation and Molecular Breeding, National Engineering Research Center for Floriculture, Beijing Laboratory of Urban and Rural Ecological Environment, Key Laboratory for Genetics and Breeding of Forest Trees and Ornamental Plants of Ministry of Education, School of Landscape Architecture, Beijing Forestry University, Beijing 100083, China; Beijing Key Laboratory of Ornamental Plants Germplasm Innovation and Molecular Breeding, National Engineering Research Center for Floriculture, Beijing Laboratory of Urban and Rural Ecological Environment, Key Laboratory for Genetics and Breeding of Forest Trees and Ornamental Plants of Ministry of Education, School of Landscape Architecture, Beijing Forestry University, Beijing 100083, China; Beijing Key Laboratory of Ornamental Plants Germplasm Innovation and Molecular Breeding, National Engineering Research Center for Floriculture, Beijing Laboratory of Urban and Rural Ecological Environment, Key Laboratory for Genetics and Breeding of Forest Trees and Ornamental Plants of Ministry of Education, School of Landscape Architecture, Beijing Forestry University, Beijing 100083, China; Beijing Key Laboratory of Ornamental Plants Germplasm Innovation and Molecular Breeding, National Engineering Research Center for Floriculture, Beijing Laboratory of Urban and Rural Ecological Environment, Key Laboratory for Genetics and Breeding of Forest Trees and Ornamental Plants of Ministry of Education, School of Landscape Architecture, Beijing Forestry University, Beijing 100083, China; Beijing Key Laboratory of Ornamental Plants Germplasm Innovation and Molecular Breeding, National Engineering Research Center for Floriculture, Beijing Laboratory of Urban and Rural Ecological Environment, Key Laboratory for Genetics and Breeding of Forest Trees and Ornamental Plants of Ministry of Education, School of Landscape Architecture, Beijing Forestry University, Beijing 100083, China; Beijing Key Laboratory of Ornamental Plants Germplasm Innovation and Molecular Breeding, National Engineering Research Center for Floriculture, Beijing Laboratory of Urban and Rural Ecological Environment, Key Laboratory for Genetics and Breeding of Forest Trees and Ornamental Plants of Ministry of Education, School of Landscape Architecture, Beijing Forestry University, Beijing 100083, China; Beijing Key Laboratory of Ornamental Plants Germplasm Innovation and Molecular Breeding, National Engineering Research Center for Floriculture, Beijing Laboratory of Urban and Rural Ecological Environment, Key Laboratory for Genetics and Breeding of Forest Trees and Ornamental Plants of Ministry of Education, School of Landscape Architecture, Beijing Forestry University, Beijing 100083, China; Beijing Key Laboratory of Ornamental Plants Germplasm Innovation and Molecular Breeding, National Engineering Research Center for Floriculture, Beijing Laboratory of Urban and Rural Ecological Environment, Key Laboratory for Genetics and Breeding of Forest Trees and Ornamental Plants of Ministry of Education, School of Landscape Architecture, Beijing Forestry University, Beijing 100083, China; Beijing Key Laboratory of Ornamental Plants Germplasm Innovation and Molecular Breeding, National Engineering Research Center for Floriculture, Beijing Laboratory of Urban and Rural Ecological Environment, Key Laboratory for Genetics and Breeding of Forest Trees and Ornamental Plants of Ministry of Education, School of Landscape Architecture, Beijing Forestry University, Beijing 100083, China

## Abstract

Anthocyanins are the primary determinants of floral pigmentation in *Chrysanthemum* ×*morifolium*, and ethylene acts as a key regulator of their biosynthesis. Although the ethylene-mediated regulatory circuitry is functionally important, its underlying mechanism in chrysanthemum has remained unclear. In this study, we identified CmMYB4 as a transcriptional repressor that directly suppresses the expression of key anthocyanin biosynthetic genes, including *CmDFR* (dihydroflavonol 4-reductase), *CmUFGT* (flavonoid 3-*O*-glucosyltransferase), and *Cm3MaT* (anthocyanin 3-*O*-glucoside-6″-*O*-malonyltransferase). Time-ordered gene co-expression network analysis comparing the transcriptomes of *CmMYB4*-overexpressing and control plants, together with molecular biology experimental results, further revealed *CmERF3* (*ethylene response factor 3*) as a hierarchical upstream regulator of anthocyanin biosynthesis. Exogenous ethylene treatment induced *CmERF3* expression while reducing anthocyanin accumulation. Subsequent functional characterization showed that the overexpression of *CmERF3* suppresses anthocyanin biosynthesis in both tobacco and chrysanthemum by directly activating *CmMYB4* and repressing *CmDFR*, *CmUFGT*, and *Cm3MaT*. Collectively, these findings revealed that ethylene inhibits anthocyanin biosynthesis through a CmERF3−CmMYB4−LBGs (late biosynthetic genes) regulatory module. This study not only elucidates the molecular mechanism governing ethylene-mediated anthocyanin inhibition but also provides new perspectives for the molecular engineering of ornamental traits in chrysanthemum.

## Introduction

Anthocyanins, a class of water-soluble flavonoid compounds, play crucial roles in mediating pollination, facilitating seed dispersal, and enhancing stress protection mechanisms [1–4]. The accumulation patterns of these pigments in floral and fruit directly determine coloration characteristics, thereby impacting their horticultural appeal and agricultural marketability. Although studies have demonstrated that anthocyanin biosynthesis is coordinately regulated by biotic and abiotic factors, the molecular orchestration underlying this process remains incompletely understood [5–7].

The anthocyanin biosynthetic pathway is catalyzed by a conserved enzymatic cascade comprising chalcone synthase (CHS), chalcone isomerase (CHI), flavanone-3-hydroxylase (F3H), flavonoid 3′-hydroxylase (F3′H)/flavonoid 3′5′-hydroxylase (F3′5′H), dihydroflavonol 4-reductase (DFR), anthocyanidin synthase (ANS), UDP-glucose: flavonoid 3-*O*-glucosyltransferase (UFGT), and anthocyanin 3-*O*-glucoside-6″-*O*-malonyltransferase (3MaT). Phylogenetic analysis classifies *CHS* and *CHI* as early biosynthetic genes (EBGs), which regulate the commitment steps in flavonoid backbone formation. In contrast, *DFR*, *ANS*, *UFGT*, and *3MaT* are categorized as late biosynthetic genes (LBGs) that directly catalyze anthocyanin biosynthesis. Among these, UFGT and 3MaT play particularly important roles by mediating key glycosylation and malonylation modifications, thereby enhancing the stability and structural diversity of anthocyanins [8, 9]. The temporal regulation of these biosynthetic genes is orchestrated by the evolutionarily conserved MYB (v-myb avian myeloblastosis)−bHLH (basic helix–loop–helix)−WD40 (WD40 repeat domain) (MBW) ternary complex, wherein R2R3-MYB transcription factors (TFs) serve as the regulatory determinants through sequence-specific recognition of *cis*-elements in target promoters [10–12].

R2R3-MYB TFs have been extensively characterized across diverse plant species, spanning model plants such as Arabidopsis (*Arabidopsis thaliana*) to horticulturally important crops including petunia (*Petunia* spp.), apple (*Malus domestica*), and pear (*Pyrus* spp.) [10, 13–21]. Phylogenetic analyses categorize these regulators into evolutionarily conserved subgroups (SGs) exhibiting functional conservation within clades [10, 22, 23]. Notably, SG6 members of the R2R3-MYBs (e.g. AtMYB75/PAP1, PhAN2, MdMYB10, and AmROSEA1) harboring the (R/K)PRPRx(F/L) signature motif function as activators of anthocyanin biosynthesis [16, 24–26]. In contrast, SG4 members (e.g. FaMYB1, AtMYB4, PhMYB27, and PtMYB182) encode transcriptional repressors [13, 27–29]. SG4 R2R3-MYBs can be further classified into a general phenylpropanoid and lignin group and a flavonoid group. Intriguingly, both repression subtypes retain intact bHLH-interaction domains, yet functional divergence emerges in their partner selectivity [6, 30, 31]. Empirical evidence demonstrates that only flavonoid-targeting repressors (e.g. anthocyanin or proanthocyanidin regulators) engage in physical interactions with bHLH proteins [31]. Structurally, these MYB repressors feature C-terminal regulatory modules: the C1/GIDP motif (characteristic of SGs 8, 9, and 11) confers transcriptional activation potential, whereas the C2/EAR motif constitutes the primary repression domain [32]. Additionally, a subset harbors the TLLLFR repression motif, suggesting combinatorial control of inhibitory functions [29, 33]. Mechanistically, SG4 R2R3-MYBs exert bimodal repression through: (i) competitive displacement of SG6 activators from bHLH-binding interfaces in the MBW complex and (ii) direct transcriptional suppression via promoter occupancy of structural genes [6, 30, 31]. This dual-layered regulatory paradigm not only fine-tunes anthocyanin biosynthesis but also provides evolutionary plasticity for metabolic channeling. However, the SG4 R2R3-MYB that regulates anthocyanin synthesis has not yet been identified in chrysanthemums, and its mechanism of action remains to be elucidated.

Anthocyanin accumulation is dynamically modulated by an array of environmental and developmental factors, spanning temperature fluctuations, spectral light composition, nutrient availability, and phytohormonal signaling [5, 6]. Among these regulators, ethylene (C₂H₄), a gaseous phytohormone, orchestrates diverse developmental processes including seed germination, secondary metabolism, fruit ripening, senescence progression, organ abscission, and abiotic stress responses [34–36]. Notably, ethylene exerts species-specific modulation of anthocyanin biosynthesis, manifesting as divergent regulatory effects across plant taxa: suppressive action in Arabidopsis seedlings, pear fruits, and lily (*Lilium* spp.) petals, contrasted by activating effects in apple and plum (*Prunus* spp.) [37–41].

The ethylene signal transduction pathway has been thoroughly revealed, centering on ethylene response factor (ERF) TFs within the APETALA2 (AP2)/ERF family [42–44]. Structurally, ERFs harbor AP2 DNA-binding domains that confer sequence-specific recognition of GCC box (GCCGCC) or dehydration-responsive elements (CCGAC) [45, 46]. Emerging evidence positions ERFs as multifunctional regulators of anthocyanin biosynthesis, exhibiting species- and context-dependent operational modes. In horticultural species, including apple, pear, citrus (*Citrus sinensis*), litchi (*Litchi chinensis*), grape (*Vitis vinifera*), and lily, ERFs predominantly regulate anthocyanin accumulation via *MYB* promoter binding, either potentiating or suppressing transcriptional activation ([38, 41, 47–51]). Intriguingly, protein–protein interaction networks between ERFs and MYBs, such as in citrus, pear, and apple, enhance DNA-binding cooperativity at structural gene promoters, thereby amplifying anthocyanin production ([21, 49, 50, 52]). A recent breakthrough in pear revealed competitive interference: PyERF4.1/4.2 and PyERF3 destabilize the PyERF3−PyMYB114−PybHLH3 transcriptional activator complex, impairing anthocyanin pigmentation [53]. Despite these advances, taxon-specific divergence in ethylene–anthocyanin crosstalk persists, particularly regarding: (i) evolutionary drivers underlying species-dependent ERF functionalization; (ii) spatiotemporal dynamics of ERF-MYB regulatory circuits in non-model species; and (iii) epigenetic modulation of ethylene-responsive pigment patterning. Therefore, it is extremely important to identify the key *ERF* genes in a species and characterize their functions.

Chrysanthemum (*Chrysanthemum*×*morifolium*), a globally significant ornamental species renowned for its diverse pigmentation patterns, holds substantial commercial value in the floriculture industry. Previous transcriptome analysis identified CmMYB4 as a negative regulator of anthocyanin biosynthesis, as evidenced by heterologous expression intobacco (*Nicotiana tabacum*) [54, 55]. Nevertheless, the mechanistic basis of CmMYB4-mediated regulation and its upstream signaling components remain poorly characterized. In this study, the inhibitory function of *CmMYB4* on anthocyanin biosynthesis was verified in transgenic chrysanthemum. Further analysis showed that CmMYB4 binds to the promoters of *CmDFR*, *CmUFGT*, and *Cm3MaT* and inhibits their transcription. Then, transcriptomic comparison between *CmMYB4*-overexpressing and control plants in the time-ordered gene co-expression networks (TO-GCNs), together with molecular biology experimental results, revealed CmERF3 as a critical upstream regulator. Subsequent investigations elucidated that the expression of *CmERF3* was induced by ethephon treatment. Finally, the function and molecular mechanism of CmERF3 in negatively regulating anthocyanin biosynthesis in chrysanthemum were explored. Collectively, our data reveal the mechanism of CmERF3−CmMYB4−LBGs regulatory module in ethylene-inhibited anthocyanin biosynthesis in chrysanthemum, which provides new insight into the molecular engineering of ornamental traits in chrysanthemum.

## Results

### Overexpression of *CmMYB4* in chrysanthemum inhibits anthocyanin production

**Figure 1:**
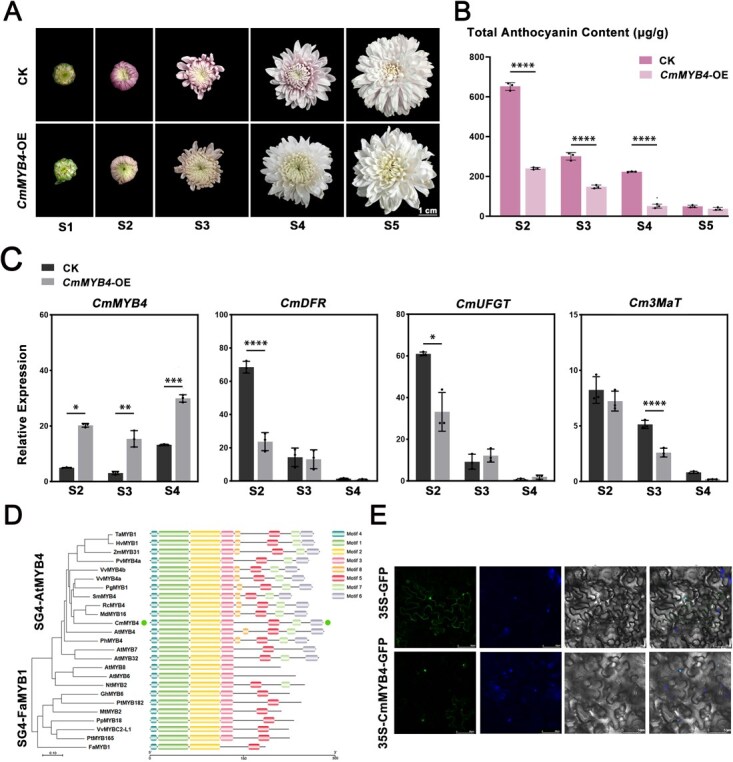
Functional characterization of *CmMYB4* in anthocyanin biosynthesis. (A) Phenotypic characteristics of chrysanthemum capitula at different developmental stages in the control (CK) and *CmMYB4*-overexpressing (OE) lines. Scale bar = 1 cm. (B) Total anthocyanin content in ray florets during S2−S5 stages in CK and *CmMYB4*-OE lines. (C) Expression patterns of anthocyanin biosynthetic genes in CK and *CmMYB4*-OE lines. (D) Phylogenetic analysis of the R2R3-MYB subgroup (SG) 4 members. (E) Subcellular localization of CmMYB4 in tobacco (*N. benthamiana*) leaf epidermal cells. Asterisks indicate statistically significant differences (^*^p <0 .05, ^**^p <0 .01, ^***^p <0 .001, ^****^p<0 .0001). DFR, dihydroflavonol 4-reductase; UFGT, UDP-glucose: flavonoid 3-*O*-glucosyltransferase; 3MaT, anthocyanin 3-*O*-glucoside-6″-*O*-malonyltransferase; GFP, green fluorescent protein.

To functionally validate the regulatory role of CmMYB4 *in planta*, we generated constitutive overexpression lines of *CmMYB4* in *C.*×*morifolium* ‘Riqie Taohong’. Phenotypic analysis of several transformed lines revealed hypopigmentation in ray florets, particularly during the capitulum developmental stages S2−S4 (Fig. 1A). High-performance liquid chromatography (HPLC) quantification confirmed a significant reduction in the total anthocyanin content compared to the control group (CK), correlating with visual phenotypes (Fig. 1B). qRT-PCR analysis revealed that the overexpression of *CmMYB4* caused stage-specific suppression of anthocyanin biosynthetic genes. The structural genes *CmCHS1*, *CmF3H*, *CmF3*′*H*, *CmANS*, *CmDFR*, and *CmUFGT* were significantly downregulated at the S2 stage, whereas *Cm3MaT* expression was specifically suppressed at S3 (Figs 1C and S1). This coordinated suppression closely matched the observed depletion of anthocyanins, confirming that CmMYB4 acts as a repressor of anthocyanin biosynthesis in chrysanthemum. The stage-specific differences in gene suppression may be attributed to the inherent temporal expression patterns of these structural genes during flower development, as well as variations in their promoter sensitivities to repression by CmMYB4.

A phylogenetic tree was constructed, and conserved motif analysis was performed to elucidate the functional characteristics of the *CmMYB4*-encoded protein (Fig. 1D). Building upon the prior classification of CmMYB4 within the R2R3-MYB SG4, our phylogenetic analysis now definitively clusters it within the ‘AtMYB4-type’ clade. This clade includes well-characterized transcriptional repressors of the general phenylpropanoid pathway, such as AtMYB4, AtMYB32, VvMyb4a, and VvMyb4b. Conserved motif analysis identified a highly conserved R2R3 domain (motifs 1, 2, and 4) at the N-terminal region of CmMYB4, containing a characteristic bHLH-binding site that suggests potential interaction with bHLH cofactors. Furthermore, a C2 motif (pdLNLD/ELxiG/S, motif 5) was detected at the C-terminal region, corresponding to the ethylene response factor-associated amphiphilic repression (EAR) motif. Notably, previous studies have demonstrated that mutations or deletions in this EAR motif result in complete loss of repressive activity [56].

To determine the subcellular localization of CmMYB4, we transiently expressed a CmMYB4-GFP (green fluorescent protein) fusion protein and a GFP-only control in tobacco (*N. benthamiana*) leaf epidermal cells. Confocal microscopy revealed that the CmMYB4-GFP signal was enriched in the nucleus, a pattern distinct from the diffuse cytosolic and nuclear signal of the GFP control (Fig. 1E). This nuclear localization supports the role of CmMYB4 as a transcriptional regulator.

### CmMYB4-mediated transcriptional suppression of anthocyanin biosynthetic genes

**Figure 2:**
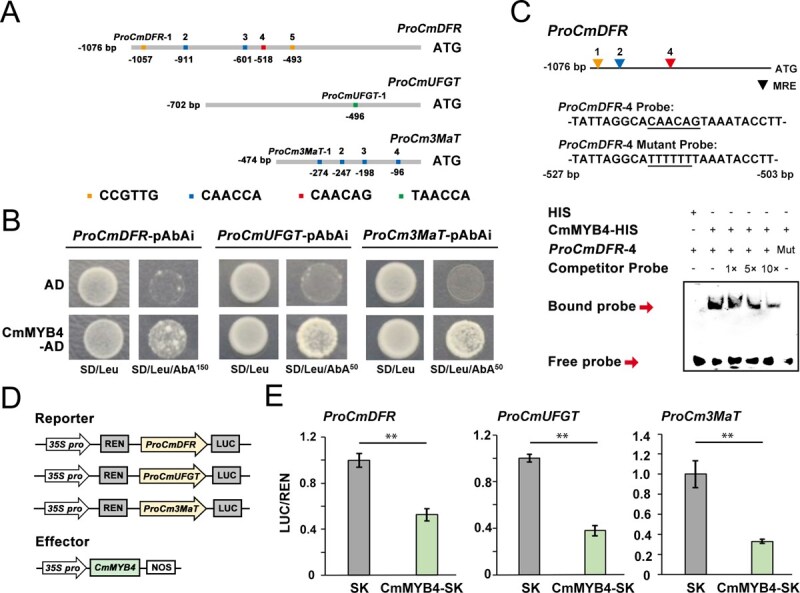
Inhibitory effect of CmMYB4 on anthocyanin biosynthetic structural genes. (A) Schematic representation of MREs within the promoters of *CmDFR*, *CmUFGT*, and *Cm3MaT*. (B) Yeast one-hybrid assays demonstrating CmMYB4 binding specificity to the *CmDFR*, *CmUFGT*, and *Cm3MaT* promoters. (C) Electrophoretic mobility shift assays confirmed that CmMYB4 binds to the CTGTTG motif in the *CmDFR* promoter *in vitro*. (D) Vector architecture for dual-luciferase reporter (DLR) assays. (E) DLR assays indicated that CmMYB4 represses the activities of the *CmDFR*, *CmUFGT*, and *Cm3MaT* promoters. Asterisks indicate statistically significant differences (^**^p < 0.01) DFR, dihydroflavonol 4-reductase; UFGT, UDP-glucose: flavonoid 3-*O*-glucosyltransferase; 3MaT, anthocyanin 3-*O*-glucoside-6″-*O*-malonyltransferase.

To investigate the direct transcriptional regulation of anthocyanin biosynthetic genes by CmMYB4, yeast one-hybrid (Y1H), electrophoretic mobility shift assay (EMSA), and dual-luciferase reporter (DLR) assays were performed. Bioinformatics analysis of promoter regions of *CmDFR*, *CmUFGT*, and *Cm3MaT* using PlantCARE identified multiple MYB recognition elements (MREs), with 5, 1, and 4 MREs, respectively (Fig. 2A). Y1H assays demonstrated direct binding of CmMYB4 to the promoter of *CmDFR*, *CmUFGT*, and *Cm3MaT*, as evidenced by robust growth of yeast strains Y1H[*CmDFRpro*-AbAi], Y1H[*CmUFGTpro*-AbAi], and Y1H[*Cm3MaTpro*-AbAi] transformed with AD-CmMYB4 on selective SD/-Leu media containing AbA (Fig. 2B).

To pinpoint the specific *cis*-element within the *CmDFR* promoter recognized by CmMYB4, we performed EMSA. The results definitively identified the CTGTTG motif as the binding site. The protein−DNA binding signal was competitively reduced by unlabeled wild-type probes in a dose-dependent manner and was eliminated when the CTGTTG motif was mutated (Fig. 2C).

For functional validation, a tobacco leaf-based dual-luciferase assay was performed using *35Spro::CmMYB4* as the effector and *CmDFRpro*/*CmUFGTpro*/*Cm3MaTpro::LUC* as reporters, with *35Spro::REN* as the internal control (Fig. 2D). Quantitative analysis revealed significant transcriptional repression, as indicated by reduced LUC/REN ratios reduced upon co-expression of *CmMYB4* with the promoters of these genes (Fig. 2E). Collectively, these complementary results indicate that CmMYB4 directly binds the promoters of key anthocyanin biosynthetic genes (*CmDFR*, *CmUFGT*, and *Cm3MaT*), thereby mediating their transcriptional repression.

### Time-ordered regulatory networks of anthocyanin biosynthesis in chrysanthemum

**Figure 3:**
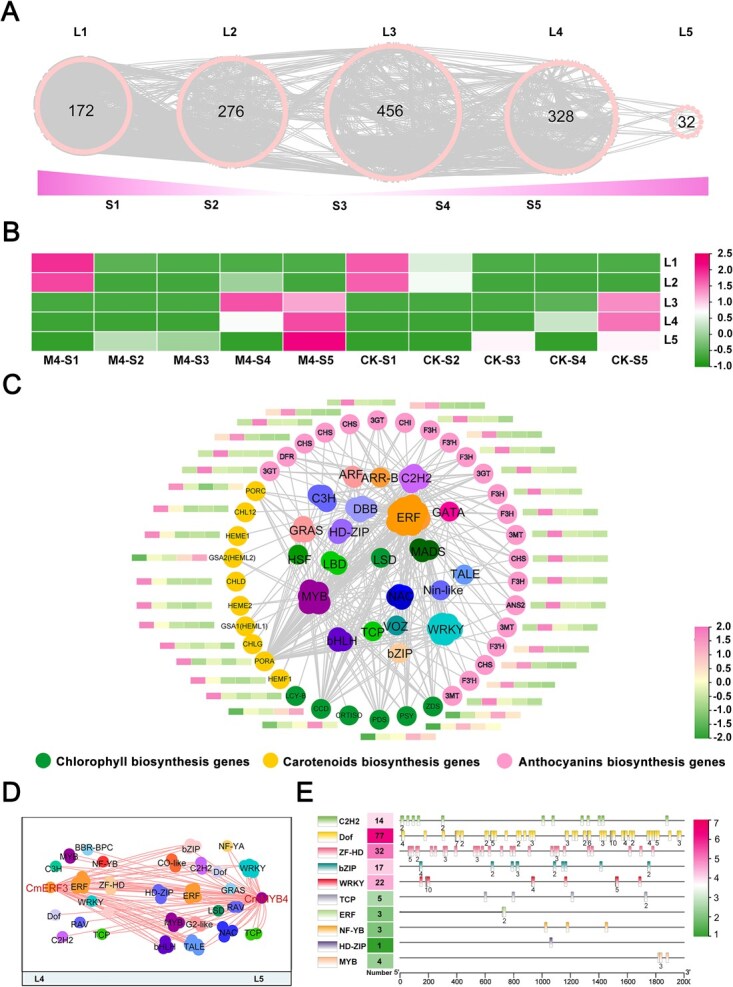
Hierarchical regulation network of floral pigmentation in *CmMYB4*-overexpressing chrysanthemum plants. (A) Architecture of time-ordered gene co-expression networks with 1260 TFs distributed across five temporal tiers (L1-L5). The circles are made up of discrete dots, with each dot corresponding to a transcription factor (TF). (B) Phasic expression dynamics mapped to the five capitulum developmental stages based on the *Z*-score normalized fragments per kilobase million values. (C) Metabolic subnetworks of genes related to the biosynthesis of chlorophylls (green circles), carotenoids (yellow circles), anthocyanins (pink circles), and the corresponding regulatory factors. (D) Multi-tiered regulation of *CmMYB4*. TFs in the same protein family are represented by circles of the same color, and the different levels to which they belong are marked. (E) *cis*-element prediction of *CmMYB4* promoter. The total number of binding sites for each TF is detailed on the left. M4, *CmMYB4*-overexpressing plants; CK, control plants.

To elucidate the hierarchical regulation of anthocyanin biosynthesis, we employed TO-GCN analysis using RNA-seq data from ray florets of *CmMYB4-overexpressing* and control plants across five capitulum developmental stages (S1-S5) (Fig. 3A; Table S3). The reconstructed TO-GCNs revealed: (i) global architecture with five temporally stratified modules (L1-L5) mirrored developmental progression; network topology analysis identified 1260 TFs distributed across these temporal layers; (ii) the phase-specific subnetworks correspond to the initial phase of anthocyanin accumulation (L1-L2), the transitional phase of pigment turnover (L3-L4), and the terminal phase of senescence-associated fading (L5) (Fig. 3A and B); and (iii) a core regulatory machinery comprising 139 key genes, including 101 regulatory genes and 38 enzyme genes, was identified and found to be correlated with pigment accumulation (Fig. 3C, Table S4). Notably, TFs in six families, such as ERF (*C.* ×*morifolium 15 148*), WRKY (*C.* ×*morifolium 1 749 144*), and MYB (*C.* ×*morifolium 175 740*), were predicted as direct regulators for anthocyanin biosynthesis. Gene Ontology enrichment revealed a significant association with the ethylene-activated signaling pathway, prompting subsequent analysis of expression patterns of ethylene biosynthesis and signaling pathway genes (Figs S2 and S3).

The hierarchical regulatory architecture of *CmMYB4* (*C.* ×*morifolium 175 740*) was reconstructed, and the upstream regulatory factors were screened (Fig. 3D). The regulatory network topology revealed *CmMYB4* as an enrichment center in the L5 module, computationally predicted to be co-regulated by multiple TFs at the L4 tier, including ERF, WRKY, NAC, C2H2, MYB, and bZIP.

Analysis of the *CmMYB4* promoter identified conserved *cis*-elements corresponding to predicted regulators (Fig. 3E), further confirming that CmMYB4 may be regulated by these TFs. Notably, preliminary Y1H screening using the *CmMYB4* promoter as bait identified *CmERF3* as a putative regulator (Table S5). This combinatorial evidence from transcriptional networks, *cis*-element profiling, and physical interaction assays suggests that *CmERF3* may function as a principal upstream regulator orchestrating *CmMYB4*-mediated anthocyanin repression.

### Exogenous ethylene induces the expression of *CmERF3* while inhibiting anthocyanin biosynthesis

**Figure 4:**
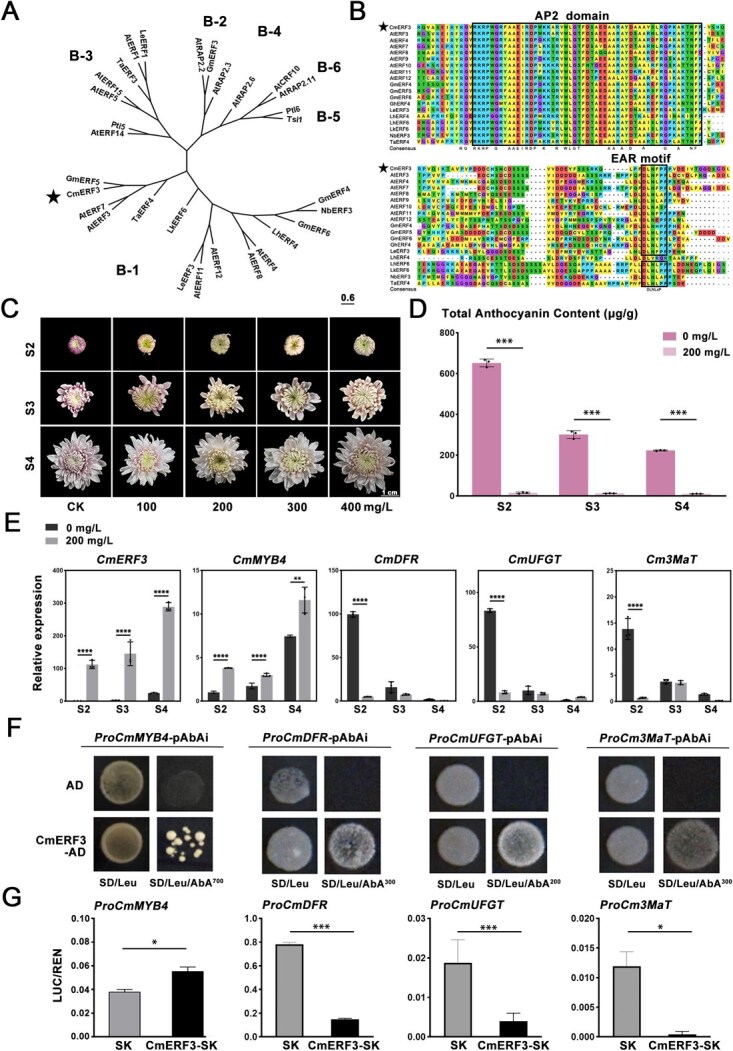
Exogenous ethylene induces the expression of *CmERF3* while inhibiting anthocyanin biosynthesis. (A) Phylogenetic tree of ERF proteins from Arabidopsis and other species. CmERF3 is marked with a black star. (B) Multiple sequence alignment of APETALA2 (AP2)/ERF DNA-binding domains. Conserved motifs (ethylene response factor-associated amphiphilic repression, EAR) are boxed. (C) Phenotypic effects of ethephon treatment across different capitulum developmental stages. (D) Total anthocyanin content in ray florets treated with 200 mg/L ethephon. (E) Temporal expression profiles of *CmERF3*, *CmMYB4*, and anthocyanin biosynthetic genes treated with 200 mg/L ethephon. (F) Yeast one-hybrid assays showed that CmERF3 can bind to the promoters of *CmMYB4*, *CmDFR*, *CmUFGT*, and *Cm3MaT*. (G) Dual-luciferase reporter assays showed that CmERF3 activates the expression of *CmMYB4* while inhibiting the expression of *CmDFR*, *CmUFGT*, and *Cm3MaT*. Asterisks indicate statistically significant differences (^*^p < 0.05, ^**^p < 0.01, ^***^p < 0.001) . CK, control; DFR, dihydroflavonol 4-reductase; UFGT, UDP-glucose: flavonoid 3-*O*-glucosyltransferase; 3MaT, anthocyanin 3-*O*-glucoside-6″-*O*-malonyltransferase.

Sequence analysis revealed that CmERF3 contains a 1069-bp genomic sequence, encompassing a 612-bp open reading frame that encodes a 204-aa polypeptide. Based on critical residue analysis showing alanine (A) at position 14 and aspartic acid (D) at position 19 within the AP2 domain, CmERF3 was classified as a member of the ERF subfamily within the AP2/ERF TF superfamily. Phylogenetic analysis classified CmERF3 within the ERF-B1 subgroup, demonstrating close phylogenetic relationships with AtERF3, 4, and 7, as well as GmERF5 in soybean (*Glycine max*) and LkERF6 in *Larix kaempferi* (Fig. 4A). These phylogenetic associations suggest potential functional conservation between CmERF3 and other B1 subgroup members. Structural characterization further revealed that the C-terminal region contains the evolutionarily conserved L/FDLNL/F(x)P sequence, characteristic of the EAR repressor motif (Fig. 4B). The temporal and spatial expression patterns of *CmERF3* were analyzed, and it was found that the transcription level of ERF gradually increased with flower development and reached the highest level in the S5 stage. In addition, the expression of ERF was also detected in leaves (Fig. S4). Subcellular localization analysis showed that CmERF3 is localized in the nucleus, supporting its function as a TF (Fig. S5).

To elucidate the regulatory role of ethylene in chrysanthemum pigmentation, capitula at developmental stage S1 (ray florets fully emerged from bracts and completely achromatic) were subjected to exogenous ethephon treatments (0, 100, 200, 300, 400 mg/L). Phenotypic analysis revealed a dose-dependent reduction in anthocyanin pigmentation following ethylene exposure (Fig. 4C). Exogenous application of 200 mg/L ethephon significantly reduced anthocyanin content in ray florets during S2-S4 (Fig. 4D). Consistent with this phenotype, qRT-PCR analysis showed strong induction of CmERF3 and CmMYB4, both of which were negatively correlated with anthocyanin accumulation, whereas the eight anthocyanin biosynthetic genes were markedly suppressed. This transcriptional response was most pronounced at the S2 stage (Figs 4E and S1). To assess the generality of this finding, we validated the effect of ethylene in the chrysanthemum cultivar ‘Aoshuang Fencai’. Consistent with our initial observations, ethylene treatment significantly attenuated ray floret pigmentation, which was associated with a substantial reduction in anthocyanin levels and the coordinated downregulation of key anthocyanin biosynthetic genes (Fig. S6).

To define the transcriptional hierarchy among CmERF3, CmMYB4, and the anthocyanin structural genes, we first investigated whether CmERF3 directly regulates *CmMYB4*. Y1H assays revealed that CmERF3 specifically binds to the *CmMYB4* promoter (Fig. 4F). This direct interaction was functionally confirmed by a DLR assay, in which CmERF3 significantly trans-activated the promoter of *CmMYB4* (Figs 4G and S7). Together, these results suggest that CmERF3 function as a direct transcriptional activator of *CmMYB4*. We extended our analysis to key structural genes and found that CmERF3 also directly binds and inhibits the promoters of *CmDFR*, *CmUFGT*, and *Cm3MaT* (Fig. 4F and G), indicating a dual regulatory role in the anthocyanin pathway.

### Overexpression of *CmERF3* suppresses anthocyanin biosynthesis in both tobacco and chrysanthemum

**Figure 5:**
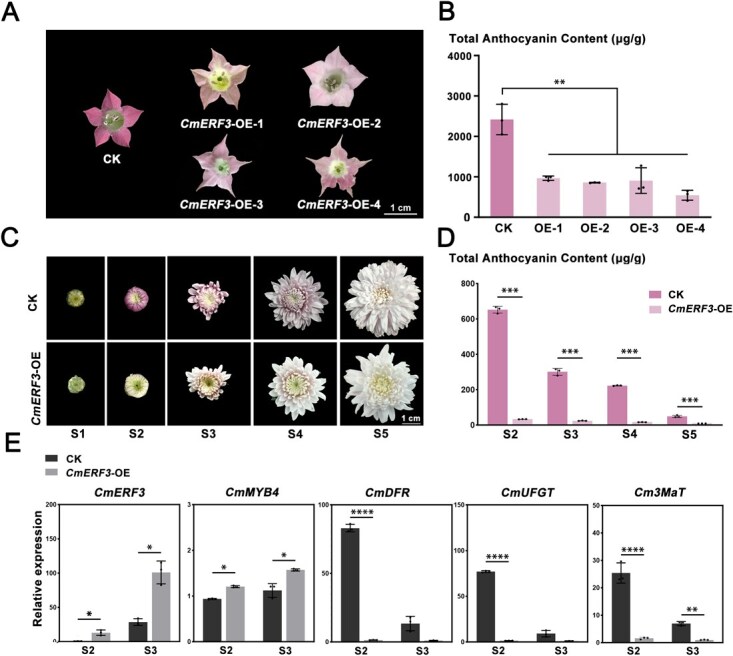
Overexpression of CmERF3 suppresses anthocyanin biosynthesis in both tobacco and chrysanthemum. (A) Overexpression of *CmERF3* in tobacco. (B) Total anthocyanin content in tobacco overexpressing *CmERF3*. (C) Overexpression of *CmERF3* in chrysanthemum. (D) Total anthocyanin content in chrysanthemum ray florets overexpressing *CmERF3.* Scale bar = 1 cm. (E) Expression patterns of genes related to anthocyanin biosynthesis in chrysanthemum ray florets overexpressing *CmERF3*. Asterisks indicate statistically significant differences (^*^p <0 .05, ^**^p <0 .01, ^***^p<0.001, ^****^p <0 .0001). ERF, ethylene response factor; DFR, dihydroflavonol 4-reductase; UFGT, UDP-glucose: flavonoid 3-*O*-glucosyltransferase; 3MaT, anthocyanin 3-*O*-glucoside-6″-*O*-malonyltransferase.

To functionally validate the repressive role of CmERF3 in anthocyanin biosynthesis, we generated *CmERF3*-overexpressing lines in both tobacco and chrysanthemum. Phenotypic assessment of *CmERF3*-overexpressing tobacco (T_1_ generation) and chrysanthemum transgenic lines consistently demonstrated significantly attenuated pigmentation in flowers compared to CK (Fig. 5A and B). Consistent with the observed hypopigmentation, the total anthocyanin content in transgenic lines was significantly reduced (Fig. 5C and D). This phenotype was associated with a substantial (~10-fold) upregulation of *CmERF3* transcripts at both S2 and S3 stages. Concomitant with this increase, we observed significant suppression of key anthocyanin biosynthetic genes that include *CmCHS1*, *CmCHS2*, *CmF3H*, *CmF3*′*H*, *CmDFR*, *CmANS*, and *CmUFGT* at the S2 stage. Furthermore, *Cm3MaT* expression was markedly downregulated at both S2 and S3 stages (Figs 5E and S1).

Our findings collectively demonstrate that ethylene signaling promotes the expression of *CmERF3*, which acts as a key repressor of anthocyanin biosynthesis. On one hand, CmERF3 activates the expression of the transcriptional repressor CmMYB4, thereby indirectly downregulating the genes *CmDFR*, *CmUFGT*, and *Cm3MaT*. Furthermore, the CmERF3 protein directly binds to the promoters of these structural genes to suppress their transcription. The combined effect of these direct and indirect mechanisms significantly reduces anthocyanin biosynthesis, explaining the lighter color observed in the ray florets.

## Discussion

### CmMYB4 acts as an active repressor of anthocyanin biosynthesis in chrysanthemum

Accumulating evidence indicates that R2R3-MYB transcriptional repressors negatively regulate anthocyanin biosynthesis in plants [27, 29, 33, 57]. These repressors, primarily clustered in SG4, typically contain conserved repressive motifs. Phylogenetic analysis classifies SG4 MYB members into two functional categories: (i) the ‘FaMYB1-type’ regulating flavonoid biosynthesis (including key steps of anthocyanin production) and (ii) the ‘AtMYB4-type’ modulating the general phenylpropanoid pathway responsible for phenolic acid and lignin synthesis [6, 30]. Notably, exceptions exist: for instance, apple MdMYB16 phylogenetically aligns with AtMYB4-type yet functionally represses anthocyanin biosynthesis [57]. In this study, CmMYB4 clustered with AtMYB4-type members (AtMYB4, AtMYB7, and AtMYB32) and harbored conserved C2 motif (pdLNLD/ELxiG/S).

The C2 motif, containing the characteristic EAR repression domain (LxLxL or DLNxxP), has been implicated in transcriptional repression of flavonoid/lignin pathways [32, 58, 59]. While AtMYB4/7/32 exhibit canonical EAR sequences (DLNxxP/LxLxL), PtMYB181/182 and AtMYBL2 display INLDL, IxIxL, and DLNIGL, respectively [13, 27, 29]. In the present study, a phylogenetic tree of R2R3-MYB SG4 was reconstructed across representative species spanning basal angiosperms (*Nymphaea colorata*), monocots (*Phalaenopsis equestris* and *Oryza sativa*), and dicots (Arabidopsis and *C.* ×*morifolium*) (Fig. S8A). Interestingly, phylogenetic conservation analysis identified a conserved substitution of the first leucine (L) residue with isoleucine (I) specifically in Asteraceae species (Fig. S8B). Accordingly, CmMYB4 carries this variant (INLEL), suggesting functional conservation within SG4.

‘AtMYB4-type’ repressors typically suppress late-stage anthocyanin biosynthesis by targeting *ANS*, *DFR*, and *UFGT* promoters [33, 60]. Our Y1H and DLR assays demonstrated that CmMYB4 directly binds to and represses promoters of LBGs (*CmDFR*, *CmUFGT*, and *Cm3MaT*), aligning with established models. CmMYB4 may also regulate upstream phenylpropanoid genes, such as *PAL* (phenylalanine ammonia lyase), *C4H* (cinnamate-4-hydroxylase), and *4CL* (4-coumarate: CoA ligase), warranting further investigation.

Subgroup IIIf bHLH proteins (e.g. TT8/EGL3/GL3 orthologs) mediate flavonoid biosynthesis through MBW complexes [12, 61]. The interactions involve the R3 domain of MYB and the MYB-interacting region of bHLH. In this study, we conducted a preliminary exploration of these potential co-regulators (CmbHLH2, CmbHLH24, and CmMYB6). Phylogenetically, CmbHLH2 clusters with SGIIIf-AN1 members (AtTT8, PhAN1, and MdbHLH3), whereas CmbHLH24 aligns with SGIVb clade (OsbHLH063, AtbHLH67, and AtbHLH121) (Fig. S9). While CmMYB6 interacts with CmbHLH2 [62], CmMYB4 specifically associates with CmbHLH24 (Fig. S10). However, this interaction did not significantly alter *CmUFGT* promoter activity in DLR assays, suggesting alternative regulatory mechanisms.

R2R3-MYB repressors employ dual strategies: (i) direct promoter binding via repression motifs or (ii) competitive inhibition by sequestering bHLH cofactors [6, 30]. The absence of CmMYB4-CmbHLH2 interaction and its direct promoter binding capacity suggests CmMYB4 functions as an active repressor rather than a passive competitor. Supporting this, overexpression of *CmMYB4* counteracted CmMYB6-mediated activation of *CmUFGT*, implying competition for *cis*-element binding as a key regulatory mechanism of anthocyanin biosynthesis (Fig. S10).

### Ethylene-induced CmERF3 suppresses anthocyanin biosynthesis through transcriptional activation of CmMYB4

ERF TFs exhibit dual regulatory roles in anthocyanin biosynthesis across species, functioning as either activators or repressors under environmental stimuli (e.g. low temperature, blue light, and ethylene) [18, 63, 64]. The activation of anthocyanin biosynthesis by ERFs principally involves two mechanisms: (i) transcriptional regulation of MYB factors or (ii) physical interaction with MYB proteins to modulate the expression of structural genes . For instance, in apple, MdERF38 enhances drought-induced anthocyanin biosynthesis by interacting with MdMYB1 and facilitating the binding of MdMYB1 to its target genes [37]; MdERF1B promotes anthocyanin and PA accumulation by directly binding to the promoters of *MdMYB9* and *MdMYB11* for their transcriptional activation, in addition to protein-level interaction [48]; similar mechanisms were also found in the studies of *Zanthoxylum bungeanum*, mulberry (*Morus alba*), and litchi [51, 65, 66]. Notably, ERFs may also directly target structural genes [51, 67], demonstrating functional versatility.

The ethylene-mediated suppression of anthocyanin biosynthesis was elucidated in pear and lily. In pear, PyERF105 mediates ethylene-dependent anthocyanin suppression via induction of PyMYB140 repressor [41], while PyERF4.1/4.2 decreases the stability of the activator PybHLH3−PyMYB114−PyERF3 complex by interacting with PyERF3, thereby repressing the transcription of *PyANS* [53]. In lily, ethylene-induced LhERF4 negatively regulates anthocyanin biosynthesis by repressing the activator LhMYBSPLATTER [38]. These repressive mechanisms stand in contrast to the promotive roles of ERFs in other species, collectively demonstrating the species-specific complexity of ethylene-mediated regulation of anthocyanin accumulation.

Our transcriptomic and Y1H screening identified CmERF3 as a candidate anthocyanin repressor in chrysanthemum ray florets by activating CmMYB4 repressor. Structural characterization revealed conserved AP2 domain residues (A14/D19), confirming it belongs to the ERF family. Phylogenetically, CmERF3 clusters with ERF-B1 TFs (e.g. AtERF4/7/8/9/11) containing the EAR motif. Although they repress gene expression in most cases, the opposite result was recently found in Arabidopsis and *L. kaempferi* [38, 68, 69]. AtERF11 in the ERF-B1 subgroup has been shown to specifically bind to the GCC box of the *BT4* promoter to activate its transcription and enhance resistance against *Pseudomonas syringae*, demonstrating their dual functional capacity [69]. Moreover, in *L. kaempferi*, ERF-B1 subgroup member LkERF6 was recently confirmed to directly interact with the promoters of *LkSOD*, *LkCCS*, and *LkCAT* to activate their transcriptions and enhance drought and salt tolerance in tobacco [68].

In this study, Y1H and DLR assays showed that CmERF3 not only activates the expression of *CmMYB4* but also directly represses the structural genes *CmDFR*, *CmUFGT*, and *Cm3MaT*. These findings demonstrate that ERF3 regulates both regulatory and structural genes within the anthocyanin pathway, in agreement with results from prior research [51]. However, the molecular basis for this target-specific switch between repression and activation remains elusive. We hypothesize that this phenomenon may result from the involvement of specific co-activators or co-repressors, or sequence differences in the *cis*-elements that CmERF3 binds to. In addition, differences in chromatin state or epigenetic modifications at the promoter regions of these target genes may also contribute to gene-specific activation or repression. These findings highlight the need for further investigation into the context-dependent dual functions of CmERF3 in transcriptional regulation.

In addition to transcriptional regulation, ethylene-mediated control of anthocyanin biosynthesis may also involve histone acetylation and deacetylation [47, 70]. A notable example is provided by the pear fruit, where PpERF9 recruits the co-repressor PpTPL1 to form a repressive complex. This complex reduces histone acetylation in the promoters of its target genes, *PpRAP2.4* and *PpMYB114*, thereby suppressing their expression. Given that PpMYB114 is a positive regulator of anthocyanin biosynthesis and is itself activated by PpRAP2.4, the PpERF9−PpRAP2.4−PpMYB114 cascade collectively represses anthocyanin accumulation [70]. In this study, CmERF3 appears to modulate the expression of both regulatory and structural genes at the transcriptional level. However, whether histone modifications similarly influence anthocyanin biosynthesis in chrysanthemum remains to be determined and warrants further investigation.

## Conclusion

**Figure 6:**
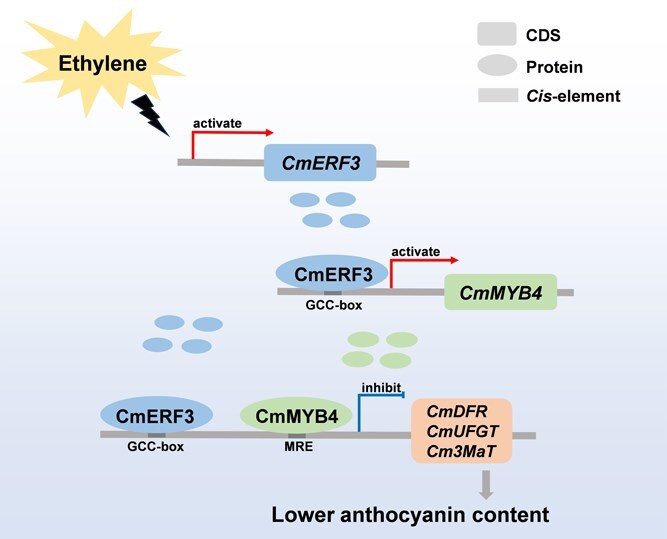
Proposed model for ethylene-mediated suppression of anthocyanin biosynthesis in chrysanthemum ray florets. Ethylene-induced CmERF3 activates the expression of the repressor *CmMYB4*, and together with CmMYB4, directly suppresses the expression of *CmDFR*, *CmUFGT*, and *Cm3MaT*, thereby inhibiting anthocyanin accumulation. ERF, ethylene response factor; DFR, dihydroflavonol 4-reductase; UFGT, UDP-glucose: flavonoid 3-*O*-glucosyltransferase; 3MaT, anthocyanin 3-*O*-glucoside-6″-*O*-malonyltransferase.

In summary, our results indicate that CmERF3 and CmMYB4 participate in the ethylene-mediated suppression of anthocyanin biosynthesis in chrysanthemum ray florets. Ethylene upregulates *CmERF3* expression, which in turn directly activates CmMYB4 by binding to its promoter. CmMYB4 functions as a transcriptional repressor of anthocyanin biosynthesis by directly inhibiting the expression of *CmDFR*, *CmUFGT*, and *Cm3MaT*. In addition, CmERF3 can also bind directly to the promoters of these structural genes to repress their transcription, thereby further reducing anthocyanin accumulation. Collectively, these findings reveal that ethylene inhibits anthocyanin biosynthesis through a CmERF3−CmMYB4−LBGs regulatory module (Fig. 6). This work fills a key knowledge gap linking ethylene signaling with specialized anthocyanin biosynthesis and provides new perspectives for engineering ornamental traits in chrysanthemum.

## Materials and methods

### Plant materials and treatments

Controlled environment chambers at Beijing Forestry University (Beijing, China) were maintained at a photosynthetic photon flux density of 50 ± 5 μmol·m^−2^·s^−1^, temperature of 22 ± 2°C, and relative humidity of 60%. Chrysanthemum (*C.* ×*morifolium* ‘Riqie Taohong’) plants were initially subjected to a 16-h light/8-h dark photoperiod for vegetative growth and then shifted to a 12-h light/12-h dark cycle to induce reproductive development. Capitulum morphogenesis was divided into five stages (S1–S5) as described by Sun et al. [71]. Ray florets were collected at each stage, flash-frozen in liquid nitrogen, and stored at −80°C for subsequent molecular analyses. Tobacco plants were grown under the same long-day conditions (16-h light/8-h dark) and used for transient expression assays 4 weeks after germination.

At the stage when ray florets first emerged from the bracts and remained completely achromatic, capitula were treated with ethephon at concentrations of 0, 100, 200, 300, and 400 mg/L. Each treatment included five biological replicates. Immersion was carried out for 5 min in solutions supplemented with 0.01% (v/v) Tween-20, with deionized water used as the negative control. Based on phenotypic assessment, the 200 mg/L treatment, which elicited the most pronounced response, was selected for further analysis. Capitula from this group were collected across developmental stages S2 to S4 for qRT-PCR, with three technical replicates performed per biological sample.

### Gene isolation and phylogenetic analyses

Using the Quick RNA Isolation Kit (Huayueyang Biotechnology Co., Ltd., Beijing, China), total RNA was purified from the ray florets of *C.* ×*morifolium* ‘Riqie Taohong’. First-strand cDNA was subsequently synthesized with M-MLV Reverse Transcriptase (Promega, Madison, WI, USA) in accordance with the supplier’s instructions. The full-length coding sequences (CDSs) of *CmMYB4* and *CmERF3* were obtained via PCR amplification employing gene-specific primers (Table S1). Each 20 μl reaction mixture contained 14.4 μl ddH₂O, 2 μl 10× buffer (Mg^2+^), 0.4 μl dNTP (10 μM each), 0.3 μl Taq DNA polymerase (5 U/μL), 1 μl each of forward and reverse primers (10 μM), and 1 μl template cDNA. The thermal cycling protocol consisted of an initial denaturation at 94°C for 5 min; 32 cycles of 94°C for 50 s, 58°C for 35 s, and 72°C for 50 s; and a final extension at 72°C for 60 s.

Phylogenetic and sequence analyses were conducted using multiple bioinformatics tools. A phylogenetic tree was constructed in MEGA v6.0 [72] via the neighbor-joining method, employing the Jones-Taylor-Thornton model, pairwise deletion for gaps, and 1000 bootstrap replicates. Homologous protein sequences for CmMYB4 and CmERF3 were obtained through BLASTP searches of the NCBI database (accession numbers in Table S2). Multiple sequence alignment was carried out with DNAMAN v7.0 using default CLUSTALW parameters. Conserved MYB TF motifs were identified with MEME Suite v5.5.2 (maximum motif width = 50; maximum number of motifs = 10). Domain architectures were annotated with Pfam and visualized using TBtools v2.0 [73].

### Subcellular localization

The CDS of *CmMYB4* (excluding the stop codon) was amplified and inserted into the *Xba*I/*Kp*nI sites of the pCAMBIA1302 vector via homologous recombination, generating the *35S::CmMYB4–GFP* fusion construct. Primer information is provided in Table S1. After sequence verification, the recombinant plasmid and empty vector control were introduced into *Agrobacterium tumefaciens* strain GV3101 (carrying the pSoup-p19 helper plasmid) by electroporation. Transformants were selected on LB medium supplemented with 50 μg/mlrifampicin and 50 μg/ml kanamycin.

For transient expression, agrobacterial cells were harvested, resuspended to an OD_600_ of 1.0 in infiltration buffer (10 mM MES, 10 mM MgCl₂, 150 μM acetosyringone, pH 5.6), and infiltrated into the abaxial surface of leaves from 4-week-old *Nicotiana benthamiana* plants using a needleless syringe. Infiltrated plants were kept at 24°C for 48 h in low-light conditions before GFP observation GFP fluorescence was visualized with a Leica TCS SP8 confocal microscope (Leica Camera AG, Wetzlar, Germany); chloroplast autofluorescence was captured in the 650-750-nm channel.

### Yeast one-hybrid assay

For the yeast one-hybrid (Y1H) assays, the full-length CDSs of *CmMYB4* and *CmERF3* were inserted into the pGADT7 prey vector at the *Eco*R1/*Xho*I sites, yielding AD-CmMYB4 and AD-CmERF3. Upstream promoter fragments of *CmDFR* (1076 bp), *CmUFGT* (702 bp), *Cm3MaT* (474 bp), and *CmMYB4* (2052 bp) were independently cloned into the pAbAi bait vector to generate pAbAi::*proCmDFR*, pAbAi::*proCmUFGT*, pAbAi::*proCm3MaT*, and pAbAi::*proCmMYB4*. Each bait construct was linearized and integrated into the Y1H Gold yeast genome via homologous recombination.

To determine the baseline sensitivity of the bait strains, transformants were grown on SD/-Ura medium supplemented with an aureobasidin A (AbA) concentration gradient (0–900 ng/mL); the minimum inhibitory concentration was recorded for each promoter-bait strain. For protein–DNA interaction testing, AD-CmMYB4, AD-CmERF3, or the empty AD vector (negative control) was individually introduced into the bait strains and selected on SD/-Leu medium containing AbA at the predetermined inhibitory concentration. All primer sequences used for plasmid construction are listed in Table S1.

### Dual-luciferase reporter assay

For dual-luciferase assays, the CDSs of *CmMYB4*, *CmbHLH24*, *CmMYB6*, and *CmERF3* were individually inserted into the *Kpn*I*/Sal*I sites of the pGreenII 62-SK effector vector; the empty vector served as the negative control. Promoter fragments of *CmDFR*, *CmUFGT*, *Cm3MaT*, and *CmMYB4* were fused into the *Hind*III/*Bam*HI sites of the pGreenII 0800-LUC reporter vector. All primer sequences used for constructing these plasmids are provided in Table S1.

Verified constructs were introduced into *A. tumefaciens* strain GV3101 (pSoup-p19) via electroporation. Transformants were grown to mid-log phase (OD_600_= 0.6–0.8) in LB medium containing 50 μg/ml rifampicin and 50 μg/ml kanamycin. Bacterial cells were harvested by centrifugation (4000× g, 10 min) and resuspended in infiltration buffer (10 mM MES, 10 mM MgCl₂, 150 μM acetosyringone, pH 5.6). For co-infiltration, effector and reporter strains were combined at a 10:1 (v/v) ratio and pressure-infiltrated into the abaxial epidermis of leaves from 4-week-old *N. benthamiana* plants using a needleless syringe.

After 72 h, firefly luciferase (LUC) and *Renilla* luciferase (REN) activities were quantified using the Dual-Luciferase® Reporter Assay System (Promega) on an EnVision® 2105 Multimode Plate Reader (PerkinElmer, Waltham, MA, USA). Transcriptional activity was expressed as the LUC/REN ratio to normalize for transformation efficiency. Six independent biological replicates (each from a separate infiltration event) were performed per combination, and all values were normalized relative to the empty SK vector control.

### Electrophoretic mobility shift assay

The CDS of *CmMYB4* was cloned into the pET32a vector harboring an N-terminal His-tag to generate the recombinant construct. The CmMYB4-His fusion protein was expressed *in vitro* via prokaryotic induction. For the EMSA, biotin-labeled DNA probes were prepared by annealing complementary oligonucleotides bearing 3′ biotin modifications under the following conditions: 95°C for 5 min, followed by 70°C for 20 min. The binding reaction was performed by incubating the fusion protein with the labeled probes at 24°C for 30 min. Unlabeled probes were included in competition assays, while a mutant probe in which the CAACAG motif was replaced with TTTTTT served as a negative control. Protein–DNA complexes were resolved from free probes by nondenaturing polyacrylamide gel electrophoresis. The sequences of all probes used are provided in Table S1.

### Yeast two-hybrid assay

The CDSs of *CmMYB4*, *CmMYB6*, *CmbHLH2*, and *CmbHLH24* were directionally cloned into the pGADT7 (prey) and pGBKT7 (bait) vectors using *Eco*RI/*Sal*I restriction sites, generating AD- and BD-fusion constructs, respectively. Bait–prey combinations were co-transformed into the yeast two-hybrid (Y2H) Gold strain via the lithium acetate–polyethylene glycol method. Transformants were selected on the SD/-Leu-Trp medium at 30°C for 72 h.

For interaction validation, colonies were replica-plated onto: (i) high-stringency medium: SD/-Leu/-Trp/-Ade/-His +300 ng/mL AbA and (ii) α-Galactosidase assay medium: SD/-Leu/-Trp/-Ade/-His/AbA +80 μg/mL X-α-gal. Positive controls (AD-T + BD-53) and negative controls (AD-T + BD-lam) were included to validate system performance. Protein–protein interactions were confirmed if colonies exhibited: (i) robust growth on high-stringency medium or (ii) distinct blue pigmentation on X-α-gal medium. The primer sequences used for vector construction are listed in Table S1.

### Genetic transformation of chrysanthemum and tobacco

The CDSs of *CmMYB4* and *CmERF3* were individually cloned into the pBI121 binary vector using restriction enzyme-based digestion and ligation. The resulting recombinant plasmids were subsequently introduced into *A. tumefaciens* strain EHA105 via freeze–thaw transformation. For chrysanthemum transformation, transverse thin cell layer explants (5 × 5 mm) were aseptically excised from tissue-cultured seedlings of ‘Riqie Taohong’ and inoculated with the *Agrobacterium* suspension following our established protocol. Putative transgenic plantlets were selected on medium containing 50 mg/L kanamycin. Genomic DNA was extracted from resistant plantlets using a Rapid Plant Genomic DNA Isolation Kit (Sangon Biotech, Shanghai, China), and transgene integration was confirmed by qRT-PCR with gene-specific primers (Table S1). Verified T₀ transgenic plants were vegetatively propagated and acclimatized in a controlled greenhouse under the following conditions: 25 ± 2°C, a 16/8-h photoperiod, and 65% relative humidity. Tobacco transformation was performed as previously described [54].

### qRT-PCR

Transcript abundance analysis was performed using SYBR® Premix Ex Taq™ II (Takara, Shiga, Japan)-based qRT-PCR on a Mini Option Real-time PCR system (Bio-Rad, Hercules, CA, USA). Relative quantification was calculated using the comparative 2^−ΔΔCT^ method [74] with protein phosphatase 2A (*CmPP2A*) serving as the validated reference gene. Primer sequences are listed in Table S1.

### Anthocyanin extraction and quantification

Anthocyanin extraction was conducted on ray florets collected from four distinct capitulum developmental stages (S2−S5) using an acidified methanol solvent system (methanol:deionized water:formic acid:trifluoroacetic acid = 70:27:2:1, v/v/v/v) under light-protected conditions, as previously optimized [75]. Following centrifugation at 12 000× *g* for 15 min at 4°C, supernatants were sequentially filtered through 0.22-μm nylon membrane filters (ANPEL Laboratory Technologies, Shanghai, China) and degassed via ultrasonic treatment (40 kHz, 10 min) prior to HPLC analysis.

Chromatographic separation was achieved using an Agilent 1100 Series HPLC System equipped with a ZORBAX SB-C18 column (4.6 × 250 mm, 5 μm; Agilent Technologies, Santa Clara, CA, USA). The mobile phase consisted of (A) 1% formic acid in water and (B) acetonitrile, delivered at 0.8 mL/min with gradient elution: 0–5 min 5%–10% B, 5–20 min 10%–25% B, 20%–25 min 25%–5% B. Anthocyanins were quantified at 515 nm using rutin (≥94% purity) as an external standard, with calibration curves established over six concentration points (0.1–50 μg/mL, *R*^2^ > 0.995).

Each sample run for HPLC was repeated three times under the same conditions. Data acquisition and processing were performed using ChemStation software (vB.04.03) following the validated protocol of Lin and Harnly [76], with peak area normalization for differential compound quantification.

### Transcriptomic analysis

RNA sequencing libraries (*n* = 48) were prepared by BioMarker Technologies Co., Ltd. (Beijing, China) using the NEBNext® Ultra™ II RNA Library Prep Kit (New England Biolabs Inc., Ipswich, MA, USA) encompassing three vegetative tissues (root, stem, and leaf) and ray florets from five capitulum developmental stages (S1-S5) of both transgenic and CK plants. Paired-end sequencing (2 × 150 bp) was performed on the Illumina NovaSeq 6000 platform (Illumina Inc., San Diego, CA, USA), generating ~40 million reads per library with Q30 > 85% quality scores.

Raw reads were preprocessed through Trimmomatic v0.39 [77] to remove adapters and low-quality bases. High-quality clean reads were aligned to the *C.* ×*morifolium* reference genome [78] using HISAT2 v2.1.0 [79] with alignment rates >85%. Transcript abundance was quantified as fragments per kilobase million values through StringTie v2.1.3 [80] in reference-guided mode.

Differential expression analysis was conducted using DESeq2 v1.24.0 [81] with biological replicates (*n* = 3 per group), applying Benjamini-Hochberg correction for multiple testing. Genes meeting |log_2_ fold change| ≥ 1 and adjusted *P*-value (padj) < 0.01 were classified as differentially expressed genes (DEGs).

### TO-GCNs

To elucidate the temporal regulatory dynamics of anthocyanin biosynthesis, a TO-GCN analysis was constructed [82], incorporating RNA-seq datasets from ray florets across five developmental stages (S1-S5) of both transgenic and CK plants. A *MYB* gene designated *C.* ×*morifolium 1819207*, exhibiting peak expression at S1 followed by progressive downregulation, was selected to generate TO-GCNs by MFselector [83] with default Markov blanket parameters.

The breadth-first search (BSF) algorithm assigned temporal hierarchy levels to TFs within the major GCN component. DEGs were categorized into TFs and non-TFs, and the Pearson's correlation coefficient (PCC) values between each pair of TF and non-TF genes were calculated, retaining significant correlations (FDR-adjusted *p* < 0.05, |PCC| > 0.9) for network construction using Cytoscape v3.9.1 [84], which comprises 38 curated genes, including chlorophyll metabolism , carotenoid biosynthesis , and anthocyanin pathway components.

The *CmMYB4* (Gene ID: 175740)-centric regulatory module was extracted using MCODE clustering (score > 8) [85]. Promoter analysis of *CmMYB4* involved: (i) retrieving 2-kb upstream genomic sequence from the *C.* ×*morifolium* genome, (ii) *cis*-element prediction via PlantCARE [86] with E-value < 1e^−5^, and (iii) visualization of spatial element distribution using TBtools v2.023 [73] with default motif mapping parameters.

### Statistical analysis

Statistical analysis was performed using Student’s t-test and one-way ANOVA followed by Duncan’s multiple range test in SPSS 27 software (IBM, Armonk, NY, USA). Asterisks and different letters indicate significant differences.

## Supplementary Material

Web_Material_uhag132

## Data Availability

RNA-seq data in this study are publicly available in the NCBI database under accession number PRJNA1289674.
