# Supplementary material for: Ethylene-responsive CmERF3−CmMYB4 cascade suppresses anthocyanin biosynthesis in Chrysanthemum ×morifolium
Source: Hortic Res. 2026 Apr 7;13(8):uhag132. doi: 10.1093/hr/uhag132 (PMC13437082; doi:10.1093/hr/uhag132)
Supplement: Supplementary file 1 [file web_material_uhag132.zip › Supplementary Data.docx]

**Supplemental Data**

**Figure S1.** Relative expression levels of anthocyanin biosynthetic genes in different plant materials and treatment conditions. (**A**) *CmMYB4*-overexpressing lines. (**B**) *CmERF3*-overexpressing lines. (C) Chrysanthemum ray florets treated with 200 mg·L⁻¹ ethephon. Asterisks indicate significantly different values (**p* < 0.05, ***p* < 0.01, ****p* < 0.001, *****p* < 0.0001).

**Figure S2.** GO annotation of the TFs in subnetworks. The ethylene-activated signaling pathway is marked with black dots. ME, molecular function; CC, cellular component; BP, biological process.

**Figure S3.** Expression profiles of ethylene biosynthesis and signaling pathway genes.

**Figure S4.** The temporal and spatial expression patterns of *CmERF3*.

**Figure S5.** Subcellular localization of CmERF3 in tobacco leaf epidermal cells.

**Figure S6.** (**A**) Phenotypes of 'Aoshuang Fencai' at different developmental stages under 0 or 200 mg·L⁻¹ ethephon treatment. (**B**) Total anthocyanin content in ray florets treated with 200 mg·L⁻¹ ethephon. (**C**) Temporal expression profiles of *CmERF3*, *CmMYB4*, and anthocyanin biosynthetic genes in response to 200 mg·L⁻¹ ethephon treatment.

**Figure S7.** The dual luciferase assays in *N. benthamiana* leaves showed that CmERF3 activates the *CmMYB4* promoter.

**Figure S8.** Conservation analysis of EAR Motif in Asteraceae and non-Asteraceae plants. (**A**) Phylogeny of R2R3-MYB subgroup 4 across basal angiosperms (*Nymphaea colorata*), monocots (*Phalaenopsis equestris* and *Oryza sativa*), and eudicots (*Arabidopsis thaliana* and *C.* ×*morifolium*). (**B**) Conserved motif architecture showing the phylogenetically constrained substitution of the first leucine (L) with isoleucine (I) in the EAR motif of Asteraceae species.

**Figure S9.** Phylogenetic analysis of CmbHLH2 and CmbHLH24.

**Figure S10.** (**A**) Y2H assays showing protein−protein interactions between CmMYB4 and regulatory partners CmbHLH2, CmbHLH24, and CmMYB6. (**B**) Regulatory dynamics of CmMYB4 on *CmUFGT* promoter activity under co-expression with CmbHLH24 or CmMYB6.

**Table S1.** List of primers.

| **Purpose** | **Primer Name** | **Primer Sequence (5'−3')** |
| --- | --- | --- |
| **Gene isolation** | CmMYB4 | F: ATGGTGAGATCACCTTGTTGTG |
|  |  | R: TCATCTCCATCTACTCGACAAG |
|  | CmERF3 | F: ATGGCGAAAAAGTCTACAGCA |
|  |  | R: TTATAAGCACAAAGCGGTGAC |
| **Subcellular localization** | CmMYB4-GFP | F: GGGGGACTCTTACCATGGCAATGGTGAGATCACCTTGT |
|  |  | R: CTAGTCAGATCTACCATGGCTCTCCATCTACTCGACAA |
|  | CmERF3-GFP | F: ATTTACGAACGATACTCGAGATGGCGAAAAAGTCTACAGCA |
|  |  | R: ACCATCACTAGTACGTCGACTAAGCACAAAGCGGTGACAA |
| **Yeast one-hybrid assay** | CmDFRpro-AbAi | F: TGAATTCGAGCTCGGTACCAGCGTATCTAGTGGTGTTG |
|  |  | R: ACAGAGCACATGCCTCGAGAGGAGGGTTGCATGCAACG |
|  | CmUFGTpro-AbAi | F: TGAATTCGAGCTCGGTACCAATGAGAAATAAATGTGGT |
|  |  | R: ACAGAGCACATGCCTCGAGGTAGATTTGCCATTTAAAC |
|  | Cm3MaTpro-AbAi | F: TGAATTCGAGCTCGGTACCAAATAGAAACAACCATTCA |
|  |  | R: ACAGAGCACATGCCTCGAGTTGGATGTGGTTGTGTTAA |
|  | CmMYB4pro-AbAi | F: GCTTGAATTCGAGCTCGGTACCTTTTAGTAAATGGCTCGCC |
|  |  | R: GGTCGACAGATCCCCGGGTACCATGGAAGGAAAGGATGGAT |
|  | pAbAi | F: TTCAAGGAAACCGAAATCA |
|  |  | R: TATGCTACAAAGGACCTAAT |
|  | AD-CmMYB4 | F: GGAATTCCATATGATGGTGAGATCACCTTGT |
|  |  | R: CGAGCTCGTCATCTCCATCTACTCGA |
|  | AD-CmERF3 | F: GCCATGGAGGCCAGTGAATTCATGGCGAAAAAGTCTACAGCA |
|  |  | R: CAGCTCGAGCTCGATGGATCCTTATAAGCACAAAGCGGTGAC |
| **EMSA** | pET32a-CmMYB4 | F: CCGCACAAACGCCGTTGATTTTTTGACG |
|  |  | R: CGTCAAAAAATCAACGGCGTTTGTGCGG |
|  | Probe 1 | F: CCGCACAAACGCCGTTGATTTTTTGACG |
|  |  | R: CGTCAAAAAATCAACGGCGTTTGTGCGG |
|  | Probe 2 | F: ATTTTAGTTTGGTTGTATTAACG |
|  |  | R: CGTTAATACAACCAAACTAAAAT |
|  | Probe 3 | F: AAGGTATTTACTGTTGTGCCTAATA |
|  |  | R: TATTAGGCACAACAGTAAATACCTT |
| **Dual luciferase assay** | CmDFRpro-LUC | F: GAGGTCGACGGTATCGATAAGCTTAGTAACGTCCGCACAAAC |
|  |  | R: GGCCGCTCTAGAACTAGTGGATCCGTTGTTTTAAGCTTGTGGT |
|  | CmUFGTpro-LUC | F: GAGGTCGACGGTATCGATAAGCTTTCAATTAAGAAAGATGTTA |
|  |  | R: GGCCGCTCTAGAACTAGTGGATCCTTGGCTGTATTTTAAGAAA |
|  | Cm3MaTpro-LUC | F: GAGGTCGACGGTATCGATAAGCTTAAAGTTGAATACGTGTTAA |
|  |  | R: GGCCGCTCTAGAACTAGTGGATCCTGAATAATTTGACAATGGT |
|  | CmMYB4pro-LUC | F: AGGTCGACGGTATCGATAAGCTTTTTTAGTAAATGGCTCGCCC |
|  |  | R: CGCTCTAGAACTAGTGGATCCTTTTTTTGTTTTTTGGTTTCAAAG  ATGGAAGG |
|  | SK-CmMYB4 | F: GGCCGCTCTAGAACTAGTGGATCCATGGTGAGATCACCTTGTT |
|  |  | R: GAGGTCGACGGTATCGATAAGCTTTCATCTCCATCTACTCGAC |
|  | SK-CmbHLH24 | F:GGCCGCTCTAGAACTAGTGGATCCATGGAGGGTTCGGATGAA |
|  |  | R:AGGTCGACGGTATCGATAAGCTTTCATTAAGCTGCTCTTGACT |
|  | SK-CmMYB6 | F: AGAGGACAGCCCAAGCTGAGCTCATGAGACCGAGTAGTAGTA |
|  |  | R: AGGTCGACGGTATCGATAAGCTTTCATAGTTGGTCCGAATTT |
|  | SK-CmERF3 | F: TCCCCCGGGCTGCAGGAATTCATGGCGAAAAAGTCTACAG |
|  |  | R: GTCGACGGTATCGATAAGCTTTTATAAGCACAAAGCGGTGA |
|  | pGreenII 62-SK | F: CCTTCGCAAGACCCTTCCTC |
|  |  | R: CGACTTAGTTTACCCGCCAATA |
|  | pGreenII 0800-LUC | F: GCCAGGGTTTTCCCAGTCAC |
|  |  | R: TCCAGGAACCAGGGCGTATC |
| **Yeast two-hybrid assay** | AD-CmMYB4 | F: GGAATTCCATATGATGGTGAGATCACCTTGT |
|  |  | R: CGAGCTCGTCATCTCCATCTACTCGA |
|  | AD-CmMYB6 | F: GGAATTCCATATGATGAGACCGAGTAGTAGT |
|  |  | R: CGAGCTCGTCATAGTTGGTCCGAATT |
|  | AD-CmbHLH2 | F: GCCATGGAGGCCAGTGAATTCATGGCTGCCAGCGGACCACC |
|  |  | R: CAGCTCGAGCTCGATGGATCCCTAAGGAGATATTATTTGG |
|  | AD-CmbHLH24 | F: GCCATGGAGGCCAGTGAATTCATGGAGGGTTCGGATGAA |
|  |  | R: CAGCTCGAGCTCGATGGATCCCTAAGGAGATATTATTTGGTTG |
|  | BD-CmMYB4 | F: ATGGCCATGGAGGCCGAATTCATGAGACCGAGTAGTAGT |
|  |  | R: CCGCTGCAGGTCGACGGATCCTCATAGTTGGTCCGAATT |
|  | BD-CmMYB6 | F: ATGGCCATGGAGGCCGAATTCATGAGACCGAGTAGTAGTACAG |
|  |  | R: CCGCTGCAGGTCGACGGATCCTCATAGTTGGTCCGAATTTAAA |
|  | BD-CmbHLH2 | F: ATGGCCATGGAGGCCGAATTCATGGCTGCCAGCGGACCACC |
|  |  | R: CCGCTGCAGGTCGACGGATCCCTAAGGAGATATTATTTGG |
|  | BD-CmbHLH24 | F: ATGGCCATGGAGGCCGAATTCATGGAGGGTTCGGATGAA |
|  |  | R: CCGCTGCAGGTCGACGGATCCCTAAGGAGATATTATTTGGTTG |
| **Genetic transformation** | pBI121-CmMYB4 | F: CCACTATCCTTCGCAAGACC |
|  |  | R: CACTGGTGTCTCCTCAATGG |
|  | pBI121-CmERF3 | F: ACGACTCAATGACAAGAAGAAAAT |
|  |  | R: AAATATTCATCGTCAACAACAGAA |
| **Quantitative real-time PCR** | CmCHS1 | F: AGGGAAGTCGGGCTAACATT |
|  |  | R: AAGCTCAACCTGGTCCAGAA |
|  | CmCHS2 | F: CTTCGCCCTTCAGTTAAACG |
|  |  | R: TCTGGCCCACGAAAAGTTAC |
|  | CmCHI | F: CTACACACATTTGCCCGATG |
|  |  | R: GCTTGTGGGAAAACACCATT |
|  | CmF3H | F: AGGAGAAACTCCGGTTCGAT |
|  |  | R: CCAAGCCCATTAGATCCTCA |
|  | CmF3'H | F: TTCATGTTCGGTTCGATTCA |
|  |  | R: TCGGCTATTGCCCATTCTAC |
|  | CmDFR | F: AGGTTGCCATGGAGTGTTTC |
|  |  | R: TCGCTCCAGTGTGACTCATC |
|  | CmANS | F: CCCAAAAACACGCAAGAAAT |
|  |  | R: TTTCTCCTCGACTGGCTGAT |
|  | CmUFGT | F: CATTTCCCCCAAGGATTTTT |
|  |  | R: GGGACCCCAACTCCAATACT |
|  | Cm3MaT | F: TGGATCACTCCCGGTTTTAG |
|  |  | R: TGTTGGGATTTGGGTAAGGA |
|  | CmMYB4 | F: TGCTAACAATTCCACCACCA |
|  |  | R: TTGCCTGTTGCTTACGACTG |
|  | CmERF3 | F: CCAATCGGATCAAGACAGGT |
|  |  | R: AAACTCGGGCACTTGATGAG |
|  | CmPP2A | F: TGTCAGCCATCTGTAAAATGTGCG |
|  |  | R: CGTGGGTCCTCAGAATCAAATAAGT |

**Table S2.** Accession numbers for phylogenetic tree construction.

| **Protein Name** | **Accession Number** | **Protein Name** | **Accession Number** |
| --- | --- | --- | --- |
| CmMYB4 | PX620089 | PpMYB18 | ALO81021.1 |
| AtMYB6 | NP_192684.1 | PtMYB165 | AXY05276.1 |
| AtMYB7 | NP_179263.1 | PtMYB182 | AJI76863 |
| AtMYB8 | Q9SDS8.1 | PvMYB4a | AEM17348 |
| AtMYB32 | NP_195225.1 | RcMYB4 | USU43561.1 |
| FaMYB1 | AAK84064.1 | SmMYB4 | ADG46002.1 |
| GhMYB6 | AAN28286.1 | TaMYB1 | ABC86569.1 |
| HvMYB1 | P20026 | VvMYB4a | ABL61515.1 |
| MdMYB16 | ADL36756.1 | VvMYB4b | ACN94269.1 |
| MtMYB2 | XP_003616388 | VvMYBC2-L1 | ABW34393 |
| NtMYB2 | ATO58377.1 | ZmMYB31 | CAJ42202.1 |
| PgMYB1 | AJD79907.1 |  |  |
| CmERF3 | PX620090 | AtRAP2.11 | AAC49777.1 |
| AtCRF10 | NP_177022 | GmERF3 | NP_001238300.2 |
| AtERF1 | NP_567530.4 | GmERF4 | NP_001238595.2 |
| AtERF11 | NP_174159.2 | GmERF5 | NP_001304530.1 |
| AtERF12 | NP_174158.1 | GmERF6 | NP_001236636.1 |
| AtERF14 | NP_171932.1 | LeERF1 | Q84XB3.1 |
| AtERF15 | NP_850162.1 | LeERF3 | AAO34705.1 |
| AtERF3 | NP_175479.1 | LhERF4 | QYI40124.2 |
| AtERF4 | NP_188139.1 | LkERF6 | WNG73270.1 |
| AtERF5 | NP_568679.1 | NbERF3 | BAJ72664.1 |
| AtERF7 | NP_188666.1 | Pti5 | NP_001233987.1 |
| AtERF8 | NP_175725.1 | Pti6 | NP_001233991.1 |
| AtRAP2.2 | NP_566482.1 | TaERF3 | ABQ52687.1 |
| AtRAP2.3 | NP_188299.1 | TaERF4 | XP_044320986.1 |
| AtRAP2.6 | AAC49772.1 | Tsi1 | AAC14323.1 |

**Table S3.** Construction of a Time-Ordered Gene Co-expression Regulatory Network.

| **Gene ID1** | **Coexpression Type** | **Gene ID2** | **PCC under C1** | **PCC under C2** | **TF Type** | **Level** | **Group** | |
| --- | --- | --- | --- | --- | --- | --- | --- | --- |
| PH02Gene32056 | C1+C2+ | PH02Gene00063 | 0.968487 | 0.969204 | HMG | 1 | 1 | |
| PH02Gene32056 | C1+C2+ | PH02Gene00074 | 0.995959 | 0.968248 | HMG | 1 | 1 | |
| PH02Gene32056 | C1+C2+ | PH02Gene00150 | 0.999266 | 0.998564 | HMG | 1 | 1 | |
| PH02Gene32056 | C1+C2+ | PH02Gene00151 | 0.982353 | 0.998915 | HMG | 1 | 1 | |
| PH02Gene32056 | C1+C2+ | PH02Gene00152 | 0.977859 | 0.999074 | HMG | 1 | 1 | |
| PH02Gene32056 | C1+C2+ | PH02Gene00536 | 0.973264 | 0.990896 | HMG | 1 | 1 | |
| PH02Gene32056 | C1+C2+ | PH02Gene00625 | 0.960435 | 0.962362 | HMG | 1 | 1 | |
| PH02Gene32056 | C1+C2+ | PH02Gene00691 | 0.946571 | 0.933492 | HMG | 1 | 1 | |
| PH02Gene32056 | C1+C2+ | PH02Gene00801 | 0.964069 | 0.972729 | HMG | 1 | 1 | |
| PH02Gene32056 | C1+C2+ | PH02Gene01004 | 0.979797 | 0.996541 | HMG | 1 | 1 | |
| PH02Gene32056 | C1+C2+ | PH02Gene01086 | 0.997873 | 0.993017 | HMG | 1 | 1 | |
| PH02Gene32056 | C1+C2+ | PH02Gene01144 | 0.986733 | 0.964943 | HMG | 1 | 1 | |
| PH02Gene32056 | C1+C2+ | PH02Gene01243 | 0.962432 | 0.957884 | HMG | 1 | 1 | |
| PH02Gene32056 | C1+C2+ | PH02Gene01286 | 0.964346 | 0.976324 | HMG | 1 | 1 | |
| PH02Gene32056 | C1+C2+ | PH02Gene01457 | 0.955187 | 0.935509 | HMG | 1 | 1 | |
| PH02Gene32056 | C1+C2+ | PH02Gene01458 | 0.99951 | 0.965033 | HMG | 1 | 1 | |
| PH02Gene32056 | C1+C2+ | PH02Gene01637 | 0.987587 | 0.992577 | HMG | 1 | 1 | |
| PH02Gene32056 | C1+C2+ | PH02Gene01814 | 0.936873 | 0.967957 | HMG | 1 | 1 | |
| PH02Gene32056 | C1+C2+ | PH02Gene02015 | 0.99019 | 0.954113 | HMG | 1 | 1 | |
| PH02Gene32056 | C1+C2+ | PH02Gene02095 | 0.991895 | 0.982336 | HMG | 1 | 1 | |
| PH02Gene32056 | C1+C2+ | PH02Gene02211 | 0.945345 | 0.942838 | HMG | 1 | 1 | |
| PH02Gene32056 | C1+C2+ | PH02Gene02272 | 0.96924 | 0.998987 | HMG | 1 | 1 | |
| PH02Gene32056 | C1+C2+ | PH02Gene02300 | 0.999488 | 0.993642 | HMG | 1 | 1 | |
| PH02Gene32056 | C1+C2+ | PH02Gene02352 | 0.99828 | 0.984425 | HMG | 1 | 1 | |
| PH02Gene32056 | C1+C2+ | PH02Gene02370 | 0.98687 | 0.934621 | HMG | 1 | 1 | |
| PH02Gene32056 | C1+C2+ | PH02Gene02429 | 0.969989 | 0.966862 | HMG | 1 | 1 | |
| PH02Gene32056 | C1+C2+ | PH02Gene02542 | 0.944516 | 0.982241 | HMG | 1 | 1 | |
| PH02Gene32056 | C1+C2+ | PH02Gene02746 | 0.98728 | 0.975847 | HMG | 1 | 1 | |
| PH02Gene32056 | C1+C2+ | PH02Gene02890 | 0.979434 | 0.989785 | HMG | 1 | 1 | |
| PH02Gene32056 | C1+C2+ | PH02Gene02977 | 0.987532 | 0.934907 | HMG | 1 | 1 | |
| PH02Gene32056 | C1+C2+ | PH02Gene03048 | 0.973372 | 0.940637 | HMG | 1 | 1 | |
| PH02Gene32056 | C1+C2+ | PH02Gene03085 | 0.938183 | 0.975855 | HMG | 1 | 1 | |
| PH02Gene32056 | C1+C2+ | PH02Gene03110 | 0.989765 | 0.994424 | HMG | 1 | 1 | |
| PH02Gene32056 | C1+C2+ | PH02Gene03150 | 0.983198 | 0.940339 | HMG | 1 | 1 | |
| PH02Gene32056 | C1+C2+ | PH02Gene03188 | 0.954317 | 0.994034 | HMG | 1 | 1 | |
| PH02Gene32056 | C1+C2+ | PH02Gene03369 | 0.998592 | 0.994864 | HMG | 1 | 1 | |
| PH02Gene32056 | C1+C2+ | PH02Gene03411 | 0.963932 | 0.983196 | HMG | 1 | 1 | |
| PH02Gene32056 | C1+C2+ | PH02Gene03778 | 0.97307 | 0.998769 | HMG | 1 | 1 | |
| PH02Gene32056 | C1+C2+ | PH02Gene03782 | 0.968424 | 0.941633 | HMG | 1 | 1 | |
| PH02Gene32056 | C1+C2+ | PH02Gene03915 | 0.947835 | 0.963225 | HMG | 1 | 1 | |
| PH02Gene32056 | C1+C2+ | PH02Gene03946 | 0.997782 | 0.985219 | HMG | 1 | 1 | |
| PH02Gene32056 | C1+C2+ | PH02Gene03997 | 0.98621 | 0.968207 | HMG | 1 | 1 | |
| PH02Gene32056 | C1+C2+ | PH02Gene03998 | 0.948777 | 0.9985 | HMG | 1 | 1 | |
| PH02Gene32056 | C1+C2+ | PH02Gene04026 | 0.984747 | 0.987278 | HMG | 1 | 1 | |
| PH02Gene32056 | C1+C2+ | PH02Gene04185 | 0.943084 | 0.973673 | HMG | 1 | 1 | |
| PH02Gene32056 | C1+C2+ | PH02Gene04199 | 0.985403 | 0.993202 | HMG | 1 | 1 | |
| PH02Gene32056 | C1+C2+ | PH02Gene04451 | 0.948298 | 0.964972 | HMG | 1 | 1 | |
| PH02Gene32056 | C1+C2+ | PH02Gene04678 | 0.95474 | 0.988122 | HMG | 1 | 1 | |
| PH02Gene32056 | C1+C2+ | PH02Gene05516 | 0.945047 | 0.990246 | HMG | 1 | 1 | |
| PH02Gene32056 | C1+C2+ | PH02Gene05606 | 0.973186 | 0.996694 | HMG | 1 | 1 | |
| PH02Gene32056 | C1+C2+ | PH02Gene05680 | 0.981832 | 0.94995 | HMG | 1 | 1 | |
| PH02Gene32056 | C1+C2+ | PH02Gene05832 | 0.954564 | 0.989625 | HMG | 1 | 1 | |
| PH02Gene32056 | C1+C2+ | PH02Gene05856 | 0.960949 | 0.958312 | HMG | 1 | 1 | |
| PH02Gene32056 | C1+C2+ | PH02Gene05859 | 0.96221 | 0.999414 | HMG | 1 | 1 | |
| PH02Gene32056 | C1+C2+ | PH02Gene05885 | 0.962315 | 0.970873 | HMG | 1 | 1 | |
| PH02Gene32056 | C1+C2+ | PH02Gene06261 | 0.974772 | 0.998304 | HMG | 1 | 1 | |
| PH02Gene32056 | C1+C2+ | PH02Gene06288 | 0.993458 | 0.969591 | HMG | 1 | 1 | |
| PH02Gene32056 | C1+C2+ | PH02Gene06429 | 0.996715 | 0.993758 | HMG | 1 | 1 | |
| PH02Gene32056 | C1+C2+ | PH02Gene06475 | 0.98343 | 0.944091 | HMG | 1 | 1 | |
| PH02Gene32056 | C1+C2+ | PH02Gene06600 | 0.996256 | 0.960226 | HMG | 1 | 1 | |
| PH02Gene32056 | C1+C2+ | PH02Gene06676 | 0.987979 | 0.981616 | HMG | 1 | 1 | |
| PH02Gene32056 | C1+C2+ | PH02Gene07046 | 0.988443 | 0.954418 | HMG | 1 | 1 | |
| PH02Gene32056 | C1+C2+ | PH02Gene07363 | 0.994859 | 0.982089 | HMG | 1 | 1 | |
| PH02Gene32056 | C1+C2+ | PH02Gene07388 | 0.949692 | 0.979173 | HMG | 1 | 1 | |
| PH02Gene32056 | C1+C2+ | PH02Gene07715 | 0.961649 | 0.968018 | HMG | 1 | 1 | |
| PH02Gene32056 | C1+C2+ | PH02Gene07862 | 0.953767 | 0.976511 | HMG | 1 | 1 | |
| PH02Gene32056 | C1+C2+ | PH02Gene07980 | 0.965 | 0.973393 | HMG | 1 | 1 | |
| PH02Gene32056 | C1+C2+ | PH02Gene08351 | 0.965965 | 0.980376 | HMG | 1 | 1 | |
| PH02Gene32056 | C1+C2+ | PH02Gene08646 | 0.956011 | 0.966011 | HMG | 1 | 1 | |
| PH02Gene32056 | C1+C2+ | PH02Gene08767 | 0.97636 | 0.990238 | HMG | 1 | 1 | |
| PH02Gene32056 | C1+C2+ | PH02Gene08864 | 0.953001 | 0.943159 | HMG | 1 | 1 | |
| PH02Gene32056 | C1+C2+ | PH02Gene09109 | 0.988569 | 0.972623 | HMG | 1 | 1 | |
| PH02Gene32056 | C1+C2+ | PH02Gene09228 | 0.942756 | 0.983132 | HMG | 1 | 1 | |
| PH02Gene32056 | C1+C2+ | PH02Gene09322 | 0.971432 | 0.954485 | HMG | 1 | 1 | |
| PH02Gene32056 | C1+C2+ | PH02Gene09619 | 0.965052 | 0.954283 | HMG | 1 | 1 | |
| PH02Gene32056 | C1+C2+ | PH02Gene09910 | 0.957472 | 0.991892 | HMG | 1 | 1 | |
| PH02Gene32056 | C1+C2+ | PH02Gene09927 | 0.960344 | 0.985983 | HMG | 1 | 1 | |
| PH02Gene32056 | C1+C2+ | PH02Gene10822 | 0.996724 | 0.993257 | HMG | 1 | 1 | |
| PH02Gene32056 | C1+C2+ | PH02Gene11895 | 0.938464 | 0.930399 | HMG | 1 | 1 | |
| PH02Gene32056 | C1+C2+ | PH02Gene12177 | 0.998076 | 0.997165 | HMG | 1 | 1 | |
| PH02Gene32056 | C1+C2+ | PH02Gene12241 | 0.945104 | 0.949244 | HMG | 1 | 1 | |
| PH02Gene32056 | C1+C2+ | PH02Gene12303 | 0.99938 | 0.949222 | HMG | 1 | 1 | |
| PH02Gene32056 | C1+C2+ | PH02Gene12354 | 0.966013 | 0.951924 | HMG | 1 | 1 | |
| PH02Gene32056 | C1+C2+ | PH02Gene12380 | 0.98119 | 0.980891 | HMG | 1 | 1 | |
| PH02Gene32056 | C1+C2+ | PH02Gene12582 | 0.993794 | 0.938216 | HMG | 1 | 1 | |
| PH02Gene32056 | C1+C2+ | PH02Gene12711 | 0.995367 | 0.98024 | HMG | 1 | 1 | |
| PH02Gene32056 | C1+C2+ | PH02Gene12786 | 0.997202 | 0.990112 | HMG | 1 | 1 | |
| PH02Gene32056 | C1+C2+ | PH02Gene12916 | 0.97116 | 0.949892 | HMG | 1 | 1 | |
| PH02Gene32056 | C1+C2+ | PH02Gene12939 | 0.947478 | 0.936084 | HMG | 1 | 1 | |
| PH02Gene32056 | C1+C2+ | PH02Gene12945 | 0.951874 | 0.951331 | HMG | 1 | 1 | |
| PH02Gene32056 | C1+C2+ | PH02Gene13025 | 0.941721 | 0.939024 | HMG | 1 | 1 | |
| PH02Gene32056 | C1+C2+ | PH02Gene13427 | 0.968337 | 0.995601 | HMG | 1 | 1 | |
| PH02Gene32056 | C1+C2+ | PH02Gene13650 | 0.944301 | 0.991634 | HMG | 1 | 1 | |
| PH02Gene32056 | C1+C2+ | PH02Gene13700 | 0.956726 | 0.972765 | HMG | 1 | 1 | |
| PH02Gene32056 | C1+C2+ | PH02Gene13779 | 0.984594 | 0.969538 | HMG | 1 | 1 | |
| PH02Gene32056 | C1+C2+ | PH02Gene13965 | 0.976035 | 0.998304 | HMG | 1 | 1 | |
| PH02Gene32056 | C1+C2+ | PH02Gene14283 | 0.969796 | 0.981898 | HMG | 1 | 1 | |
| PH02Gene32056 | C1+C2+ | PH02Gene14386 | 0.978596 | 0.966217 | HMG | 1 | 1 | |
| PH02Gene32056 | C1+C2+ | PH02Gene14575 | 0.998795 | 0.998956 | HMG | 1 | 1 | |
| PH02Gene32056 | C1+C2+ | PH02Gene14584 | 0.991436 | 0.99609 | HMG | 1 | 1 | |
| PH02Gene32056 | C1+C2+ | PH02Gene14657 | 0.98646 | 0.997657 | HMG | 1 | 1 | |
| PH02Gene32056 | C1+C2+ | PH02Gene14658 | 0.955982 | 0.964116 | HMG | 1 | 1 | |
| PH02Gene32056 | C1+C2+ | PH02Gene14662 | 0.995694 | 0.998859 | HMG | 1 | 1 | |
| PH02Gene32056 | C1+C2+ | PH02Gene14753 | 0.965402 | 0.976385 | HMG | 1 | 1 | |
| PH02Gene32056 | C1+C2+ | PH02Gene14796 | 0.997173 | 0.97608 | HMG | 1 | 1 | |
| PH02Gene32056 | C1+C2+ | PH02Gene14859 | 0.990909 | 0.97405 | HMG | 1 | 1 | |
| PH02Gene32056 | C1+C2+ | PH02Gene14947 | 0.984345 | 0.966995 | HMG | 1 | 1 | |
| PH02Gene32056 | C1+C2+ | PH02Gene15071 | 0.947211 | 0.951753 | HMG | 1 | 1 | |
| PH02Gene32056 | C1+C2+ | PH02Gene15117 | 0.975948 | 0.932723 | HMG | 1 | 1 | |
| PH02Gene32056 | C1+C2+ | PH02Gene15334 | 0.947066 | 0.947431 | HMG | 1 | 1 | |
| PH02Gene32056 | C1+C2+ | PH02Gene15346 | 0.935181 | 0.971559 | HMG | 1 | 1 | |
| PH02Gene32056 | C1+C2+ | PH02Gene15459 | 0.998473 | 0.99661 | HMG | 1 | 1 | |
| PH02Gene32056 | C1+C2+ | PH02Gene15557 | 0.979138 | 0.970625 | HMG | 1 | 1 | |
| PH02Gene32056 | C1+C2+ | PH02Gene16131 | 0.951423 | 0.93891 | HMG | 1 | 1 | |
| PH02Gene32056 | C1+C2+ | PH02Gene16196 | 0.996724 | 0.939081 | HMG | 1 | 1 | |
| PH02Gene32056 | C1+C2+ | PH02Gene16344 | 0.990049 | 0.97544 | HMG | 1 | 1 | |
| PH02Gene32056 | C1+C2+ | PH02Gene16414 | 0.981651 | 0.971005 | HMG | 1 | 1 | |
| PH02Gene32056 | C1+C2+ | PH02Gene16432 | 0.970693 | 0.96597 | HMG | 1 | 1 | |
| PH02Gene32056 | C1+C2+ | PH02Gene16610 | 0.969821 | 0.933508 | HMG | 1 | 1 | |
| PH02Gene32056 | C1+C2+ | PH02Gene16623 | 0.962627 | 0.936277 | HMG | 1 | 1 | |
| PH02Gene32056 | C1+C2+ | PH02Gene16705 | 0.982928 | 0.932884 | HMG | 1 | 1 | |
| PH02Gene32056 | C1+C2+ | PH02Gene16957 | 0.965101 | 0.951825 | HMG | 1 | 1 | |
| PH02Gene32056 | C1+C2+ | PH02Gene17224 | 0.981662 | 0.998927 | HMG | 1 | 1 | |
| PH02Gene32056 | C1+C2+ | PH02Gene17539 | 0.995685 | 0.994599 | HMG | 1 | 1 | |
| PH02Gene32056 | C1+C2+ | PH02Gene17607 | 0.941609 | 0.974709 | HMG | 1 | 1 | |
| PH02Gene32056 | C1+C2+ | PH02Gene17803 | 0.979118 | 0.962141 | HMG | 1 | 1 | |
| PH02Gene32056 | C1+C2+ | PH02Gene17807 | 0.953418 | 0.999312 | HMG | 1 | 1 | |
| PH02Gene32056 | C1+C2+ | PH02Gene17848 | 0.963609 | 0.938385 | HMG | 1 | 1 | |
| PH02Gene32056 | C1+C2+ | PH02Gene17855 | 0.967052 | 0.990427 | HMG | 1 | 1 | |
| PH02Gene32056 | C1+C2+ | PH02Gene18047 | 0.937382 | 0.93452 | HMG | 1 | 1 | |
| PH02Gene32056 | C1+C2+ | PH02Gene18199 | 0.94892 | 0.989089 | HMG | 1 | 1 | |
| PH02Gene32056 | C1+C2+ | PH02Gene18256 | 0.963134 | 0.977524 | HMG | 1 | 1 | |
| PH02Gene32056 | C1+C2+ | PH02Gene18276 | 0.982326 | 0.99522 | HMG | 1 | 1 | |
| PH02Gene32056 | C1+C2+ | PH02Gene18347 | 0.976542 | 0.946198 | HMG | 1 | 1 | |
| PH02Gene32056 | C1+C2+ | PH02Gene18414 | 0.984219 | 0.989349 | HMG | 1 | 1 | |
| PH02Gene32056 | C1+C2+ | PH02Gene18558 | 0.953606 | 0.934749 | HMG | 1 | 1 | |
| PH02Gene32056 | C1+C2+ | PH02Gene18663 | 0.994916 | 0.999457 | HMG | 1 | 1 | |
| PH02Gene32056 | C1+C2+ | PH02Gene18690 | 0.9714 | 0.962866 | HMG | 1 | 1 | |
| PH02Gene32056 | C1+C2+ | PH02Gene18737 | 0.996862 | 0.999929 | HMG | 1 | 1 | |
| PH02Gene32056 | C1+C2+ | PH02Gene18753 | 0.994567 | 0.978735 | HMG | 1 | 1 | |
| PH02Gene32056 | C1+C2+ | PH02Gene18763 | 0.980049 | 0.986377 | HMG | 1 | 1 | |
| PH02Gene32056 | C1+C2+ | PH02Gene18848 | 0.956324 | 0.96897 | HMG | 1 | 1 | |
| PH02Gene32056 | C1+C2+ | PH02Gene18942 | 0.979839 | 0.938911 | HMG | 1 | 1 | |
| PH02Gene32056 | C1+C2+ | PH02Gene18944 | 0.971213 | 0.991279 | HMG | 1 | 1 | |
| PH02Gene32056 | C1+C2+ | PH02Gene19245 | 0.996123 | 0.990721 | HMG | 1 | 1 | |
| PH02Gene32056 | C1+C2+ | PH02Gene19544 | 0.983125 | 0.992531 | HMG | 1 | 1 | |
| PH02Gene32056 | C1+C2+ | PH02Gene19549 | 0.982739 | 0.980502 | HMG | 1 | 1 | |
| PH02Gene32056 | C1+C2+ | PH02Gene19787 | 0.958903 | 0.939092 | HMG | 1 | 1 | |
| PH02Gene32056 | C1+C2+ | PH02Gene19819 | 0.99649 | 0.999826 | HMG | 1 | 1 | |
| PH02Gene32056 | C1+C2+ | PH02Gene20031 | 0.957444 | 0.991701 | HMG | 1 | 1 | |
| PH02Gene32056 | C1+C2+ | PH02Gene20135 | 0.979303 | 0.989928 | HMG | 1 | 1 | |
| PH02Gene32056 | C1+C2+ | PH02Gene20156 | 0.984158 | 0.938112 | HMG | 1 | 1 | |
| PH02Gene32056 | C1+C2+ | PH02Gene20576 | 0.95613 | 0.955222 | HMG | 1 | 1 | |
| PH02Gene32056 | C1+C2+ | PH02Gene20881 | 0.987241 | 0.991895 | HMG | 1 | 1 | |
| PH02Gene32056 | C1+C2+ | PH02Gene20969 | 0.938095 | 0.996307 | HMG | 1 | 1 | |
| PH02Gene32056 | C1+C2+ | PH02Gene21093 | 0.940466 | 0.93595 | HMG | 1 | 1 | |
| PH02Gene32056 | C1+C2+ | PH02Gene21199 | 0.946879 | 0.980295 | HMG | 1 | 1 | |
| PH02Gene32056 | C1+C2+ | PH02Gene21374 | 0.999151 | 0.952304 | HMG | 1 | 1 | |
| PH02Gene32056 | C1+C2+ | PH02Gene21550 | 0.983087 | 0.935015 | HMG | 1 | 1 | |
| PH02Gene32056 | C1+C2+ | PH02Gene21590 | 0.997876 | 0.998114 | HMG | 1 | 1 | |
| PH02Gene32056 | C1+C2+ | PH02Gene21744 | 0.940911 | 0.984342 | HMG | 1 | 1 | |
| PH02Gene32056 | C1+C2+ | PH02Gene22095 | 0.96844 | 0.962593 | HMG | 1 | 1 | |
| PH02Gene32056 | C1+C2+ | PH02Gene22265 | 0.973551 | 0.996195 | HMG | 1 | 1 | |
| PH02Gene32056 | C1+C2+ | PH02Gene22337 | 0.967848 | 0.988101 | HMG | 1 | 1 | |
| PH02Gene32056 | C1+C2+ | PH02Gene22734 | 0.995106 | 0.9814 | HMG | 1 | 1 | |
| PH02Gene32056 | C1+C2+ | PH02Gene23033 | 0.992816 | 0.97452 | HMG | 1 | 1 | |
| PH02Gene32056 | C1+C2+ | PH02Gene23069 | 0.969718 | 0.952243 | HMG | 1 | 1 | |
| PH02Gene32056 | C1+C2+ | PH02Gene23239 | 0.975538 | 0.959266 | HMG | 1 | 1 | |
| PH02Gene32056 | C1+C2+ | PH02Gene23391 | 0.979863 | 0.979435 | HMG | 1 | 1 | |
| PH02Gene32056 | C1+C2+ | PH02Gene23425 | 0.990304 | 0.99631 | HMG | 1 | 1 | |
| PH02Gene32056 | C1+C2+ | PH02Gene23437 | 0.968436 | 0.938264 | HMG | 1 | 1 | |
| PH02Gene32056 | C1+C2+ | PH02Gene23622 | 0.984904 | 0.986189 | HMG | 1 | 1 | |
| PH02Gene32056 | C1+C2+ | PH02Gene23898 | 0.998772 | 0.998766 | HMG | 1 | 1 | |
| PH02Gene32056 | C1+C2+ | PH02Gene24034 | 0.934631 | 0.969861 | HMG | 1 | 1 | |
| PH02Gene32056 | C1+C2+ | PH02Gene24077 | 0.984389 | 0.997649 | HMG | 1 | 1 | |
| PH02Gene32056 | C1+C2+ | PH02Gene24244 | 0.970748 | 0.984256 | HMG | 1 | 1 | |
| PH02Gene32056 | C1+C2+ | PH02Gene24353 | 0.963568 | 0.97326 | HMG | 1 | 1 | |
| PH02Gene32056 | C1+C2+ | PH02Gene24469 | 0.994516 | 0.998304 | HMG | 1 | 1 | |
| PH02Gene32056 | C1+C2+ | PH02Gene24511 | 0.983834 | 0.977338 | HMG | 1 | 1 | |
| PH02Gene32056 | C1+C2+ | PH02Gene24633 | 0.983997 | 0.996279 | HMG | 1 | 1 | |
| PH02Gene32056 | C1+C2+ | PH02Gene24787 | 0.991268 | 0.998146 | HMG | 1 | 1 | |
| PH02Gene32056 | C1+C2+ | PH02Gene25081 | 0.990883 | 0.992985 | HMG | 1 | 1 | |
| PH02Gene32056 | C1+C2+ | PH02Gene25386 | 0.999527 | 0.996539 | HMG | 1 | 1 | |
| PH02Gene32056 | C1+C2+ | PH02Gene25561 | 0.964375 | 0.962779 | HMG | 1 | 1 | |
| PH02Gene32056 | C1+C2+ | PH02Gene25665 | 0.940851 | 0.979205 | HMG | 1 | 1 | |
| PH02Gene32056 | C1+C2+ | PH02Gene25851 | 0.958789 | 0.948135 | HMG | 1 | 1 | |
| PH02Gene32056 | C1+C2+ | PH02Gene25985 | 0.967943 | 0.959657 | HMG | 1 | 1 | |
| PH02Gene32056 | C1+C2+ | PH02Gene26055 | 0.953086 | 0.966353 | HMG | 1 | 1 | |
| PH02Gene32056 | C1+C2+ | PH02Gene26217 | 0.938733 | 0.95071 | HMG | 1 | 1 | |
| PH02Gene32056 | C1+C2+ | PH02Gene26318 | 0.953131 | 0.94283 | HMG | 1 | 1 | |
| PH02Gene32056 | C1+C2+ | PH02Gene26381 | 0.965277 | 0.958487 | HMG | 1 | 1 | |
| PH02Gene32056 | C1+C2+ | PH02Gene26414 | 0.949512 | 0.969504 | HMG | 1 | 1 | |
| PH02Gene32056 | C1+C2+ | PH02Gene26502 | 0.933366 | 0.934683 | HMG | 1 | 1 | |
| PH02Gene32056 | C1+C2+ | PH02Gene26525 | 0.959512 | 0.956154 | HMG | 1 | 1 | |
| PH02Gene32056 | C1+C2+ | PH02Gene26568 | 0.983691 | 0.93376 | HMG | 1 | 1 | |
| PH02Gene32056 | C1+C2+ | PH02Gene26736 | 0.970738 | 0.943409 | HMG | 1 | 1 | |
| PH02Gene32056 | C1+C2+ | PH02Gene26764 | 0.973582 | 0.999191 | HMG | 1 | 1 | |
| PH02Gene32056 | C1+C2+ | PH02Gene27423 | 0.96267 | 0.994724 | HMG | 1 | 1 | |
| PH02Gene32056 | C1+C2+ | PH02Gene27570 | 0.978356 | 0.998765 | HMG | 1 | 1 | |
| PH02Gene32056 | C1+C2+ | PH02Gene27763 | 0.977562 | 0.963674 | HMG | 1 | 1 | |
| PH02Gene32056 | C1+C2+ | PH02Gene28048 | 0.951118 | 0.988001 | HMG | 1 | 1 | |
| PH02Gene32056 | C1+C2+ | PH02Gene28264 | 0.981988 | 0.939444 | HMG | 1 | 1 | |
| PH02Gene32056 | C1+C2+ | PH02Gene28961 | 0.957429 | 0.979887 | HMG | 1 | 1 | |
| PH02Gene32056 | C1+C2+ | PH02Gene28994 | 0.955782 | 0.950089 | HMG | 1 | 1 | |
| PH02Gene32056 | C1+C2+ | PH02Gene29053 | 0.94346 | 0.983989 | HMG | 1 | 1 | |
| PH02Gene32056 | C1+C2+ | PH02Gene29181 | 0.997989 | 0.996112 | HMG | 1 | 1 | |
| PH02Gene32056 | C1+C2+ | PH02Gene29257 | 0.968027 | 0.945778 | HMG | 1 | 1 | |
| PH02Gene32056 | C1+C2+ | PH02Gene29452 | 0.959665 | 0.996313 | HMG | 1 | 1 | |
| PH02Gene32056 | C1+C2+ | PH02Gene29679 | 0.979195 | 0.974326 | HMG | 1 | 1 | |
| PH02Gene32056 | C1+C2+ | PH02Gene29871 | 0.999356 | 0.999582 | HMG | 1 | 1 | |
| PH02Gene32056 | C1+C2+ | PH02Gene29878 | 0.945187 | 0.982007 | HMG | 1 | 1 | |
| PH02Gene32056 | C1+C2+ | PH02Gene30472 | 0.968248 | 0.933523 | HMG | 1 | 1 | |
| PH02Gene32056 | C1+C2+ | PH02Gene30677 | 0.970956 | 0.952423 | HMG | 1 | 1 | |
| PH02Gene32056 | C1+C2+ | PH02Gene30825 | 0.978855 | 0.935678 | HMG | 1 | 1 | |
| PH02Gene32056 | C1+C2+ | PH02Gene30842 | 0.990558 | 0.953453 | HMG | 1 | 1 | |
| PH02Gene32056 | C1+C2+ | PH02Gene30873 | 0.979356 | 0.945887 | HMG | 1 | 1 | |
| PH02Gene32056 | C1+C2+ | PH02Gene30958 | 0.965967 | 0.97824 | HMG | 1 | 1 | |
| PH02Gene32056 | C1+C2+ | PH02Gene31061 | 0.93253 | 0.985207 | HMG | 1 | 1 | |
| PH02Gene32056 | C1+C2+ | PH02Gene31157 | 0.959859 | 0.95944 | HMG | 1 | 1 | |
| PH02Gene32056 | C1+C2+ | PH02Gene31224 | 0.984392 | 0.990911 | HMG | 1 | 1 | |
| PH02Gene32056 | C1+C2+ | PH02Gene31593 | 0.962015 | 0.990167 | HMG | 1 | 1 | |
| PH02Gene32056 | C1+C2+ | PH02Gene31722 | 0.996384 | 0.989792 | HMG | 1 | 1 | |
| PH02Gene32056 | C1+C2+ | PH02Gene31885 | 0.952501 | 0.983886 | HMG | 1 | 1 | |
| PH02Gene32056 | C1+C2+ | PH02Gene32293 | 0.982622 | 0.966796 | HMG | 1 | 1 | |
| PH02Gene32056 | C1+C2+ | PH02Gene32333 | 0.999673 | 0.993078 | HMG | 1 | 1 | |
| PH02Gene32056 | C1+C2+ | PH02Gene32394 | 0.95063 | 0.990342 | HMG | 1 | 1 | |
| PH02Gene32056 | C1+C2+ | PH02Gene32582 | 0.961484 | 0.991583 | HMG | 1 | 1 | |
| PH02Gene32056 | C1+C2+ | PH02Gene32584 | 0.99034 | 0.942669 | HMG | 1 | 1 | |
| PH02Gene32056 | C1+C2+ | PH02Gene33706 | 0.948068 | 0.98713 | HMG | 1 | 1 | |
| PH02Gene32056 | C1+C2+ | PH02Gene33723 | 0.995542 | 0.998304 | HMG | 1 | 1 | |
| PH02Gene32056 | C1+C2+ | PH02Gene33744 | 0.937346 | 0.961212 | HMG | 1 | 1 | |
| PH02Gene32056 | C1+C2+ | PH02Gene33783 | 0.965223 | 0.989808 | HMG | 1 | 1 | |
| PH02Gene32056 | C1+C2+ | PH02Gene33785 | 0.990866 | 0.937945 | HMG | 1 | 1 | |
| PH02Gene32056 | C1+C2+ | PH02Gene34062 | 0.986394 | 0.983043 | HMG | 1 | 1 | |
| PH02Gene32056 | C1+C2+ | PH02Gene34281 | 0.981415 | 0.982528 | HMG | 1 | 1 | |
| PH02Gene32056 | C1+C2+ | PH02Gene34300 | 0.988504 | 0.933619 | HMG | 1 | 1 | |
| PH02Gene32056 | C1+C2+ | PH02Gene34456 | 0.999323 | 0.998053 | HMG | 1 | 1 | |
| PH02Gene32056 | C1+C2+ | PH02Gene34493 | 0.992761 | 0.983172 | HMG | 1 | 1 | |
| PH02Gene32056 | C1+C2+ | PH02Gene34514 | 0.952292 | 0.94033 | HMG | 1 | 1 | |
| PH02Gene32056 | C1+C2+ | PH02Gene34542 | 0.996485 | 0.998519 | HMG | 1 | 1 | |
| PH02Gene32056 | C1+C2+ | PH02Gene34676 | 0.976584 | 0.981035 | HMG | 1 | 1 | |
| PH02Gene32056 | C1+C2+ | PH02Gene35175 | 0.960642 | 0.999272 | HMG | 1 | 1 | |
| PH02Gene32056 | C1+C2+ | PH02Gene35272 | 0.957276 | 0.974942 | HMG | 1 | 1 | |
| PH02Gene32056 | C1+C2+ | PH02Gene35441 | 0.962715 | 0.970331 | HMG | 1 | 1 | |
| PH02Gene32056 | C1+C2+ | PH02Gene35460 | 0.959979 | 0.991761 | HMG | 1 | 1 | |
| PH02Gene32056 | C1+C2+ | PH02Gene35543 | 0.960011 | 0.996623 | HMG | 1 | 1 | |
| PH02Gene32056 | C1+C2+ | PH02Gene35565 | 0.940498 | 0.959419 | HMG | 1 | 1 | |
| PH02Gene32056 | C1+C2+ | PH02Gene35884 | 0.97094 | 0.938098 | HMG | 1 | 1 | |
| PH02Gene32056 | C1+C2+ | PH02Gene36349 | 0.991341 | 0.994765 | HMG | 1 | 1 | |
| PH02Gene32056 | C1+C2+ | PH02Gene36420 | 0.950887 | 0.998359 | HMG | 1 | 1 | |
| PH02Gene32056 | C1+C2+ | PH02Gene36582 | 0.953703 | 0.961494 | HMG | 1 | 1 | |
| PH02Gene32056 | C1+C2+ | PH02Gene37018 | 0.997705 | 0.985162 | HMG | 1 | 2 | |
| PH02Gene32056 | C1+C2+ | PH02Gene37257 | 0.959963 | 0.932483 | HMG | 1 | 2 | |
| PH02Gene32056 | C1+C2+ | PH02Gene37334 | 0.988411 | 0.980877 | HMG | 1 | 2 | |
| PH02Gene32056 | C1+C2+ | PH02Gene37503 | 0.99259 | 0.998673 | HMG | 1 | 2 | |
| PH02Gene32056 | C1+C2+ | PH02Gene37522 | 0.999722 | 0.994585 | HMG | 1 | 2 | |
| PH02Gene32056 | C1+C2+ | PH02Gene37635 | 0.985892 | 0.999638 | HMG | 1 | 2 | |
| PH02Gene32056 | C1+C2+ | PH02Gene37763 | 0.971252 | 0.998304 | HMG | 1 | 2 | |
| PH02Gene32056 | C1+C2+ | PH02Gene37903 | 0.951572 | 0.934557 | HMG | 1 | 2 | |
| PH02Gene32056 | C1+C2+ | PH02Gene38267 | 0.944507 | 0.943444 | HMG | 1 | 2 | |
| PH02Gene32056 | C1+C2+ | PH02Gene38367 | 0.940664 | 0.990909 | HMG | 1 | 2 | |
| PH02Gene32056 | C1+C2+ | PH02Gene38381 | 0.948935 | 0.973566 | HMG | 1 | 2 | |
| PH02Gene32056 | C1+C2+ | PH02Gene38696 | 0.960849 | 0.97395 | HMG | 1 | 2 | |
| PH02Gene32056 | C1+C2+ | PH02Gene39220 | 0.985503 | 0.99054 | HMG | 1 | 2 | |
| PH02Gene32056 | C1+C2+ | PH02Gene39278 | 0.968097 | 0.990277 | HMG | 1 | 2 | |
| PH02Gene32056 | C1+C2+ | PH02Gene39281 | 0.96501 | 0.967587 | HMG | 1 | 2 | |
| PH02Gene32056 | C1+C2+ | PH02Gene39541 | 0.978757 | 0.972167 | HMG | 1 | 2 | |
| PH02Gene32056 | C1+C2+ | PH02Gene39751 | 0.988948 | 0.951817 | HMG | 1 | 2 | |
| PH02Gene32056 | C1+C2+ | PH02Gene39885 | 0.966413 | 0.958482 | HMG | 1 | 2 | |
| PH02Gene32056 | C1+C2+ | PH02Gene39901 | 0.963333 | 0.94917 | HMG | 1 | 2 | |
| PH02Gene32056 | C1+C2+ | PH02Gene39927 | 0.981597 | 0.961529 | HMG | 1 | 2 | |
| PH02Gene32056 | C1+C2+ | PH02Gene40016 | 0.996888 | 0.98621 | HMG | 1 | 2 | |
| PH02Gene32056 | C1+C2+ | PH02Gene40017 | 0.992027 | 0.990774 | HMG | 1 | 2 | |
| PH02Gene32056 | C1+C2+ | PH02Gene40040 | 0.998855 | 0.999155 | HMG | 1 | 2 | |
| PH02Gene32056 | C1+C2+ | PH02Gene40071 | 0.95273 | 0.971687 | HMG | 1 | 2 | |
| PH02Gene32056 | C1+C2+ | PH02Gene40075 | 0.954904 | 0.9706 | HMG | 1 | 2 | |
| PH02Gene32056 | C1+C2+ | PH02Gene40184 | 0.998639 | 0.954404 | HMG | 1 | 2 | |
| PH02Gene32056 | C1+C2+ | PH02Gene40605 | 0.974974 | 0.952514 | HMG | 1 | 2 | |
| PH02Gene32056 | C1+C2+ | PH02Gene40926 | 0.980308 | 0.97978 | HMG | 1 | 2 | |
| PH02Gene32056 | C1+C2+ | PH02Gene40956 | 0.98608 | 0.944112 | HMG | 1 | 2 | |
| PH02Gene32056 | C1+C2+ | PH02Gene41041 | 0.952758 | 0.960027 | HMG | 1 | 2 | |
| PH02Gene32056 | C1+C2+ | PH02Gene41327 | 0.975004 | 0.987891 | HMG | 1 | 2 | |
| PH02Gene32056 | C1+C2+ | PH02Gene41404 | 0.997464 | 0.991007 | HMG | 1 | 2 | |
| PH02Gene32056 | C1+C2+ | PH02Gene41483 | 0.984731 | 0.942834 | HMG | 1 | 2 | |
| PH02Gene32056 | C1+C2+ | PH02Gene41532 | 0.985385 | 0.939874 | HMG | 1 | 2 | |
| PH02Gene32056 | C1+C2+ | PH02Gene41784 | 0.961761 | 0.986131 | HMG | 1 | 2 | |
| PH02Gene32056 | C1+C2+ | PH02Gene41943 | 0.957703 | 0.954162 | HMG | 1 | 2 | |
| PH02Gene32056 | C1+C2+ | PH02Gene42194 | 0.957075 | 0.944256 | HMG | 1 | 2 | |
| PH02Gene32056 | C1+C2+ | PH02Gene42693 | 0.980768 | 0.970153 | HMG | 1 | 2 | |
| PH02Gene32056 | C1+C2+ | PH02Gene42789 | 0.983319 | 0.973213 | HMG | 1 | 2 | |
| PH02Gene32056 | C1+C2+ | PH02Gene43015 | 0.996746 | 0.998282 | HMG | 1 | 2 | |
| PH02Gene32056 | C1+C2+ | PH02Gene43240 | 0.98297 | 0.988398 | HMG | 1 | 2 | |
| PH02Gene32056 | C1+C2+ | PH02Gene43432 | 0.95147 | 0.94048 | HMG | 1 | 2 | |
| PH02Gene32056 | C1+C2+ | PH02Gene43508 | 0.965179 | 0.972648 | HMG | 1 | 2 | |
| PH02Gene32056 | C1+C2+ | PH02Gene43540 | 0.992856 | 0.995296 | HMG | 1 | 2 | |
| PH02Gene32056 | C1+C2+ | PH02Gene43573 | 0.977587 | 0.961367 | HMG | 1 | 2 | |
| PH02Gene32056 | C1+C2+ | PH02Gene43612 | 0.998109 | 0.981418 | HMG | 1 | 2 | |
| PH02Gene32056 | C1+C2+ | PH02Gene43767 | 0.960342 | 0.994772 | HMG | 1 | 2 | |
| PH02Gene32056 | C1+C2+ | PH02Gene44046 | 0.974351 | 0.948703 | HMG | 1 | 2 | |
| PH02Gene32056 | C1+C2+ | PH02Gene44069 | 0.992083 | 0.996115 | HMG | 1 | 2 | |
| PH02Gene32056 | C1+C2+ | PH02Gene44316 | 0.955066 | 0.948891 | HMG | 1 | 2 | |
| PH02Gene32056 | C1+C2+ | PH02Gene44535 | 0.950454 | 0.983262 | HMG | 1 | 2 | |
| PH02Gene32056 | C1+C2+ | PH02Gene44588 | 0.980681 | 0.983362 | HMG | 1 | 2 | |
| PH02Gene32056 | C1+C2+ | PH02Gene44676 | 0.954289 | 0.986565 | HMG | 1 | 2 | |
| PH02Gene32056 | C1+C2+ | PH02Gene45075 | 0.976705 | 0.98778 | HMG | 1 | 2 | |
| PH02Gene32056 | C1+C2+ | PH02Gene45236 | 0.983589 | 0.956679 | HMG | 1 | 2 | |
| PH02Gene32056 | C1+C2+ | PH02Gene45242 | 0.964728 | 0.966856 | HMG | 1 | 2 | |
| PH02Gene32056 | C1+C2+ | PH02Gene45318 | 0.952674 | 0.966099 | HMG | 1 | 2 | |
| PH02Gene32056 | C1+C2+ | PH02Gene45660 | 0.931129 | 0.987577 | HMG | 1 | 2 | |
| PH02Gene32056 | C1+C2+ | PH02Gene45733 | 0.997449 | 0.99844 | HMG | 1 | 2 | |
| PH02Gene32056 | C1+C2+ | PH02Gene46344 | 0.974502 | 0.971989 | HMG | 1 | 2 | |
| PH02Gene32056 | C1+C2+ | PH02Gene46407 | 0.997511 | 0.989318 | HMG | 1 | 2 | |
| PH02Gene32056 | C1+C2+ | PH02Gene46513 | 0.999826 | 0.998561 | HMG | 1 | 2 | |
| PH02Gene32056 | C1+C2+ | PH02Gene46889 | 0.996757 | 0.998978 | HMG | 1 | 2 | |
| PH02Gene32056 | C1+C2+ | PH02Gene47129 | 0.989528 | 0.999299 | HMG | 1 | 2 | |
| PH02Gene32056 | C1+C2+ | PH02Gene47168 | 0.992283 | 0.98085 | HMG | 1 | 2 | |
| PH02Gene32056 | C1+C2+ | PH02Gene47312 | 0.955435 | 0.968833 | HMG | 1 | 2 | |
| PH02Gene32056 | C1+C2+ | PH02Gene47485 | 0.97354 | 0.996442 | HMG | 1 | 2 | |
| PH02Gene32056 | C1+C2+ | PH02Gene47631 | 0.940327 | 0.970812 | HMG | 1 | 2 | |
| PH02Gene32056 | C1+C2+ | PH02Gene48013 | 0.984005 | 0.98572 | HMG | 1 | 2 | |
| PH02Gene32056 | C1+C2+ | PH02Gene48059 | 0.974049 | 0.934236 | HMG | 1 | 2 | |
| PH02Gene32056 | C1+C2+ | PH02Gene48194 | 0.958269 | 0.997939 | HMG | 1 | 2 | |
| PH02Gene32056 | C1+C2+ | PH02Gene48303 | 0.946822 | 0.990375 | HMG | 1 | 2 | |
| PH02Gene32056 | C1+C2+ | PH02Gene48345 | 0.968455 | 0.978024 | HMG | 1 | 2 | |
| PH02Gene32056 | C1+C2+ | PH02Gene48654 | 0.99525 | 0.998304 | HMG | 1 | 2 | |
| PH02Gene32056 | C1+C2+ | PH02Gene48662 | 0.935999 | 0.968747 | HMG | 1 | 2 | |
| PH02Gene32056 | C1+C2+ | PH02Gene48689 | 0.993105 | 0.969754 | HMG | 1 | 2 | |
| PH02Gene32056 | C1+C2+ | PH02Gene48765 | 0.998997 | 0.973485 | HMG | 1 | 2 | |
| PH02Gene32056 | C1+C2+ | PH02Gene48876 | 0.988054 | 0.998304 | HMG | 1 | 2 | |
| PH02Gene32056 | C1+C2+ | PH02Gene49057 | 0.934651 | 0.968269 | HMG | 1 | 2 | |
| PH02Gene32056 | C1+C2+ | PH02Gene49111 | 0.996845 | 0.994779 | HMG | 1 | 2 | |
| PH02Gene32056 | C1+C2+ | PH02Gene49160 | 0.981771 | 0.958105 | HMG | 1 | 2 | |
| PH02Gene32056 | C1+C2+ | PH02Gene49162 | 0.965775 | 0.931963 | HMG | 1 | 2 | |
| PH02Gene32056 | C1+C2+ | PH02Gene49239 | 0.992671 | 0.977924 | HMG | 1 | 2 | |
| PH02Gene32056 | C1+C2+ | PH02Gene49374 | 0.946709 | 0.992197 | HMG | 1 | 2 | |
| PH02Gene32056 | C1+C2+ | PH02Gene49407 | 0.964596 | 0.962308 | HMG | 1 | 2 | |
| PH02Gene32056 | C1+C2+ | PH02Gene49708 | 0.963892 | 0.967045 | HMG | 1 | 3 | |
| PH02Gene32056 | C1+C2+ | PH02Gene49729 | 0.974719 | 0.981401 | HMG | 1 | 3 | |
| PH02Gene32056 | C1+C2+ | PH02Gene50597 | 0.959996 | 0.961475 | HMG | 1 | 3 | |
| PH02Gene32056 | C1+C2+ | PH02Gene51342 | 0.965266 | 0.998611 | HMG | 1 | 3 | |
| PH02Gene32056 | C1+C2+ | novel.10141 | 0.941652 | 0.93154 | HMG | 1 | 3 | |
| PH02Gene32056 | C1+C2+ | novel.10150 | 0.979583 | 0.954663 | HMG | 1 | 3 | |
| PH02Gene32056 | C1+C2+ | novel.10190 | 0.946309 | 0.992285 | HMG | 1 | 3 | |
| PH02Gene32056 | C1+C2+ | novel.1033 | 0.999336 | 0.999294 | HMG | 1 | 3 | |
| PH02Gene32056 | C1+C2+ | novel.10350 | 0.943393 | 0.990841 | HMG | 1 | 3 | |
| PH02Gene32056 | C1+C2+ | novel.10411 | 0.939503 | 0.951219 | HMG | 1 | 3 | |
| PH02Gene32056 | C1+C2+ | novel.10485 | 0.955982 | 0.984986 | HMG | 1 | 3 | |
| PH02Gene32056 | C1+C2+ | novel.10533 | 0.983651 | 0.998304 | HMG | 1 | 3 | |
| PH02Gene32056 | C1+C2+ | novel.10880 | 0.985374 | 0.998836 | HMG | 1 | 3 | |
| PH02Gene32056 | C1+C2+ | novel.10928 | 0.949712 | 0.969481 | HMG | 1 | 3 | |
| PH02Gene32056 | C1+C2+ | novel.1152 | 0.999783 | 0.937734 | HMG | 1 | 3 | |
| PH02Gene32056 | C1+C2+ | novel.11797 | 0.992328 | 0.939033 | HMG | 1 | 3 | |
| PH02Gene32056 | C1+C2+ | novel.12033 | 0.981718 | 0.998304 | HMG | 1 | 3 | |
| PH02Gene32056 | C1+C2+ | novel.12035 | 0.977274 | 0.986167 | HMG | 1 | 3 | |
| PH02Gene32056 | C1+C2+ | novel.12720 | 0.939449 | 0.97011 | HMG | 1 | 3 | |
| PH02Gene32056 | C1+C2+ | novel.12721 | 0.97647 | 0.989269 | HMG | 1 | 3 | |
| PH02Gene32056 | C1+C2+ | novel.12879 | 0.998348 | 0.994029 | HMG | 1 | 3 | |
| PH02Gene32056 | C1+C2+ | novel.13262 | 0.95217 | 0.953639 | HMG | 1 | 3 | |
| PH02Gene32056 | C1+C2+ | novel.13297 | 0.964648 | 0.988037 | HMG | 1 | 3 | |
| PH02Gene32056 | C1+C2+ | novel.13434 | 0.964292 | 0.967614 | HMG | 1 | 3 | |
| PH02Gene32056 | C1+C2+ | novel.13734 | 0.969316 | 0.964412 | HMG | 1 | 3 | |
| PH02Gene32056 | C1+C2+ | novel.13840 | 0.985446 | 0.971485 | HMG | 1 | 3 | |
| PH02Gene32056 | C1+C2+ | novel.14186 | 0.938798 | 0.994034 | HMG | 1 | 3 | |
| PH02Gene32056 | C1+C2+ | novel.14391 | 0.999871 | 0.972383 | HMG | 1 | 3 | |
| PH02Gene32056 | C1+C2+ | novel.14445 | 0.951371 | 0.932777 | HMG | 1 | 3 | |
| PH02Gene32056 | C1+C2+ | novel.14502 | 0.942279 | 0.946252 | HMG | 1 | 3 | |
| PH02Gene32056 | C1+C2+ | novel.15376 | 0.95192 | 0.981873 | HMG | 1 | 3 | |
| PH02Gene32056 | C1+C2+ | novel.15420 | 0.997894 | 0.959211 | HMG | 1 | 3 | |
| PH02Gene32056 | C1+C2+ | novel.16019 | 0.94335 | 0.936944 | HMG | 1 | 3 | |
| PH02Gene32056 | C1+C2+ | novel.2444 | 0.943522 | 0.969026 | HMG | 1 | 3 | |
| PH02Gene32056 | C1+C2+ | novel.2475 | 0.994288 | 0.973454 | HMG | 1 | 3 | |
| PH02Gene32056 | C1+C2+ | novel.3209 | 0.94094 | 0.948545 | HMG | 1 | 3 | |
| PH02Gene32056 | C1+C2+ | novel.3261 | 0.980727 | 0.954019 | HMG | 1 | 3 | |
| PH02Gene32056 | C1+C2+ | novel.3457 | 0.992068 | 0.961 | HMG | 1 | 3 | |
| PH02Gene32056 | C1+C2+ | novel.3720 | 0.970604 | 0.950526 | HMG | 1 | 3 | |
| PH02Gene32056 | C1+C2+ | novel.3862 | 0.984586 | 0.930261 | HMG | 1 | 3 | |
| PH02Gene32056 | C1+C2+ | novel.4162 | 0.988157 | 0.963314 | HMG | 1 | 3 | |
| PH02Gene32056 | C1+C2+ | novel.4842 | 0.983815 | 0.972277 | HMG | 1 | 3 | |
| PH02Gene32056 | C1+C2+ | novel.5503 | 0.943013 | 0.986735 | HMG | 1 | 3 | |
| PH02Gene32056 | C1+C2+ | novel.578 | 0.963785 | 0.958973 | HMG | 1 | 3 | |
| PH02Gene32056 | C1+C2+ | novel.5864 | 0.985047 | 0.986948 | HMG | 1 | 3 | |
| PH02Gene32056 | C1+C2+ | novel.5960 | 0.937659 | 0.951113 | HMG | 1 | 3 | |
| PH02Gene32056 | C1+C2+ | novel.7284 | 0.967089 | 0.936374 | HMG | 1 | 3 | |
| PH02Gene32056 | C1+C2+ | novel.8108 | 0.976694 | 0.948079 | HMG | 1 | 3 | |
| PH02Gene32056 | C1+C2+ | novel.8239 | 0.946115 | 0.980997 | HMG | 1 | 3 | |
| PH02Gene32056 | C1+C2+ | novel.8246 | 0.97271 | 0.970104 | HMG | 1 | 3 | |
| PH02Gene32056 | C1+C2+ | novel.8363 | 0.978708 | 0.970114 | HMG | 1 | 3 | |
| PH02Gene32056 | C1+C2+ | novel.8414 | 0.998952 | 0.964295 | HMG | 1 | 3 | |
| PH02Gene32056 | C1+C2+ | novel.8418 | 0.965607 | 0.937372 | HMG | 1 | 3 | |
| PH02Gene32056 | C1+C2+ | novel.8844 | 0.964652 | 0.946641 | HMG | 1 | 3 | |
| PH02Gene32056 | C1+C2+ | novel.9118 | 0.9349 | 0.942958 | HMG | 1 | 3 | |
| PH02Gene32056 | C1+C2+ | novel.9494 | 0.954064 | 0.958222 | HMG | 1 | 3 | |
| PH02Gene32056 | C1+C2+ | novel.9743 | 0.984002 | 0.951065 | HMG | 1 | 3 | |
| PH02Gene42615 | C1+C2+ | PH02Gene00004 | 0.995596 | 0.962663 | C2C2->C2C2-YABBY | 4 | 3 | |
| PH02Gene42615 | C1+C2+ | PH02Gene00409 | 0.966021 | 0.965599 | C2C2->C2C2-YABBY | 4 | 3 | |
| PH02Gene42615 | C1+C2+ | PH02Gene00481 | 0.99014 | 0.9609 | C2C2->C2C2-YABBY | 4 | 3 | |
| PH02Gene42615 | C1+C2+ | PH02Gene00677 | 0.98639 | 0.964526 | C2C2->C2C2-YABBY | 4 | 3 | |
| PH02Gene42615 | C1+C2+ | PH02Gene00750 | 0.941577 | 0.946042 | C2C2->C2C2-YABBY | 4 | 3 | |
| PH02Gene42615 | C1+C2+ | PH02Gene01182 | 0.933345 | 0.978214 | C2C2->C2C2-YABBY | 4 | 3 | |
| PH02Gene42615 | C1+C2+ | PH02Gene04480 | 0.979996 | 0.9882 | C2C2->C2C2-YABBY | 4 | 3 | |
| PH02Gene42615 | C1+C2+ | PH02Gene09246 | 0.961991 | 0.965723 | C2C2->C2C2-YABBY | 4 | 3 | |
| PH02Gene42615 | C1+C2+ | PH02Gene09449 | 0.938168 | 0.945147 | C2C2->C2C2-YABBY | 4 | 3 | |
| PH02Gene42615 | C1+C2+ | PH02Gene09952 | 0.938293 | 0.978755 | C2C2->C2C2-YABBY | 4 | 3 | |
| PH02Gene42615 | C1+C2+ | PH02Gene13613 | 0.986344 | 0.981992 | C2C2->C2C2-YABBY | 4 | 3 | |
| PH02Gene42615 | C1+C2+ | PH02Gene23475 | 0.992866 | 0.940205 | C2C2->C2C2-YABBY | 4 | 3 | |
| PH02Gene42615 | C1+C2+ | PH02Gene27104 | 0.983727 | 0.981205 | C2C2->C2C2-YABBY | 4 | 3 | |
| PH02Gene42615 | C1+C2+ | PH02Gene27740 | 0.963557 | 0.933175 | C2C2->C2C2-YABBY | 4 | 3 | |
| PH02Gene42615 | C1+C2+ | PH02Gene29089 | 0.982791 | 0.963863 | C2C2->C2C2-YABBY | 4 | 3 | |
| PH02Gene42615 | C1+C2+ | PH02Gene36157 | 0.932128 | 0.981095 | C2C2->C2C2-YABBY | 4 | 3 | |
| PH02Gene42615 | C1+C2+ | PH02Gene43454 | 0.977277 | 0.971074 | C2C2->C2C2-YABBY | 4 | 3 | |
| PH02Gene42615 | C1+C2+ | PH02Gene43507 | 0.9769 | 0.963619 | C2C2->C2C2-YABBY | 4 | 3 | |
| PH02Gene42615 | C1+C2+ | novel.14438 | 0.976507 | 0.954455 | C2C2->C2C2-YABBY | 4 | 3 | |
| PH02Gene42615 | C1+C2+ | novel.3672 | 0.994561 | 0.970815 | C2C2->C2C2-YABBY | 4 | 3 | |
| PH02Gene42615 | C1+C2+ | novel.7248 | 0.932114 | 0.941412 | C2C2->C2C2-YABBY | 4 | 3 | |
| PH02Gene42615 | C1+C2+ | novel.8679 | 0.977819 | 0.966092 | C2C2->C2C2-YABBY | 4 | 3 | |
| PH02Gene22915 | C1+C2+ | PH02Gene01046 | 0.968847 | 0.980014 | MYB->MYB | 2 | 3 | |
| PH02Gene22915 | C1+C2+ | PH02Gene02245 | 0.964653 | 0.952205 | MYB->MYB | 2 | 3 | |
| PH02Gene22915 | C1+C2+ | PH02Gene02370 | 0.96587 | 0.974682 | MYB->MYB | 2 | 3 | |
| PH02Gene22915 | C1+C2+ | PH02Gene03122 | 0.949776 | 0.968851 | MYB->MYB | 2 | 3 | |
| PH02Gene22915 | C1+C2+ | PH02Gene03773 | 0.95981 | 0.978023 | MYB->MYB | 2 | 3 | |
| PH02Gene22915 | C1+C2+ | PH02Gene03933 | 0.968093 | 0.982541 | MYB->MYB | 2 | 3 | |
| PH02Gene22915 | C1+C2+ | PH02Gene04045 | 0.970277 | 0.94217 | MYB->MYB | 2 | 3 | |
| PH02Gene22915 | C1+C2+ | PH02Gene05137 | 0.941543 | 0.933015 | MYB->MYB | 2 | 3 | |
| PH02Gene22915 | C1+C2+ | PH02Gene05858 | 0.987464 | 0.98123 | MYB->MYB | 2 | 3 | |
| PH02Gene22915 | C1+C2+ | PH02Gene06112 | 0.9707 | 0.93819 | MYB->MYB | 2 | 3 | |
| PH02Gene22915 | C1+C2+ | PH02Gene06296 | 0.950063 | 0.974364 | MYB->MYB | 2 | 3 | |
| PH02Gene22915 | C1+C2+ | PH02Gene07046 | 0.964502 | 0.944886 | MYB->MYB | 2 | 3 | |
| PH02Gene22915 | C1+C2+ | PH02Gene07136 | 0.958389 | 0.932799 | MYB->MYB | 2 | 4 | |
| PH02Gene22915 | C1+C2+ | PH02Gene07799 | 0.932907 | 0.95591 | MYB->MYB | 2 | 4 | |
| PH02Gene22915 | C1+C2+ | PH02Gene08241 | 0.966802 | 0.936559 | MYB->MYB | 2 | 4 | |
| PH02Gene22915 | C1+C2+ | PH02Gene08481 | 0.944378 | 0.952771 | MYB->MYB | 2 | 4 | |
| PH02Gene22915 | C1+C2+ | PH02Gene09040 | 0.957373 | 0.959959 | MYB->MYB | 2 | 4 | |
| PH02Gene22915 | C1+C2+ | PH02Gene10255 | 0.936364 | 0.979685 | MYB->MYB | 2 | 4 | |
| PH02Gene22915 | C1+C2+ | PH02Gene10408 | 0.954108 | 0.974008 | MYB->MYB | 2 | 4 | |
| PH02Gene22915 | C1+C2+ | PH02Gene10613 | 0.988716 | 0.932894 | MYB->MYB | 2 | 4 | |
| PH02Gene22915 | C1+C2+ | PH02Gene11956 | 0.942212 | 0.986023 | MYB->MYB | 2 | 4 | |
| PH02Gene22915 | C1+C2+ | PH02Gene12249 | 0.972074 | 0.98127 | MYB->MYB | 2 | 4 | |
| PH02Gene22915 | C1+C2+ | PH02Gene12450 | 0.968291 | 0.942432 | MYB->MYB | 2 | 4 | |
| PH02Gene22915 | C1+C2+ | PH02Gene13354 | 0.962206 | 0.975822 | MYB->MYB | 2 | 4 | |
| PH02Gene22915 | C1+C2+ | PH02Gene13621 | 0.979894 | 0.936663 | MYB->MYB | 2 | 4 | |
| PH02Gene22915 | C1+C2+ | PH02Gene15071 | 0.943133 | 0.968472 | MYB->MYB | 2 | 4 | |
| PH02Gene22915 | C1+C2+ | PH02Gene15773 | 0.934465 | 0.992897 | MYB->MYB | 2 | 4 | |
| PH02Gene22915 | C1+C2+ | PH02Gene16957 | 0.936045 | 0.965514 | MYB->MYB | 2 | 4 | |
| PH02Gene22915 | C1+C2+ | PH02Gene18038 | 0.938533 | 0.994526 | MYB->MYB | 2 | 4 | |
| PH02Gene22915 | C1+C2+ | PH02Gene18540 | 0.963608 | 0.997001 | MYB->MYB | 2 | 4 | |
| PH02Gene22915 | C1+C2+ | PH02Gene18625 | 0.930099 | 0.964476 | MYB->MYB | 2 | 4 | |
| PH02Gene22915 | C1+C2+ | PH02Gene19173 | 0.93588 | 0.996435 | MYB->MYB | 2 | 4 | |
| PH02Gene22915 | C1+C2+ | PH02Gene21297 | 0.984889 | 0.972242 | MYB->MYB | 2 | 4 | |
| PH02Gene22915 | C1+C2+ | PH02Gene21726 | 0.990219 | 0.941819 | MYB->MYB | 2 | 4 | |
| PH02Gene22915 | C1+C2+ | PH02Gene23033 | 0.963082 | 0.934058 | MYB->MYB | 2 | 4 | |
| PH02Gene22915 | C1+C2+ | PH02Gene23424 | 0.946303 | 0.996516 | MYB->MYB | 2 | 4 | |
| PH02Gene22915 | C1+C2+ | PH02Gene23653 | 0.963359 | 0.94012 | MYB->MYB | 2 | 4 | |
| PH02Gene22915 | C1+C2+ | PH02Gene23931 | 0.938132 | 0.974454 | MYB->MYB | 2 | 4 | |
| PH02Gene22915 | C1+C2+ | PH02Gene24363 | 0.992423 | 0.969181 | MYB->MYB | 2 | 4 | |
| PH02Gene22915 | C1+C2+ | PH02Gene26568 | 0.968546 | 0.973267 | MYB->MYB | 2 | 4 | |
| PH02Gene22915 | C1+C2+ | PH02Gene26736 | 0.951772 | 0.972908 | MYB->MYB | 2 | 4 | |
| PH02Gene22915 | C1+C2+ | PH02Gene28264 | 0.973063 | 0.972165 | MYB->MYB | 2 | 4 | |
| PH02Gene22915 | C1+C2+ | PH02Gene29628 | 0.947233 | 0.939777 | MYB->MYB | 2 | 4 | |
| PH02Gene22915 | C1+C2+ | PH02Gene30261 | 0.940696 | 0.986599 | MYB->MYB | 2 | 4 | |
| PH02Gene22915 | C1+C2+ | PH02Gene30475 | 0.983782 | 0.948375 | MYB->MYB | 2 | 4 | |
| PH02Gene22915 | C1+C2+ | PH02Gene31157 | 0.957747 | 0.95487 | MYB->MYB | 2 | 4 | |
| PH02Gene22915 | C1+C2+ | PH02Gene32068 | 0.988159 | 0.935385 | MYB->MYB | 2 | 4 | |
| PH02Gene22915 | C1+C2+ | PH02Gene32984 | 0.960578 | 0.972902 | MYB->MYB | 2 | 4 | |
| PH02Gene22915 | C1+C2+ | PH02Gene33375 | 0.968821 | 0.933304 | MYB->MYB | 2 | 4 | |
| PH02Gene22915 | C1+C2+ | PH02Gene33678 | 0.958856 | 0.954643 | MYB->MYB | 2 | 4 | |
| PH02Gene22915 | C1+C2+ | PH02Gene34308 | 0.973361 | 0.991996 | MYB->MYB | 2 | 4 | |
| PH02Gene22915 | C1+C2+ | PH02Gene34514 | 0.976066 | 0.979376 | MYB->MYB | 2 | 4 | |
| PH02Gene22915 | C1+C2+ | PH02Gene35012 | 0.985477 | 0.933917 | MYB->MYB | 2 | 4 | |
| PH02Gene22915 | C1+C2+ | PH02Gene35144 | 0.948987 | 0.957434 | MYB->MYB | 2 | 4 | |
| PH02Gene22915 | C1+C2+ | PH02Gene36486 | 0.977944 | 0.983457 | MYB->MYB | 2 | 4 | |
| PH02Gene22915 | C1+C2+ | PH02Gene36503 | 0.998161 | 0.931089 | MYB->MYB | 2 | 4 | |
| PH02Gene22915 | C1+C2+ | PH02Gene36574 | 0.958861 | 0.964682 | MYB->MYB | 2 | 4 | |
| PH02Gene22915 | C1+C2+ | PH02Gene36747 | 0.943728 | 0.98459 | MYB->MYB | 2 | 4 | |
| PH02Gene22915 | C1+C2+ | PH02Gene37257 | 0.945189 | 0.946308 | MYB->MYB | 2 | 4 | |
| PH02Gene22915 | C1+C2+ | PH02Gene38381 | 0.941697 | 0.935217 | MYB->MYB | 2 | 4 | |
| PH02Gene22915 | C1+C2+ | PH02Gene39628 | 0.967144 | 0.957882 | MYB->MYB | 2 | 4 | |
| PH02Gene22915 | C1+C2+ | PH02Gene39927 | 0.940327 | 0.954928 | MYB->MYB | 2 | 4 | |
| PH02Gene22915 | C1+C2+ | PH02Gene40120 | 0.968249 | 0.975778 | MYB->MYB | 2 | 4 | |
| PH02Gene22915 | C1+C2+ | PH02Gene40184 | 0.962159 | 0.947264 | MYB->MYB | 2 | 4 | |
| PH02Gene22915 | C1+C2+ | PH02Gene40941 | 0.976885 | 0.970639 | MYB->MYB | 2 | 4 | |
| PH02Gene22915 | C1+C2+ | PH02Gene41943 | 0.986612 | 0.958353 | MYB->MYB | 2 | 4 | |
| PH02Gene22915 | C1+C2+ | PH02Gene42066 | 0.959772 | 0.958045 | MYB->MYB | 2 | 4 | |
| PH02Gene22915 | C1+C2+ | PH02Gene43432 | 0.942998 | 0.953017 | MYB->MYB | 2 | 4 | |
| PH02Gene22915 | C1+C2+ | PH02Gene43508 | 0.953403 | 0.943464 | MYB->MYB | 2 | 4 | |
| PH02Gene22915 | C1+C2+ | PH02Gene43573 | 0.974376 | 0.930447 | MYB->MYB | 2 | 5 | |
| PH02Gene22915 | C1+C2+ | PH02Gene44046 | 0.97335 | 0.934317 | MYB->MYB | 2 | 5 | |
| PH02Gene22915 | C1+C2+ | PH02Gene45236 | 0.961852 | 0.961972 | MYB->MYB | 2 | 5 | |
| PH02Gene22915 | C1+C2+ | PH02Gene49708 | 0.950865 | 0.948193 | MYB->MYB | 2 | 5 | |
| PH02Gene22915 | C1+C2+ | PH02Gene50214 | 0.948029 | 0.966617 | MYB->MYB | 2 | 5 | |
| PH02Gene22915 | C1+C2+ | PH02Gene50597 | 0.990098 | 0.957319 | MYB->MYB | 2 | 5 | |
| PH02Gene22915 | C1+C2+ | novel.1038 | 0.955027 | 0.984819 | MYB->MYB | 2 | 5 | |
| PH02Gene22915 | C1+C2+ | novel.11331 | 0.943699 | 0.932975 | MYB->MYB | 2 | 5 | |
| PH02Gene22915 | C1+C2+ | novel.11797 | 0.961925 | 0.938421 | MYB->MYB | 2 | 5 | |
| PH02Gene22915 | C1+C2+ | novel.13734 | 0.999058 | 0.943892 | MYB->MYB | 2 | 5 | |
| PH02Gene22915 | C1+C2+ | novel.14142 | 0.964006 | 0.963236 | MYB->MYB | 2 | 5 | |
| PH02Gene22915 | C1+C2+ | novel.1836 | 0.946936 | 0.98785 | MYB->MYB | 2 | 5 | |
| PH02Gene22915 | C1+C2+ | novel.2234 | 0.93588 | 0.974589 | MYB->MYB | 2 | 5 | |
| PH02Gene22915 | C1+C2+ | novel.3446 | 0.956631 | 0.96148 | MYB->MYB | 2 | 5 | |
| PH02Gene22915 | C1+C2+ | novel.4162 | 0.982916 | 0.960943 | MYB->MYB | 2 | 5 | |
| PH02Gene22915 | C1+C2+ | novel.4842 | 0.972681 | 0.942302 | MYB->MYB | 2 | 5 | |
| PH02Gene22915 | C1+C2+ | novel.9468 | 0.932791 | 0.962296 | MYB->MYB | 2 | 5 | |
| PH02Gene27480 | C1+C2+ | PH02Gene07379 | 0.937331 | 0.961806 | MADS->MADS-MIKC | 4 | 5 | |
| PH02Gene27480 | C1+C2+ | PH02Gene10076 | 0.948595 | 0.980379 | MADS->MADS-MIKC | 4 | 5 | |
| PH02Gene27480 | C1+C2+ | PH02Gene11184 | 0.935081 | 0.999761 | MADS->MADS-MIKC | 4 | 5 | |
| PH02Gene27480 | C1+C2+ | PH02Gene15627 | 0.951703 | 0.963011 | MADS->MADS-MIKC | 4 | 5 | |
| PH02Gene27480 | C1+C2+ | PH02Gene22555 | 0.9417 | 0.991569 | MADS->MADS-MIKC | 4 | 5 | |
| PH02Gene27480 | C1+C2+ | PH02Gene29281 | 0.941452 | 0.958816 | MADS->MADS-MIKC | 4 | 5 | |
| PH02Gene27480 | C1+C2+ | PH02Gene31341 | 0.933965 | 0.970513 | MADS->MADS-MIKC | 4 | 5 | |
| PH02Gene27480 | C1+C2+ | PH02Gene36176 | 0.969778 | 0.959414 | MADS->MADS-MIKC | 4 | 5 | |
| PH02Gene27480 | C1+C2+ | PH02Gene39643 | 0.931751 | 0.944308 | MADS->MADS-MIKC | 4 | 5 | |
| PH02Gene27480 | C1+C2+ | PH02Gene40233 | 0.942318 | 0.962684 | MADS->MADS-MIKC | 4 | 5 | |
| PH02Gene27480 | C1+C2+ | PH02Gene43670 | 0.948068 | 0.986527 | MADS->MADS-MIKC | 4 | 5 | |
| PH02Gene27480 | C1+C2+ | PH02Gene47742 | 0.963938 | 0.990454 | MADS->MADS-MIKC | 4 | 5 | |
| PH02Gene27480 | C1+C2+ | PH02Gene51127 | 0.931009 | 0.975979 | MADS->MADS-MIKC | 4 | 5 | |
| PH02Gene27480 | C1+C2+ | novel.513 | 0.953608 | 0.952913 | MADS->MADS-MIKC | 4 | 5 | |
| PH02Gene07980 | C1+C2+ | PH02Gene00150 | 0.966446 | 0.983699 | B3->B3-ARF | 2 | 5 | |
| PH02Gene07980 | C1+C2+ | PH02Gene00151 | 0.991404 | 0.978326 | B3->B3-ARF | 2 | 5 | |
| PH02Gene07980 | C1+C2+ | PH02Gene00536 | 0.988513 | 0.954626 | B3->B3-ARF | 2 | 5 | |
| PH02Gene07980 | C1+C2+ | PH02Gene00691 | 0.931067 | 0.956092 | B3->B3-ARF | 2 | 5 | |
| PH02Gene07980 | C1+C2+ | PH02Gene00801 | 0.994576 | 0.932177 | B3->B3-ARF | 2 | 5 | |
| PH02Gene07980 | C1+C2+ | PH02Gene00813 | 0.961023 | 0.979759 | B3->B3-ARF | 2 | 5 | |
| PH02Gene07980 | C1+C2+ | PH02Gene01004 | 0.964855 | 0.965402 | B3->B3-ARF | 2 | 5 | |
| PH02Gene07980 | C1+C2+ | PH02Gene01086 | 0.949589 | 0.962661 | B3->B3-ARF | 2 | 5 | |
| PH02Gene07980 | C1+C2+ | PH02Gene01286 | 0.934718 | 0.943285 | B3->B3-ARF | 2 | 5 | |
| PH02Gene07980 | C1+C2+ | PH02Gene01458 | 0.968772 | 0.984424 | B3->B3-ARF | 2 | 5 | |
| PH02Gene07980 | C1+C2+ | PH02Gene01637 | 0.965433 | 0.959725 | B3->B3-ARF | 2 | 5 | |
| PH02Gene07980 | C1+C2+ | PH02Gene01814 | 0.937327 | 0.940264 | B3->B3-ARF | 2 | 5 | |
| PH02Gene07980 | C1+C2+ | PH02Gene02095 | 0.974475 | 0.991733 | B3->B3-ARF | 2 | 5 | |
| PH02Gene07980 | C1+C2+ | PH02Gene02162 | 0.947924 | 0.984044 | B3->B3-ARF | 2 | 5 | |
| PH02Gene07980 | C1+C2+ | PH02Gene02267 | 0.948264 | 0.988977 | B3->B3-ARF | 2 | 5 | |
| PH02Gene07980 | C1+C2+ | PH02Gene02272 | 0.983693 | 0.980156 | B3->B3-ARF | 2 | 6 | |
| PH02Gene07980 | C1+C2+ | PH02Gene02300 | 0.959923 | 0.961862 | B3->B3-ARF | 2 | 6 | |
| PH02Gene07980 | C1+C2+ | PH02Gene02352 | 0.962503 | 0.933362 | B3->B3-ARF | 2 | 6 | |
| PH02Gene07980 | C1+C2+ | PH02Gene02370 | 0.985913 | 0.955687 | B3->B3-ARF | 2 | 6 | |
| PH02Gene07980 | C1+C2+ | PH02Gene02429 | 0.984514 | 0.96845 | B3->B3-ARF | 2 | 6 | |
| PH02Gene07980 | C1+C2+ | PH02Gene02465 | 0.9312 | 0.957471 | B3->B3-ARF | 2 | 6 | |
| PH02Gene07980 | C1+C2+ | PH02Gene02542 | 0.954484 | 0.989321 | B3->B3-ARF | 2 | 6 | |
| PH02Gene07980 | C1+C2+ | PH02Gene03000 | 0.947473 | 0.942863 | B3->B3-ARF | 2 | 6 | |
| PH02Gene07980 | C1+C2+ | PH02Gene03085 | 0.947535 | 0.957551 | B3->B3-ARF | 2 | 6 | |
| PH02Gene07980 | C1+C2+ | PH02Gene03110 | 0.990935 | 0.975409 | B3->B3-ARF | 2 | 6 | |
| PH02Gene07980 | C1+C2+ | PH02Gene03122 | 0.952765 | 0.978789 | B3->B3-ARF | 2 | 6 | |
| PH02Gene07980 | C1+C2+ | PH02Gene03150 | 0.986684 | 0.931571 | B3->B3-ARF | 2 | 6 | |
| PH02Gene07980 | C1+C2+ | PH02Gene03369 | 0.958627 | 0.987009 | B3->B3-ARF | 2 | 6 | |
| PH02Gene07980 | C1+C2+ | PH02Gene03411 | 0.976873 | 0.951045 | B3->B3-ARF | 2 | 6 | |
| PH02Gene07980 | C1+C2+ | PH02Gene03773 | 0.962968 | 0.970937 | B3->B3-ARF | 2 | 6 | |
| PH02Gene07980 | C1+C2+ | PH02Gene03778 | 0.986135 | 0.981783 | B3->B3-ARF | 2 | 6 | |
| PH02Gene07980 | C1+C2+ | PH02Gene03782 | 0.984875 | 0.982237 | B3->B3-ARF | 2 | 6 | |
| PH02Gene07980 | C1+C2+ | PH02Gene03935 | 0.96351 | 0.978535 | B3->B3-ARF | 2 | 6 | |
| PH02Gene07980 | C1+C2+ | PH02Gene03946 | 0.968321 | 0.988541 | B3->B3-ARF | 2 | 6 | |
| PH02Gene07980 | C1+C2+ | PH02Gene03998 | 0.982346 | 0.967922 | B3->B3-ARF | 2 | 6 | |
| PH02Gene07980 | C1+C2+ | PH02Gene04026 | 0.989012 | 0.987145 | B3->B3-ARF | 2 | 6 | |
| PH02Gene07980 | C1+C2+ | PH02Gene04199 | 0.990145 | 0.967006 | B3->B3-ARF | 2 | 6 | |
| PH02Gene07980 | C1+C2+ | PH02Gene04400 | 0.932812 | 0.943956 | B3->B3-ARF | 2 | 6 | |
| PH02Gene07980 | C1+C2+ | PH02Gene04678 | 0.994221 | 0.958122 | B3->B3-ARF | 2 | 6 | |
| PH02Gene07980 | C1+C2+ | PH02Gene04862 | 0.945349 | 0.932258 | B3->B3-ARF | 2 | 7 | |
| PH02Gene07980 | C1+C2+ | PH02Gene04996 | 0.963623 | 0.939942 | B3->B3-ARF | 2 | 7 | |
| PH02Gene07980 | C1+C2+ | PH02Gene05134 | 0.973132 | 0.964672 | B3->B3-ARF | 2 | 7 | |
| PH02Gene07980 | C1+C2+ | PH02Gene05155 | 0.946387 | 0.98884 | B3->B3-ARF | 2 | 7 | |
| PH02Gene07980 | C1+C2+ | PH02Gene05439 | 0.956894 | 0.931541 | B3->B3-ARF | 2 | 7 | |
| PH02Gene07980 | C1+C2+ | PH02Gene05680 | 0.969545 | 0.988858 | B3->B3-ARF | 2 | 7 | |
| PH02Gene07980 | C1+C2+ | PH02Gene05856 | 0.983104 | 0.976328 | B3->B3-ARF | 2 | 7 | |
| PH02Gene07980 | C1+C2+ | PH02Gene05858 | 0.985712 | 0.937531 | B3->B3-ARF | 2 | 7 | |
| PH02Gene07980 | C1+C2+ | PH02Gene05885 | 0.989714 | 0.956587 | B3->B3-ARF | 2 | 7 | |
| PH02Gene07980 | C1+C2+ | PH02Gene06112 | 0.982455 | 0.936971 | B3->B3-ARF | 2 | 7 | |
| PH02Gene07980 | C1+C2+ | PH02Gene06288 | 0.98784 | 0.934744 | B3->B3-ARF | 2 | 7 | |
| PH02Gene07980 | C1+C2+ | PH02Gene06296 | 0.985266 | 0.953818 | B3->B3-ARF | 2 | 7 | |
| PH02Gene07980 | C1+C2+ | PH02Gene06399 | 0.98916 | 0.950083 | B3->B3-ARF | 2 | 7 | |
| PH02Gene07980 | C1+C2+ | PH02Gene06429 | 0.96927 | 0.977603 | B3->B3-ARF | 2 | 7 | |
| PH02Gene07980 | C1+C2+ | PH02Gene06550 | 0.970133 | 0.984044 | B3->B3-ARF | 2 | 7 | |
| PH02Gene07980 | C1+C2+ | PH02Gene06600 | 0.9795 | 0.972401 | B3->B3-ARF | 2 | 7 | |
| PH02Gene07980 | C1+C2+ | PH02Gene06704 | 0.962035 | 0.937179 | B3->B3-ARF | 2 | 7 | |
| PH02Gene07980 | C1+C2+ | PH02Gene07046 | 0.98045 | 0.955912 | B3->B3-ARF | 2 | 7 | |
| PH02Gene07980 | C1+C2+ | PH02Gene07287 | 0.963077 | 0.941686 | B3->B3-ARF | 2 | 7 | |
| PH02Gene07980 | C1+C2+ | PH02Gene07487 | 0.99471 | 0.968013 | B3->B3-ARF | 2 | 7 | |
| PH02Gene07980 | C1+C2+ | PH02Gene08100 | 0.958951 | 0.941939 | B3->B3-ARF | 2 | 7 | |
| PH02Gene07980 | C1+C2+ | PH02Gene08241 | 0.931627 | 0.957904 | B3->B3-ARF | 2 | 7 | |
| PH02Gene07980 | C1+C2+ | PH02Gene08767 | 0.984398 | 0.951261 | B3->B3-ARF | 2 | 7 | |
| PH02Gene07980 | C1+C2+ | PH02Gene09040 | 0.986013 | 0.934541 | B3->B3-ARF | 2 | 7 | |
| PH02Gene07980 | C1+C2+ | PH02Gene09228 | 0.982896 | 0.998559 | B3->B3-ARF | 2 | 7 | |
| PH02Gene07980 | C1+C2+ | PH02Gene09718 | 0.966171 | 0.940053 | B3->B3-ARF | 2 | 7 | |
| PH02Gene07980 | C1+C2+ | PH02Gene09809 | 0.931557 | 0.973432 | B3->B3-ARF | 2 | 7 | |
| PH02Gene07980 | C1+C2+ | PH02Gene09910 | 0.98362 | 0.991316 | B3->B3-ARF | 2 | 7 | |
| PH02Gene07980 | C1+C2+ | PH02Gene09927 | 0.965025 | 0.996695 | B3->B3-ARF | 2 | 7 | |
| PH02Gene07980 | C1+C2+ | PH02Gene10613 | 0.992361 | 0.945538 | B3->B3-ARF | 2 | 7 | |
| PH02Gene07980 | C1+C2+ | PH02Gene10822 | 0.965459 | 0.979127 | B3->B3-ARF | 2 | 7 | |
| PH02Gene07980 | C1+C2+ | PH02Gene10955 | 0.974509 | 0.951067 | B3->B3-ARF | 2 | 7 | |
| PH02Gene07980 | C1+C2+ | PH02Gene11377 | 0.940101 | 0.934824 | B3->B3-ARF | 2 | 7 | |
| PH02Gene07980 | C1+C2+ | PH02Gene11472 | 0.943682 | 0.95771 | B3->B3-ARF | 2 | 7 | |
| PH02Gene07980 | C1+C2+ | PH02Gene12177 | 0.975993 | 0.981377 | B3->B3-ARF | 2 | 7 | |
| PH02Gene07980 | C1+C2+ | PH02Gene12303 | 0.963505 | 0.996051 | B3->B3-ARF | 2 | 7 | |
| PH02Gene07980 | C1+C2+ | PH02Gene12430 | 0.972908 | 0.965256 | B3->B3-ARF | 2 | 7 | |
| PH02Gene07980 | C1+C2+ | PH02Gene12582 | 0.931699 | 0.981967 | B3->B3-ARF | 2 | 7 | |
| PH02Gene07980 | C1+C2+ | PH02Gene12711 | 0.963821 | 0.942926 | B3->B3-ARF | 2 | 7 | |
| PH02Gene07980 | C1+C2+ | PH02Gene12786 | 0.951857 | 0.955509 | B3->B3-ARF | 2 | 7 | |
| PH02Gene07980 | C1+C2+ | PH02Gene13033 | 0.978943 | 0.950406 | B3->B3-ARF | 2 | 7 | |
| PH02Gene07980 | C1+C2+ | PH02Gene13427 | 0.98866 | 0.982568 | B3->B3-ARF | 2 | 7 | |
| PH02Gene07980 | C1+C2+ | PH02Gene13568 | 0.970916 | 0.97547 | B3->B3-ARF | 2 | 7 | |
| PH02Gene07980 | C1+C2+ | PH02Gene13650 | 0.983236 | 0.958078 | B3->B3-ARF | 2 | 7 | |
| PH02Gene07980 | C1+C2+ | PH02Gene13701 | 0.949745 | 0.969657 | B3->B3-ARF | 2 | 7 | |
| PH02Gene07980 | C1+C2+ | PH02Gene13965 | 0.99477 | 0.984044 | B3->B3-ARF | 2 | 7 | |
| PH02Gene07980 | C1+C2+ | PH02Gene14032 | 0.972919 | 0.941352 | B3->B3-ARF | 2 | 7 | |
| PH02Gene07980 | C1+C2+ | PH02Gene14218 | 0.957074 | 0.938903 | B3->B3-ARF | 2 | 7 | |
| PH02Gene07980 | C1+C2+ | PH02Gene14386 | 0.984719 | 0.935234 | B3->B3-ARF | 2 | 7 | |
| PH02Gene07980 | C1+C2+ | PH02Gene14575 | 0.95331 | 0.980127 | B3->B3-ARF | 2 | 7 | |
| PH02Gene07980 | C1+C2+ | PH02Gene14584 | 0.946243 | 0.968514 | B3->B3-ARF | 2 | 7 | |
| PH02Gene07980 | C1+C2+ | PH02Gene14590 | 0.974338 | 0.978485 | B3->B3-ARF | 2 | 7 | |
| PH02Gene07980 | C1+C2+ | PH02Gene14608 | 0.955774 | 0.939132 | B3->B3-ARF | 2 | 7 | |
| PH02Gene07980 | C1+C2+ | PH02Gene14657 | 0.982245 | 0.98435 | B3->B3-ARF | 2 | 7 | |
| PH02Gene07980 | C1+C2+ | PH02Gene14662 | 0.984331 | 0.981969 | B3->B3-ARF | 2 | 7 | |
| PH02Gene07980 | C1+C2+ | PH02Gene14796 | 0.955756 | 0.997701 | B3->B3-ARF | 2 | 7 | |
| PH02Gene07980 | C1+C2+ | PH02Gene15071 | 0.985843 | 0.962187 | B3->B3-ARF | 2 | 7 | |
| PH02Gene07980 | C1+C2+ | PH02Gene15459 | 0.97375 | 0.987013 | B3->B3-ARF | 2 | 7 | |
| PH02Gene07980 | C1+C2+ | PH02Gene15685 | 0.944614 | 0.938801 | B3->B3-ARF | 2 | 7 | |
| PH02Gene07980 | C1+C2+ | PH02Gene15773 | 0.940977 | 0.937536 | B3->B3-ARF | 2 | 7 | |
| PH02Gene07980 | C1+C2+ | PH02Gene15812 | 0.969492 | 0.984666 | B3->B3-ARF | 2 | 7 | |
| PH02Gene07980 | C1+C2+ | PH02Gene16047 | 0.967098 | 0.968362 | B3->B3-ARF | 2 | 7 | |
| PH02Gene07980 | C1+C2+ | PH02Gene16160 | 0.941604 | 0.934649 | B3->B3-ARF | 2 | 7 | |
| PH02Gene07980 | C1+C2+ | PH02Gene16196 | 0.962072 | 0.933278 | B3->B3-ARF | 2 | 7 | |
| PH02Gene07980 | C1+C2+ | PH02Gene16344 | 0.979635 | 0.955625 | B3->B3-ARF | 2 | 7 | |
| PH02Gene07980 | C1+C2+ | PH02Gene17028 | 0.947775 | 0.948603 | B3->B3-ARF | 2 | 7 | |
| PH02Gene07980 | C1+C2+ | PH02Gene17539 | 0.953756 | 0.953575 | B3->B3-ARF | 2 | 7 | |
| PH02Gene07980 | C1+C2+ | PH02Gene17807 | 0.998125 | 0.978534 | B3->B3-ARF | 2 | 7 | |
| PH02Gene07980 | C1+C2+ | PH02Gene18038 | 0.958962 | 0.942729 | B3->B3-ARF | 2 | 7 | |
| PH02Gene07980 | C1+C2+ | PH02Gene18199 | 0.942775 | 0.946735 | B3->B3-ARF | 2 | 7 | |
| PH02Gene07980 | C1+C2+ | PH02Gene18276 | 0.980367 | 0.976128 | B3->B3-ARF | 2 | 7 | |
| PH02Gene07980 | C1+C2+ | PH02Gene18540 | 0.98931 | 0.935609 | B3->B3-ARF | 2 | 7 | |
| PH02Gene07980 | C1+C2+ | PH02Gene18663 | 0.940474 | 0.968438 | B3->B3-ARF | 2 | 7 | |
| PH02Gene07980 | C1+C2+ | PH02Gene18690 | 0.961746 | 0.9475 | B3->B3-ARF | 2 | 7 | |
| PH02Gene07980 | C1+C2+ | PH02Gene18737 | 0.954853 | 0.971706 | B3->B3-ARF | 2 | 7 | |
| PH02Gene07980 | C1+C2+ | PH02Gene18753 | 0.932545 | 0.956726 | B3->B3-ARF | 2 | 7 | |
| PH02Gene07980 | C1+C2+ | PH02Gene18756 | 0.964386 | 0.97207 | B3->B3-ARF | 2 | 7 | |
| PH02Gene07980 | C1+C2+ | PH02Gene18763 | 0.995068 | 0.988329 | B3->B3-ARF | 2 | 7 | |
| PH02Gene07980 | C1+C2+ | PH02Gene18944 | 0.97855 | 0.964231 | B3->B3-ARF | 2 | 7 | |
| PH02Gene07980 | C1+C2+ | PH02Gene19245 | 0.940978 | 0.969843 | B3->B3-ARF | 2 | 7 | |
| PH02Gene07980 | C1+C2+ | PH02Gene19544 | 0.986493 | 0.985738 | B3->B3-ARF | 2 | 7 | |
| PH02Gene07980 | C1+C2+ | PH02Gene19819 | 0.981514 | 0.971352 | B3->B3-ARF | 2 | 7 | |
| PH02Gene07980 | C1+C2+ | PH02Gene20031 | 0.984749 | 0.992519 | B3->B3-ARF | 2 | 7 | |
| PH02Gene07980 | C1+C2+ | PH02Gene20202 | 0.967008 | 0.961286 | B3->B3-ARF | 2 | 7 | |
| PH02Gene07980 | C1+C2+ | PH02Gene20920 | 0.932816 | 0.994637 | B3->B3-ARF | 2 | 7 | |
| PH02Gene07980 | C1+C2+ | PH02Gene20969 | 0.982395 | 0.987435 | B3->B3-ARF | 2 | 7 | |
| PH02Gene07980 | C1+C2+ | PH02Gene21590 | 0.979612 | 0.983632 | B3->B3-ARF | 2 | 7 | |
| PH02Gene07980 | C1+C2+ | PH02Gene21621 | 0.980371 | 0.985008 | B3->B3-ARF | 2 | 7 | |
| PH02Gene07980 | C1+C2+ | PH02Gene21744 | 0.963631 | 0.93491 | B3->B3-ARF | 2 | 7 | |
| PH02Gene07980 | C1+C2+ | PH02Gene21946 | 0.966106 | 0.948361 | B3->B3-ARF | 2 | 8 | |
| PH02Gene07980 | C1+C2+ | PH02Gene22265 | 0.988072 | 0.961952 | B3->B3-ARF | 2 | 8 | |
| PH02Gene07980 | C1+C2+ | PH02Gene22337 | 0.996011 | 0.954762 | B3->B3-ARF | 2 | 8 | |
| PH02Gene07980 | C1+C2+ | PH02Gene22734 | 0.980159 | 0.987242 | B3->B3-ARF | 2 | 8 | |
| PH02Gene07980 | C1+C2+ | PH02Gene23033 | 0.942823 | 0.970506 | B3->B3-ARF | 2 | 8 | |
| PH02Gene07980 | C1+C2+ | PH02Gene23069 | 0.993292 | 0.958569 | B3->B3-ARF | 2 | 8 | |
| PH02Gene07980 | C1+C2+ | PH02Gene23083 | 0.981353 | 0.977328 | B3->B3-ARF | 2 | 8 | |
| PH02Gene07980 | C1+C2+ | PH02Gene23391 | 0.981974 | 0.989526 | B3->B3-ARF | 2 | 8 | |
| PH02Gene07980 | C1+C2+ | PH02Gene23622 | 0.939781 | 0.948175 | B3->B3-ARF | 2 | 8 | |
| PH02Gene07980 | C1+C2+ | PH02Gene23898 | 0.960484 | 0.979997 | B3->B3-ARF | 2 | 8 | |
| PH02Gene07980 | C1+C2+ | PH02Gene24033 | 0.969417 | 0.961723 | B3->B3-ARF | 2 | 8 | |
| PH02Gene07980 | C1+C2+ | PH02Gene24034 | 0.990332 | 0.944811 | B3->B3-ARF | 2 | 8 | |
| PH02Gene07980 | C1+C2+ | PH02Gene24469 | 0.9325 | 0.984044 | B3->B3-ARF | 2 | 8 | |
| PH02Gene07980 | C1+C2+ | PH02Gene24633 | 0.990049 | 0.97358 | B3->B3-ARF | 2 | 8 | |
| PH02Gene07980 | C1+C2+ | PH02Gene25081 | 0.976581 | 0.968305 | B3->B3-ARF | 2 | 8 | |
| PH02Gene07980 | C1+C2+ | PH02Gene25208 | 0.969615 | 0.971858 | B3->B3-ARF | 2 | 8 | |
| PH02Gene07980 | C1+C2+ | PH02Gene25386 | 0.966558 | 0.986062 | B3->B3-ARF | 2 | 8 | |
| PH02Gene07980 | C1+C2+ | PH02Gene25441 | 0.963899 | 0.956716 | B3->B3-ARF | 2 | 8 | |
| PH02Gene07980 | C1+C2+ | PH02Gene25851 | 0.995975 | 0.985194 | B3->B3-ARF | 2 | 8 | |
| PH02Gene07980 | C1+C2+ | PH02Gene25880 | 0.97563 | 0.969777 | B3->B3-ARF | 2 | 8 | |
| PH02Gene07980 | C1+C2+ | PH02Gene25985 | 0.989284 | 0.95975 | B3->B3-ARF | 2 | 8 | |
| PH02Gene07980 | C1+C2+ | PH02Gene26502 | 0.977744 | 0.958517 | B3->B3-ARF | 2 | 8 | |
| PH02Gene07980 | C1+C2+ | PH02Gene26568 | 0.936801 | 0.965236 | B3->B3-ARF | 2 | 8 | |
| PH02Gene07980 | C1+C2+ | PH02Gene26736 | 0.992467 | 0.978694 | B3->B3-ARF | 2 | 8 | |
| PH02Gene07980 | C1+C2+ | PH02Gene26763 | 0.977776 | 0.984367 | B3->B3-ARF | 2 | 8 | |
| PH02Gene07980 | C1+C2+ | PH02Gene27008 | 0.982287 | 0.937946 | B3->B3-ARF | 2 | 8 | |
| PH02Gene07980 | C1+C2+ | PH02Gene27423 | 0.986057 | 0.964143 | B3->B3-ARF | 2 | 8 | |
| PH02Gene07980 | C1+C2+ | PH02Gene27948 | 0.981168 | 0.933576 | B3->B3-ARF | 2 | 8 | |
| PH02Gene07980 | C1+C2+ | PH02Gene28048 | 0.983142 | 0.964364 | B3->B3-ARF | 2 | 8 | |
| PH02Gene07980 | C1+C2+ | PH02Gene28184 | 0.970467 | 0.983063 | B3->B3-ARF | 2 | 8 | |
| PH02Gene07980 | C1+C2+ | PH02Gene28264 | 0.977717 | 0.957667 | B3->B3-ARF | 2 | 8 | |
| PH02Gene07980 | C1+C2+ | PH02Gene28291 | 0.967049 | 0.959938 | B3->B3-ARF | 2 | 8 | |
| PH02Gene07980 | C1+C2+ | PH02Gene28662 | 0.937995 | 0.959759 | B3->B3-ARF | 2 | 8 | |
| PH02Gene07980 | C1+C2+ | PH02Gene28681 | 0.958563 | 0.958426 | B3->B3-ARF | 2 | 8 | |
| PH02Gene07980 | C1+C2+ | PH02Gene28846 | 0.94732 | 0.951534 | B3->B3-ARF | 2 | 8 | |
| PH02Gene07980 | C1+C2+ | PH02Gene29032 | 0.980206 | 0.988668 | B3->B3-ARF | 2 | 8 | |
| PH02Gene07980 | C1+C2+ | PH02Gene29053 | 0.979423 | 0.939218 | B3->B3-ARF | 2 | 8 | |
| PH02Gene07980 | C1+C2+ | PH02Gene29090 | 0.977571 | 0.954119 | B3->B3-ARF | 2 | 8 | |
| PH02Gene07980 | C1+C2+ | PH02Gene29107 | 0.939271 | 0.966366 | B3->B3-ARF | 2 | 8 | |
| PH02Gene07980 | C1+C2+ | PH02Gene29181 | 0.956904 | 0.971297 | B3->B3-ARF | 2 | 8 | |
| PH02Gene07980 | C1+C2+ | PH02Gene29452 | 0.984055 | 0.986277 | B3->B3-ARF | 2 | 8 | |
| PH02Gene07980 | C1+C2+ | PH02Gene29871 | 0.962385 | 0.977327 | B3->B3-ARF | 2 | 8 | |
| PH02Gene07980 | C1+C2+ | PH02Gene29878 | 0.980917 | 0.995225 | B3->B3-ARF | 2 | 8 | |
| PH02Gene07980 | C1+C2+ | PH02Gene30825 | 0.982219 | 0.955593 | B3->B3-ARF | 2 | 8 | |
| PH02Gene07980 | C1+C2+ | PH02Gene31157 | 0.987857 | 0.97143 | B3->B3-ARF | 2 | 8 | |
| PH02Gene07980 | C1+C2+ | PH02Gene31224 | 0.958818 | 0.963287 | B3->B3-ARF | 2 | 8 | |
| PH02Gene07980 | C1+C2+ | PH02Gene31311 | 0.977048 | 0.984868 | B3->B3-ARF | 2 | 8 | |
| PH02Gene07980 | C1+C2+ | PH02Gene31572 | 0.958986 | 0.984044 | B3->B3-ARF | 2 | 8 | |
| PH02Gene07980 | C1+C2+ | PH02Gene31593 | 0.984309 | 0.978893 | B3->B3-ARF | 2 | 8 | |
| PH02Gene07980 | C1+C2+ | PH02Gene31722 | 0.970459 | 0.987668 | B3->B3-ARF | 2 | 8 | |
| PH02Gene07980 | C1+C2+ | PH02Gene31731 | 0.942956 | 0.955335 | B3->B3-ARF | 2 | 8 | |
| PH02Gene07980 | C1+C2+ | PH02Gene32056 | 0.965 | 0.973393 | B3->B3-ARF | 2 | 8 | |
| PH02Gene07980 | C1+C2+ | PH02Gene32253 | 0.930342 | 0.984136 | B3->B3-ARF | 2 | 8 | |
| PH02Gene07980 | C1+C2+ | PH02Gene32333 | 0.967069 | 0.985682 | B3->B3-ARF | 2 | 8 | |
| PH02Gene07980 | C1+C2+ | PH02Gene32394 | 0.98321 | 0.95658 | B3->B3-ARF | 2 | 8 | |
| PH02Gene07980 | C1+C2+ | PH02Gene32404 | 0.940351 | 0.968397 | B3->B3-ARF | 2 | 8 | |
| PH02Gene07980 | C1+C2+ | PH02Gene32582 | 0.981819 | 0.977396 | B3->B3-ARF | 2 | 8 | |
| PH02Gene07980 | C1+C2+ | PH02Gene32584 | 0.979427 | 0.954167 | B3->B3-ARF | 2 | 8 | |
| PH02Gene07980 | C1+C2+ | PH02Gene32984 | 0.991325 | 0.953664 | B3->B3-ARF | 2 | 8 | |
| PH02Gene07980 | C1+C2+ | PH02Gene33549 | 0.969743 | 0.936154 | B3->B3-ARF | 2 | 8 | |
| PH02Gene07980 | C1+C2+ | PH02Gene33723 | 0.98416 | 0.984044 | B3->B3-ARF | 2 | 8 | |
| PH02Gene07980 | C1+C2+ | PH02Gene33783 | 0.981335 | 0.962471 | B3->B3-ARF | 2 | 8 | |
| PH02Gene07980 | C1+C2+ | PH02Gene33785 | 0.937747 | 0.967631 | B3->B3-ARF | 2 | 8 | |
| PH02Gene07980 | C1+C2+ | PH02Gene34025 | 0.932113 | 0.984044 | B3->B3-ARF | 2 | 8 | |
| PH02Gene07980 | C1+C2+ | PH02Gene34062 | 0.984253 | 0.974638 | B3->B3-ARF | 2 | 8 | |
| PH02Gene07980 | C1+C2+ | PH02Gene34300 | 0.955606 | 0.984059 | B3->B3-ARF | 2 | 8 | |
| PH02Gene07980 | C1+C2+ | PH02Gene34422 | 0.954581 | 0.960419 | B3->B3-ARF | 2 | 8 | |
| PH02Gene07980 | C1+C2+ | PH02Gene34456 | 0.959332 | 0.984706 | B3->B3-ARF | 2 | 8 | |
| PH02Gene07980 | C1+C2+ | PH02Gene34477 | 0.948717 | 0.975113 | B3->B3-ARF | 2 | 8 | |
| PH02Gene07980 | C1+C2+ | PH02Gene34493 | 0.978533 | 0.951425 | B3->B3-ARF | 2 | 8 | |
| PH02Gene07980 | C1+C2+ | PH02Gene34514 | 0.995177 | 0.965319 | B3->B3-ARF | 2 | 8 | |
| PH02Gene07980 | C1+C2+ | PH02Gene34542 | 0.981624 | 0.96125 | B3->B3-ARF | 2 | 8 | |
| PH02Gene07980 | C1+C2+ | PH02Gene34676 | 0.968288 | 0.937346 | B3->B3-ARF | 2 | 8 | |
| PH02Gene07980 | C1+C2+ | PH02Gene35144 | 0.956371 | 0.961666 | B3->B3-ARF | 2 | 8 | |
| PH02Gene07980 | C1+C2+ | PH02Gene35175 | 0.983298 | 0.978112 | B3->B3-ARF | 2 | 8 | |
| PH02Gene07980 | C1+C2+ | PH02Gene35197 | 0.945227 | 0.990354 | B3->B3-ARF | 2 | 8 | |
| PH02Gene07980 | C1+C2+ | PH02Gene35265 | 0.962441 | 0.957352 | B3->B3-ARF | 2 | 8 | |
| PH02Gene07980 | C1+C2+ | PH02Gene35272 | 0.938315 | 0.933357 | B3->B3-ARF | 2 | 8 | |
| PH02Gene07980 | C1+C2+ | PH02Gene35461 | 0.98002 | 0.945678 | B3->B3-ARF | 2 | 8 | |
| PH02Gene07980 | C1+C2+ | PH02Gene36041 | 0.970428 | 0.936603 | B3->B3-ARF | 2 | 8 | |
| PH02Gene07980 | C1+C2+ | PH02Gene36349 | 0.930489 | 0.958448 | B3->B3-ARF | 2 | 8 | |
| PH02Gene07980 | C1+C2+ | PH02Gene36420 | 0.98265 | 0.9842 | B3->B3-ARF | 2 | 8 | |
| PH02Gene07980 | C1+C2+ | PH02Gene36503 | 0.976479 | 0.935759 | B3->B3-ARF | 2 | 8 | |
| PH02Gene07980 | C1+C2+ | PH02Gene36568 | 0.940131 | 0.984044 | B3->B3-ARF | 2 | 8 | |
| PH02Gene07980 | C1+C2+ | PH02Gene36574 | 0.957882 | 0.935812 | B3->B3-ARF | 2 | 8 | |
| PH02Gene07980 | C1+C2+ | PH02Gene36582 | 0.97938 | 0.981726 | B3->B3-ARF | 2 | 8 | |
| PH02Gene07980 | C1+C2+ | PH02Gene36608 | 0.95116 | 0.956281 | B3->B3-ARF | 2 | 8 | |
| PH02Gene07980 | C1+C2+ | PH02Gene36747 | 0.962189 | 0.941505 | B3->B3-ARF | 2 | 8 | |
| PH02Gene07980 | C1+C2+ | PH02Gene37018 | 0.979258 | 0.95679 | B3->B3-ARF | 2 | 8 | |
| PH02Gene07980 | C1+C2+ | PH02Gene37257 | 0.980509 | 0.94092 | B3->B3-ARF | 2 | 8 | |
| PH02Gene07980 | C1+C2+ | PH02Gene37334 | 0.980319 | 0.96054 | B3->B3-ARF | 2 | 8 | |
| PH02Gene07980 | C1+C2+ | PH02Gene37359 | 0.957262 | 0.983755 | B3->B3-ARF | 2 | 8 | |
| PH02Gene07980 | C1+C2+ | PH02Gene37503 | 0.944305 | 0.969873 | B3->B3-ARF | 2 | 8 | |
| PH02Gene07980 | C1+C2+ | PH02Gene37522 | 0.969457 | 0.977128 | B3->B3-ARF | 2 | 8 | |
| PH02Gene07980 | C1+C2+ | PH02Gene37635 | 0.985536 | 0.977888 | B3->B3-ARF | 2 | 8 | |
| PH02Gene07980 | C1+C2+ | PH02Gene37763 | 0.985627 | 0.984044 | B3->B3-ARF | 2 | 8 | |
| PH02Gene07980 | C1+C2+ | PH02Gene38367 | 0.982553 | 0.983269 | B3->B3-ARF | 2 | 8 | |
| PH02Gene07980 | C1+C2+ | PH02Gene38381 | 0.983575 | 0.980918 | B3->B3-ARF | 2 | 8 | |
| PH02Gene07980 | C1+C2+ | PH02Gene39220 | 0.987854 | 0.952071 | B3->B3-ARF | 2 | 8 | |
| PH02Gene07980 | C1+C2+ | PH02Gene39278 | 0.987829 | 0.942995 | B3->B3-ARF | 2 | 8 | |
| PH02Gene07980 | C1+C2+ | PH02Gene39281 | 0.986146 | 0.99769 | B3->B3-ARF | 2 | 8 | |
| PH02Gene07980 | C1+C2+ | PH02Gene39336 | 0.935005 | 0.932483 | B3->B3-ARF | 2 | 8 | |
| PH02Gene07980 | C1+C2+ | PH02Gene39509 | 0.991159 | 0.963156 | B3->B3-ARF | 2 | 8 | |
| PH02Gene07980 | C1+C2+ | PH02Gene39901 | 0.969937 | 0.953148 | B3->B3-ARF | 2 | 8 | |
| PH02Gene07980 | C1+C2+ | PH02Gene40016 | 0.966241 | 0.977903 | B3->B3-ARF | 2 | 8 | |
| PH02Gene07980 | C1+C2+ | PH02Gene40017 | 0.988466 | 0.968679 | B3->B3-ARF | 2 | 8 | |
| PH02Gene07980 | C1+C2+ | PH02Gene40040 | 0.971149 | 0.980248 | B3->B3-ARF | 2 | 8 | |
| PH02Gene07980 | C1+C2+ | PH02Gene40184 | 0.953137 | 0.944928 | B3->B3-ARF | 2 | 8 | |
| PH02Gene07980 | C1+C2+ | PH02Gene40223 | 0.981039 | 0.980059 | B3->B3-ARF | 2 | 8 | |
| PH02Gene07980 | C1+C2+ | PH02Gene40395 | 0.966421 | 0.954953 | B3->B3-ARF | 2 | 8 | |
| PH02Gene07980 | C1+C2+ | PH02Gene40419 | 0.94162 | 0.984044 | B3->B3-ARF | 2 | 8 | |
| PH02Gene07980 | C1+C2+ | PH02Gene40633 | 0.978063 | 0.93103 | B3->B3-ARF | 2 | 8 | |
| PH02Gene07980 | C1+C2+ | PH02Gene40926 | 0.99563 | 0.974002 | B3->B3-ARF | 2 | 8 | |
| PH02Gene07980 | C1+C2+ | PH02Gene40956 | 0.973548 | 0.965264 | B3->B3-ARF | 2 | 8 | |
| PH02Gene07980 | C1+C2+ | PH02Gene41327 | 0.983137 | 0.943213 | B3->B3-ARF | 2 | 8 | |
| PH02Gene07980 | C1+C2+ | PH02Gene41329 | 0.943863 | 0.981106 | B3->B3-ARF | 2 | 8 | |
| PH02Gene07980 | C1+C2+ | PH02Gene41404 | 0.977869 | 0.98548 | B3->B3-ARF | 2 | 8 | |
| PH02Gene07980 | C1+C2+ | PH02Gene41415 | 0.975673 | 0.984044 | B3->B3-ARF | 2 | 8 | |
| PH02Gene07980 | C1+C2+ | PH02Gene41647 | 0.931443 | 0.961683 | B3->B3-ARF | 2 | 8 | |
| PH02Gene07980 | C1+C2+ | PH02Gene41659 | 0.933245 | 0.934481 | B3->B3-ARF | 2 | 8 | |
| PH02Gene07980 | C1+C2+ | PH02Gene41784 | 0.994318 | 0.980463 | B3->B3-ARF | 2 | 8 | |
| PH02Gene07980 | C1+C2+ | PH02Gene41943 | 0.948506 | 0.951559 | B3->B3-ARF | 2 | 8 | |
| PH02Gene07980 | C1+C2+ | PH02Gene41951 | 0.98254 | 0.938964 | B3->B3-ARF | 2 | 8 | |
| PH02Gene07980 | C1+C2+ | PH02Gene42265 | 0.96747 | 0.965751 | B3->B3-ARF | 2 | 8 | |
| PH02Gene07980 | C1+C2+ | PH02Gene42272 | 0.990414 | 0.966217 | B3->B3-ARF | 2 | 8 | |
| PH02Gene07980 | C1+C2+ | PH02Gene42831 | 0.950151 | 0.984044 | B3->B3-ARF | 2 | 8 | |
| PH02Gene07980 | C1+C2+ | PH02Gene43005 | 0.988408 | 0.952851 | B3->B3-ARF | 2 | 8 | |
| PH02Gene07980 | C1+C2+ | PH02Gene43015 | 0.940778 | 0.983561 | B3->B3-ARF | 2 | 8 | |
| PH02Gene07980 | C1+C2+ | PH02Gene43148 | 0.943016 | 0.939721 | B3->B3-ARF | 2 | 8 | |
| PH02Gene07980 | C1+C2+ | PH02Gene43240 | 0.982523 | 0.933386 | B3->B3-ARF | 2 | 8 | |
| PH02Gene07980 | C1+C2+ | PH02Gene43432 | 0.983593 | 0.958185 | B3->B3-ARF | 2 | 8 | |
| PH02Gene07980 | C1+C2+ | PH02Gene43508 | 0.987065 | 0.976652 | B3->B3-ARF | 2 | 8 | |
| PH02Gene07980 | C1+C2+ | PH02Gene43540 | 0.979264 | 0.98422 | B3->B3-ARF | 2 | 8 | |
| PH02Gene07980 | C1+C2+ | PH02Gene43573 | 0.936331 | 0.94403 | B3->B3-ARF | 2 | 8 | |
| PH02Gene07980 | C1+C2+ | PH02Gene43612 | 0.952283 | 0.968954 | B3->B3-ARF | 2 | 8 | |
| PH02Gene07980 | C1+C2+ | PH02Gene44572 | 0.96297 | 0.942051 | B3->B3-ARF | 2 | 8 | |
| PH02Gene07980 | C1+C2+ | PH02Gene45075 | 0.982413 | 0.987744 | B3->B3-ARF | 2 | 8 | |
| PH02Gene07980 | C1+C2+ | PH02Gene45236 | 0.981736 | 0.970044 | B3->B3-ARF | 2 | 8 | |
| PH02Gene07980 | C1+C2+ | PH02Gene45459 | 0.998663 | 0.939119 | B3->B3-ARF | 2 | 8 | |
| PH02Gene07980 | C1+C2+ | PH02Gene45660 | 0.961442 | 0.995003 | B3->B3-ARF | 2 | 8 | |
| PH02Gene07980 | C1+C2+ | PH02Gene45733 | 0.971384 | 0.975156 | B3->B3-ARF | 2 | 8 | |
| PH02Gene07980 | C1+C2+ | PH02Gene45911 | 0.979878 | 0.942864 | B3->B3-ARF | 2 | 8 | |
| PH02Gene07980 | C1+C2+ | PH02Gene46407 | 0.946659 | 0.943182 | B3->B3-ARF | 2 | 8 | |
| PH02Gene07980 | C1+C2+ | PH02Gene46513 | 0.968721 | 0.983411 | B3->B3-ARF | 2 | 8 | |
| PH02Gene07980 | C1+C2+ | PH02Gene46889 | 0.941934 | 0.981861 | B3->B3-ARF | 2 | 8 | |
| PH02Gene07980 | C1+C2+ | PH02Gene47040 | 0.94338 | 0.97624 | B3->B3-ARF | 2 | 8 | |
| PH02Gene07980 | C1+C2+ | PH02Gene47168 | 0.973219 | 0.976802 | B3->B3-ARF | 2 | 8 | |
| PH02Gene07980 | C1+C2+ | PH02Gene47212 | 0.972615 | 0.984661 | B3->B3-ARF | 2 | 8 | |
| PH02Gene07980 | C1+C2+ | PH02Gene47485 | 0.986168 | 0.963298 | B3->B3-ARF | 2 | 8 | |
| PH02Gene07980 | C1+C2+ | PH02Gene48013 | 0.949045 | 0.933365 | B3->B3-ARF | 2 | 8 | |
| PH02Gene07980 | C1+C2+ | PH02Gene48194 | 0.990037 | 0.976391 | B3->B3-ARF | 2 | 8 | |
| PH02Gene07980 | C1+C2+ | PH02Gene48303 | 0.994956 | 0.93451 | B3->B3-ARF | 2 | 8 | |
| PH02Gene07980 | C1+C2+ | PH02Gene48654 | 0.936758 | 0.984044 | B3->B3-ARF | 2 | 8 | |
| PH02Gene07980 | C1+C2+ | PH02Gene48662 | 0.976535 | 0.968522 | B3->B3-ARF | 2 | 8 | |
| PH02Gene07980 | C1+C2+ | PH02Gene48689 | 0.961367 | 0.970469 | B3->B3-ARF | 2 | 8 | |
| PH02Gene07980 | C1+C2+ | PH02Gene48876 | 0.980057 | 0.984044 | B3->B3-ARF | 2 | 8 | |
| PH02Gene07980 | C1+C2+ | PH02Gene49111 | 0.941849 | 0.94765 | B3->B3-ARF | 2 | 8 | |
| PH02Gene07980 | C1+C2+ | PH02Gene49374 | 0.940251 | 0.956265 | B3->B3-ARF | 2 | 8 | |
| PH02Gene07980 | C1+C2+ | PH02Gene49708 | 0.984628 | 0.978278 | B3->B3-ARF | 2 | 8 | |
| PH02Gene07980 | C1+C2+ | PH02Gene50597 | 0.988021 | 0.970274 | B3->B3-ARF | 2 | 8 | |
| PH02Gene07980 | C1+C2+ | PH02Gene50823 | 0.97936 | 0.96893 | B3->B3-ARF | 2 | 8 | |
| PH02Gene07980 | C1+C2+ | PH02Gene50966 | 0.94439 | 0.999515 | B3->B3-ARF | 2 | 8 | |
| PH02Gene07980 | C1+C2+ | novel.10190 | 0.995851 | 0.950995 | B3->B3-ARF | 2 | 8 | |
| PH02Gene07980 | C1+C2+ | novel.1033 | 0.968678 | 0.978332 | B3->B3-ARF | 2 | 8 | |
| PH02Gene07980 | C1+C2+ | novel.10350 | 0.971482 | 0.948263 | B3->B3-ARF | 2 | 8 | |
| PH02Gene07980 | C1+C2+ | novel.10411 | 0.994382 | 0.974963 | B3->B3-ARF | 2 | 8 | |
| PH02Gene07980 | C1+C2+ | novel.10533 | 0.990454 | 0.984044 | B3->B3-ARF | 2 | 8 | |
| PH02Gene07980 | C1+C2+ | novel.12033 | 0.979531 | 0.984044 | B3->B3-ARF | 2 | 8 | |
| PH02Gene07980 | C1+C2+ | novel.12035 | 0.979884 | 0.98348 | B3->B3-ARF | 2 | 8 | |
| PH02Gene07980 | C1+C2+ | novel.12720 | 0.983949 | 0.964507 | B3->B3-ARF | 2 | 8 | |
| PH02Gene07980 | C1+C2+ | novel.12721 | 0.969387 | 0.96501 | B3->B3-ARF | 2 | 8 | |
| PH02Gene07980 | C1+C2+ | novel.12879 | 0.952536 | 0.958627 | B3->B3-ARF | 2 | 8 | |
| PH02Gene07980 | C1+C2+ | novel.13734 | 0.96341 | 0.98656 | B3->B3-ARF | 2 | 8 | |
| PH02Gene07980 | C1+C2+ | novel.13840 | 0.946716 | 0.973192 | B3->B3-ARF | 2 | 8 | |
| PH02Gene07980 | C1+C2+ | novel.14186 | 0.995817 | 0.954064 | B3->B3-ARF | 2 | 8 | |
| PH02Gene07980 | C1+C2+ | novel.14310 | 0.960676 | 0.958318 | B3->B3-ARF | 2 | 8 | |
| PH02Gene07980 | C1+C2+ | novel.14391 | 0.963707 | 0.999561 | B3->B3-ARF | 2 | 8 | |
| PH02Gene07980 | C1+C2+ | novel.14510 | 0.980534 | 0.969262 | B3->B3-ARF | 2 | 8 | |
| PH02Gene07980 | C1+C2+ | novel.14993 | 0.949196 | 0.961786 | B3->B3-ARF | 2 | 8 | |
| PH02Gene07980 | C1+C2+ | novel.15917 | 0.962881 | 0.947135 | B3->B3-ARF | 2 | 8 | |
| PH02Gene07980 | C1+C2+ | novel.2466 | 0.977646 | 0.992791 | B3->B3-ARF | 2 | 8 | |
| PH02Gene07980 | C1+C2+ | novel.2746 | 0.949774 | 0.951455 | B3->B3-ARF | 2 | 8 | |
| PH02Gene07980 | C1+C2+ | novel.3072 | 0.950434 | 0.956175 | B3->B3-ARF | 2 | 8 | |
| PH02Gene07980 | C1+C2+ | novel.3209 | 0.974494 | 0.97571 | B3->B3-ARF | 2 | 8 | |
| PH02Gene07980 | C1+C2+ | novel.3457 | 0.987444 | 0.936866 | B3->B3-ARF | 2 | 8 | |
| PH02Gene07980 | C1+C2+ | novel.3720 | 0.972792 | 0.936356 | B3->B3-ARF | 2 | 8 | |
| PH02Gene07980 | C1+C2+ | novel.3823 | 0.967136 | 0.997224 | B3->B3-ARF | 2 | 8 | |
| PH02Gene07980 | C1+C2+ | novel.3862 | 0.995364 | 0.966878 | B3->B3-ARF | 2 | 8 | |
| PH02Gene07980 | C1+C2+ | novel.4162 | 0.966593 | 0.975512 | B3->B3-ARF | 2 | 9 | |
| PH02Gene07980 | C1+C2+ | novel.4314 | 0.939756 | 0.937567 | B3->B3-ARF | 2 | 9 | |
| PH02Gene07980 | C1+C2+ | novel.4451 | 0.959549 | 0.970816 | B3->B3-ARF | 2 | 9 | |
| PH02Gene07980 | C1+C2+ | novel.4842 | 0.995703 | 0.981056 | B3->B3-ARF | 2 | 9 | |
| PH02Gene07980 | C1+C2+ | novel.490 | 0.939581 | 0.992541 | B3->B3-ARF | 2 | 9 | |
| PH02Gene07980 | C1+C2+ | novel.5503 | 0.994509 | 0.941175 | B3->B3-ARF | 2 | 9 | |
| PH02Gene07980 | C1+C2+ | novel.5864 | 0.932319 | 0.936408 | B3->B3-ARF | 2 | 9 | |
| PH02Gene07980 | C1+C2+ | novel.704 | 0.956194 | 0.968714 | B3->B3-ARF | 2 | 9 | |
| PH02Gene07980 | C1+C2+ | novel.8107 | 0.93751 | 0.994898 | B3->B3-ARF | 2 | 9 | |
| PH02Gene07980 | C1+C2+ | novel.8239 | 0.996676 | 0.982288 | B3->B3-ARF | 2 | 9 | |
| PH02Gene07980 | C1+C2+ | novel.8414 | 0.9658 | 0.957826 | B3->B3-ARF | 2 | 9 | |
| PH02Gene07980 | C1+C2+ | novel.8418 | 0.982745 | 0.975889 | B3->B3-ARF | 2 | 9 | |
| PH02Gene07980 | C1+C2+ | novel.9118 | 0.948884 | 0.953829 | B3->B3-ARF | 2 | 9 | |
| PH02Gene07980 | C1+C2+ | novel.9819 | 0.930195 | 0.977939 | B3->B3-ARF | 2 | 9 | |
| PH02Gene14941 | C1+C2+ | PH02Gene00107 | 0.95256 | 0.948044 | MYB->MYB | 5 | 9 | |
| PH02Gene14941 | C1+C2+ | PH02Gene04089 | 0.961527 | 0.993418 | MYB->MYB | 5 | 9 | |
| PH02Gene14941 | C1+C2+ | PH02Gene05225 | 0.968024 | 0.940044 | MYB->MYB | 5 | 9 | |
| PH02Gene14941 | C1+C2+ | PH02Gene06699 | 0.93496 | 0.938909 | MYB->MYB | 5 | 9 | |
| PH02Gene14941 | C1+C2+ | PH02Gene07585 | 0.962191 | 0.983056 | MYB->MYB | 5 | 9 | |
| PH02Gene14941 | C1+C2+ | PH02Gene11685 | 0.947385 | 0.95445 | MYB->MYB | 5 | 9 | |
| PH02Gene14941 | C1+C2+ | PH02Gene12216 | 0.980017 | 0.954219 | MYB->MYB | 5 | 9 | |
| PH02Gene14941 | C1+C2+ | PH02Gene26990 | 0.958346 | 0.94793 | MYB->MYB | 5 | 9 | |
| PH02Gene14941 | C1+C2+ | PH02Gene46019 | 0.935922 | 0.954208 | MYB->MYB | 5 | 9 | |
| PH02Gene14941 | C1+C2+ | novel.10052 | 0.954547 | 0.956808 | MYB->MYB | 5 | 9 | |
| PH02Gene14941 | C1+C2+ | novel.7951 | 0.934254 | 0.953176 | MYB->MYB | 5 | 9 | |
| PH02Gene14941 | C1+C2+ | novel.9303 | 0.930718 | 0.973291 | MYB->MYB | 5 | 9 | |
| PH02Gene06604 | C1+C2+ | PH02Gene00040 | 0.962566 | 0.953349 | MYB->MYB | 5 | 9 | |
| PH02Gene06604 | C1+C2+ | PH02Gene02264 | 0.936046 | 0.994685 | MYB->MYB | 5 | 9 | |
| PH02Gene27887 | C1+C2+ | PH02Gene02001 | 0.97416 | 0.94762 | C2C2->C2C2-Dof | 4 | 9 | |
| PH02Gene27887 | C1+C2+ | PH02Gene02471 | 0.96443 | 0.945398 | C2C2->C2C2-Dof | 4 | 9 | |
| PH02Gene27887 | C1+C2+ | PH02Gene03212 | 0.950444 | 0.96477 | C2C2->C2C2-Dof | 4 | 9 | |
| PH02Gene27887 | C1+C2+ | PH02Gene04896 | 0.961065 | 0.930891 | C2C2->C2C2-Dof | 4 | 9 | |
| PH02Gene27887 | C1+C2+ | PH02Gene06122 | 0.973632 | 0.96352 | C2C2->C2C2-Dof | 4 | 9 | |
| PH02Gene27887 | C1+C2+ | PH02Gene07703 | 0.935018 | 0.957986 | C2C2->C2C2-Dof | 4 | 9 | |
| PH02Gene27887 | C1+C2+ | PH02Gene08694 | 0.952667 | 0.955799 | C2C2->C2C2-Dof | 4 | 9 | |
| PH02Gene27887 | C1+C2+ | PH02Gene17756 | 0.933075 | 0.973154 | C2C2->C2C2-Dof | 4 | 9 | |
| PH02Gene27887 | C1+C2+ | PH02Gene22316 | 0.956804 | 0.980947 | C2C2->C2C2-Dof | 4 | 9 | |
| PH02Gene27887 | C1+C2+ | PH02Gene22798 | 0.955197 | 0.932762 | C2C2->C2C2-Dof | 4 | 9 | |
| PH02Gene27887 | C1+C2+ | PH02Gene25670 | 0.99358 | 0.935701 | C2C2->C2C2-Dof | 4 | 9 | |
| PH02Gene27887 | C1+C2+ | PH02Gene29011 | 0.964943 | 0.953518 | C2C2->C2C2-Dof | 4 | 9 | |
| PH02Gene27887 | C1+C2+ | PH02Gene30228 | 0.980109 | 0.938794 | C2C2->C2C2-Dof | 4 | 9 | |
| PH02Gene27887 | C1+C2+ | PH02Gene32762 | 0.952611 | 0.944983 | C2C2->C2C2-Dof | 4 | 9 | |
| PH02Gene27887 | C1+C2+ | PH02Gene33585 | 0.982574 | 0.941795 | C2C2->C2C2-Dof | 4 | 9 | |
| PH02Gene27887 | C1+C2+ | PH02Gene35053 | 0.963271 | 0.964211 | C2C2->C2C2-Dof | 4 | 9 | |
| PH02Gene27887 | C1+C2+ | novel.12398 | 0.958613 | 0.931851 | C2C2->C2C2-Dof | 4 | 9 | |
| PH02Gene27887 | C1+C2+ | novel.2743 | 0.933666 | 0.98475 | C2C2->C2C2-Dof | 4 | 9 | |
| PH02Gene27887 | C1+C2+ | novel.9372 | 0.971996 | 0.975024 | C2C2->C2C2-Dof | 4 | 9 | |
| PH02Gene16447 | C1+C2+ | PH02Gene17619 | 0.966507 | 0.9671 | bZIP | 6 | 9 | |
| PH02Gene16447 | C1+C2+ | PH02Gene18116 | 0.962865 | 0.971914 | bZIP | 6 | 9 | |
| PH02Gene16447 | C1+C2+ | PH02Gene25612 | 0.961939 | 0.974968 | bZIP | 6 | 9 | |
| PH02Gene16447 | C1+C2+ | PH02Gene28296 | 0.979802 | 0.967341 | bZIP | 6 | 9 | |
| PH02Gene16447 | C1+C2+ | PH02Gene37236 | 0.978888 | 0.940168 | bZIP | 6 | 9 | |
| PH02Gene16447 | C1+C2+ | novel.12207 | 0.965808 | 0.931959 | bZIP | 6 | 9 | |
| PH02Gene16447 | C1+C2+ | novel.4517 | 0.979747 | 0.939195 | bZIP | 6 | 9 | |
| PH02Gene03448 | C1+C2+ | PH02Gene00177 | 0.992017 | 0.968884 | bZIP | 6 | 9 | |
| PH02Gene03448 | C1+C2+ | PH02Gene00249 | 0.967042 | 0.977739 | bZIP | 6 | 9 | |
| PH02Gene03448 | C1+C2+ | PH02Gene00250 | 0.971937 | 0.964318 | bZIP | 6 | 9 | |
| PH02Gene03448 | C1+C2+ | PH02Gene00330 | 0.976546 | 0.952878 | bZIP | 6 | 9 | |
| PH02Gene03448 | C1+C2+ | PH02Gene00470 | 0.991826 | 0.974714 | bZIP | 6 | 9 | |
| PH02Gene03448 | C1+C2+ | PH02Gene00471 | 0.96901 | 0.953455 | bZIP | 6 | 9 | |
| PH02Gene03448 | C1+C2+ | PH02Gene00528 | 0.957428 | 0.947189 | bZIP | 6 | 9 | |
| PH02Gene03448 | C1+C2+ | PH02Gene00939 | 0.961772 | 0.938596 | bZIP | 6 | 9 | |
| PH02Gene03448 | C1+C2+ | PH02Gene01203 | 0.955768 | 0.941849 | bZIP | 6 | 9 | |
| PH02Gene03448 | C1+C2+ | PH02Gene01430 | 0.935768 | 0.989499 | bZIP | 6 | 9 | |
| PH02Gene03448 | C1+C2+ | PH02Gene01989 | 0.965128 | 0.977527 | bZIP | 6 | 9 | |
| PH02Gene03448 | C1+C2+ | PH02Gene02748 | 0.98699 | 0.958491 | bZIP | 6 | 9 | |
| PH02Gene03448 | C1+C2+ | PH02Gene02871 | 0.969042 | 0.966701 | bZIP | 6 | 9 | |
| PH02Gene03448 | C1+C2+ | PH02Gene03055 | 0.976995 | 0.986022 | bZIP | 6 | 9 | |
| PH02Gene03448 | C1+C2+ | PH02Gene03609 | 0.965908 | 0.957254 | bZIP | 6 | 9 | |
| PH02Gene03448 | C1+C2+ | PH02Gene04104 | 0.990133 | 0.996208 | bZIP | 6 | 9 | |
| PH02Gene03448 | C1+C2+ | PH02Gene04394 | 0.937379 | 0.991746 | bZIP | 6 | 9 | |
| PH02Gene03448 | C1+C2+ | PH02Gene04428 | 0.978232 | 0.994144 | bZIP | 6 | 9 | |
| PH02Gene03448 | C1+C2+ | PH02Gene04496 | 0.939708 | 0.935194 | bZIP | 6 | 9 | |
| PH02Gene03448 | C1+C2+ | PH02Gene04855 | 0.958641 | 0.963916 | bZIP | 6 | 9 | |
| PH02Gene03448 | C1+C2+ | PH02Gene05099 | 0.936609 | 0.969437 | bZIP | 6 | 9 | |
| PH02Gene03448 | C1+C2+ | PH02Gene05112 | 0.979458 | 0.949717 | bZIP | 6 | 9 | |
| PH02Gene03448 | C1+C2+ | PH02Gene05507 | 0.936974 | 0.965297 | bZIP | 6 | 9 | |
| PH02Gene03448 | C1+C2+ | PH02Gene06298 | 0.983954 | 0.958096 | bZIP | 6 | 9 | |
| PH02Gene03448 | C1+C2+ | PH02Gene06521 | 0.954614 | 0.945209 | bZIP | 6 | 9 | |
| PH02Gene03448 | C1+C2+ | PH02Gene06900 | 0.986656 | 0.953636 | bZIP | 6 | 9 | |
| PH02Gene03448 | C1+C2+ | PH02Gene07944 | 0.932177 | 0.962704 | bZIP | 6 | 9 | |
| PH02Gene03448 | C1+C2+ | PH02Gene08316 | 0.957721 | 0.948496 | bZIP | 6 | 9 | |
| PH02Gene03448 | C1+C2+ | PH02Gene08392 | 0.990927 | 0.98769 | bZIP | 6 | 9 | |
| PH02Gene03448 | C1+C2+ | PH02Gene08700 | 0.978195 | 0.931315 | bZIP | 6 | 9 | |
| PH02Gene03448 | C1+C2+ | PH02Gene08753 | 0.95463 | 0.956586 | bZIP | 6 | 9 | |
| PH02Gene03448 | C1+C2+ | PH02Gene08893 | 0.997899 | 0.945727 | bZIP | 6 | 9 | |
| PH02Gene03448 | C1+C2+ | PH02Gene08921 | 0.930884 | 0.941881 | bZIP | 6 | 9 | |
| PH02Gene03448 | C1+C2+ | PH02Gene09297 | 0.98192 | 0.97664 | bZIP | 6 | 9 | |
| PH02Gene03448 | C1+C2+ | PH02Gene09758 | 0.977369 | 0.940207 | bZIP | 6 | 9 | |
| PH02Gene03448 | C1+C2+ | PH02Gene09770 | 0.946083 | 0.94487 | bZIP | 6 | 9 | |
| PH02Gene03448 | C1+C2+ | PH02Gene09777 | 0.952768 | 0.947213 | bZIP | 6 | 9 | |
| PH02Gene03448 | C1+C2+ | PH02Gene10571 | 0.947048 | 0.959802 | bZIP | 6 | 9 | |
| PH02Gene03448 | C1+C2+ | PH02Gene10588 | 0.960292 | 0.9903 | bZIP | 6 | 9 | |
| PH02Gene03448 | C1+C2+ | PH02Gene11022 | 0.954778 | 0.9399 | bZIP | 6 | 9 | |
| PH02Gene03448 | C1+C2+ | PH02Gene11120 | 0.958937 | 0.987445 | bZIP | 6 | 9 | |
| PH02Gene03448 | C1+C2+ | PH02Gene11651 | 0.975584 | 0.95693 | bZIP | 6 | 9 | |
| PH02Gene03448 | C1+C2+ | PH02Gene11819 | 0.99608 | 0.958672 | bZIP | 6 | 10 | |
| PH02Gene03448 | C1+C2+ | PH02Gene11942 | 0.966996 | 0.940158 | bZIP | 6 | 10 | |
| PH02Gene03448 | C1+C2+ | PH02Gene12461 | 0.989819 | 0.951177 | bZIP | 6 | 10 | |
| PH02Gene03448 | C1+C2+ | PH02Gene12490 | 0.964891 | 0.962922 | bZIP | 6 | 10 | |
| PH02Gene03448 | C1+C2+ | PH02Gene13179 | 0.991463 | 0.968152 | bZIP | 6 | 10 | |
| PH02Gene03448 | C1+C2+ | PH02Gene13508 | 0.942905 | 0.954237 | bZIP | 6 | 10 | |
| PH02Gene03448 | C1+C2+ | PH02Gene13993 | 0.958256 | 0.960222 | bZIP | 6 | 10 | |
| PH02Gene03448 | C1+C2+ | PH02Gene14625 | 0.984449 | 0.987388 | bZIP | 6 | 10 | |
| PH02Gene03448 | C1+C2+ | PH02Gene14744 | 0.960118 | 0.958074 | bZIP | 6 | 10 | |
| PH02Gene03448 | C1+C2+ | PH02Gene14860 | 0.966191 | 0.952854 | bZIP | 6 | 10 | |
| PH02Gene03448 | C1+C2+ | PH02Gene15067 | 0.956585 | 0.988593 | bZIP | 6 | 10 | |
| PH02Gene03448 | C1+C2+ | PH02Gene15481 | 0.941175 | 0.944749 | bZIP | 6 | 10 | |
| PH02Gene03448 | C1+C2+ | PH02Gene15522 | 0.955363 | 0.992832 | bZIP | 6 | 10 | |
| PH02Gene03448 | C1+C2+ | PH02Gene15560 | 0.968128 | 0.96633 | bZIP | 6 | 10 | |
| PH02Gene03448 | C1+C2+ | PH02Gene15663 | 0.935251 | 0.947523 | bZIP | 6 | 10 | |
| PH02Gene03448 | C1+C2+ | PH02Gene15862 | 0.994845 | 0.97408 | bZIP | 6 | 10 | |
| PH02Gene03448 | C1+C2+ | PH02Gene15945 | 0.933757 | 0.994717 | bZIP | 6 | 10 | |
| PH02Gene03448 | C1+C2+ | PH02Gene16234 | 0.959134 | 0.951462 | bZIP | 6 | 10 | |
| PH02Gene03448 | C1+C2+ | PH02Gene16289 | 0.945482 | 0.963117 | bZIP | 6 | 10 | |
| PH02Gene03448 | C1+C2+ | PH02Gene17665 | 0.932329 | 0.97294 | bZIP | 6 | 10 | |
| PH02Gene03448 | C1+C2+ | PH02Gene17723 | 0.946114 | 0.952656 | bZIP | 6 | 10 | |
| PH02Gene03448 | C1+C2+ | PH02Gene17979 | 0.954447 | 0.969708 | bZIP | 6 | 10 | |
| PH02Gene03448 | C1+C2+ | PH02Gene18212 | 0.947384 | 0.969771 | bZIP | 6 | 10 | |
| PH02Gene03448 | C1+C2+ | PH02Gene18423 | 0.974718 | 0.935901 | bZIP | 6 | 10 | |
| PH02Gene03448 | C1+C2+ | PH02Gene18894 | 0.961032 | 0.984205 | bZIP | 6 | 10 | |
| PH02Gene03448 | C1+C2+ | PH02Gene19326 | 0.937679 | 0.976617 | bZIP | 6 | 10 | |
| PH02Gene03448 | C1+C2+ | PH02Gene19407 | 0.966269 | 0.986098 | bZIP | 6 | 10 | |
| PH02Gene03448 | C1+C2+ | PH02Gene19922 | 0.94302 | 0.937046 | bZIP | 6 | 10 | |
| PH02Gene03448 | C1+C2+ | PH02Gene19963 | 0.961528 | 0.968831 | bZIP | 6 | 10 | |
| PH02Gene03448 | C1+C2+ | PH02Gene20287 | 0.944239 | 0.942606 | bZIP | 6 | 10 | |
| PH02Gene03448 | C1+C2+ | PH02Gene20870 | 0.950901 | 0.979474 | bZIP | 6 | 10 | |
| PH02Gene03448 | C1+C2+ | PH02Gene20989 | 0.966958 | 0.955767 | bZIP | 6 | 10 | |
| PH02Gene03448 | C1+C2+ | PH02Gene21020 | 0.983025 | 0.970964 | bZIP | 6 | 10 | |
| PH02Gene03448 | C1+C2+ | PH02Gene21722 | 0.980534 | 0.941014 | bZIP | 6 | 10 | |
| PH02Gene03448 | C1+C2+ | PH02Gene21882 | 0.965231 | 0.975163 | bZIP | 6 | 10 | |
| PH02Gene03448 | C1+C2+ | PH02Gene22100 | 0.935442 | 0.971526 | bZIP | 6 | 10 | |
| PH02Gene03448 | C1+C2+ | PH02Gene23132 | 0.974531 | 0.997227 | bZIP | 6 | 10 | |
| PH02Gene03448 | C1+C2+ | PH02Gene23409 | 0.932735 | 0.937682 | bZIP | 6 | 10 | |
| PH02Gene03448 | C1+C2+ | PH02Gene23508 | 0.971846 | 0.96896 | bZIP | 6 | 10 | |
| PH02Gene03448 | C1+C2+ | PH02Gene23649 | 0.934132 | 0.956163 | bZIP | 6 | 10 | |
| PH02Gene03448 | C1+C2+ | PH02Gene23831 | 0.932091 | 0.960117 | bZIP | 6 | 10 | |
| PH02Gene03448 | C1+C2+ | PH02Gene24115 | 0.983158 | 0.950034 | bZIP | 6 | 10 | |
| PH02Gene03448 | C1+C2+ | PH02Gene24425 | 0.963759 | 0.99025 | bZIP | 6 | 10 | |
| PH02Gene03448 | C1+C2+ | PH02Gene24881 | 0.965894 | 0.935988 | bZIP | 6 | 10 | |
| PH02Gene03448 | C1+C2+ | PH02Gene25353 | 0.972409 | 0.958929 | bZIP | 6 | 10 | |
| PH02Gene03448 | C1+C2+ | PH02Gene26181 | 0.948011 | 0.995586 | bZIP | 6 | 10 | |
| PH02Gene03448 | C1+C2+ | PH02Gene26971 | 0.990229 | 0.982059 | bZIP | 6 | 10 | |
| PH02Gene03448 | C1+C2+ | PH02Gene27042 | 0.985096 | 0.965951 | bZIP | 6 | 10 | |
| PH02Gene03448 | C1+C2+ | PH02Gene27573 | 0.935193 | 0.942477 | bZIP | 6 | 10 | |
| PH02Gene03448 | C1+C2+ | PH02Gene27908 | 0.957209 | 0.972913 | bZIP | 6 | 10 | |
| PH02Gene03448 | C1+C2+ | PH02Gene28396 | 0.951198 | 0.97315 | bZIP | 6 | 10 | |
| PH02Gene03448 | C1+C2+ | PH02Gene28568 | 0.945733 | 0.949203 | bZIP | 6 | 10 | |
| PH02Gene03448 | C1+C2+ | PH02Gene28863 | 0.979903 | 0.94081 | bZIP | 6 | 10 | |
| PH02Gene03448 | C1+C2+ | PH02Gene29052 | 0.961404 | 0.962065 | bZIP | 6 | 10 | |
| PH02Gene03448 | C1+C2+ | PH02Gene29123 | 0.945698 | 0.955287 | bZIP | 6 | 10 | |
| PH02Gene03448 | C1+C2+ | PH02Gene29179 | 0.971306 | 0.93469 | bZIP | 6 | 10 | |
| PH02Gene03448 | C1+C2+ | PH02Gene29180 | 0.934857 | 0.980247 | bZIP | 6 | 10 | |
| PH02Gene03448 | C1+C2+ | PH02Gene30536 | 0.980475 | 0.981744 | bZIP | 6 | 10 | |
| PH02Gene03448 | C1+C2+ | PH02Gene30744 | 0.985825 | 0.967466 | bZIP | 6 | 10 | |
| PH02Gene03448 | C1+C2+ | PH02Gene30987 | 0.946873 | 0.94095 | bZIP | 6 | 10 | |
| PH02Gene03448 | C1+C2+ | PH02Gene31132 | 0.955181 | 0.985776 | bZIP | 6 | 10 | |
| PH02Gene03448 | C1+C2+ | PH02Gene31162 | 0.933667 | 0.971642 | bZIP | 6 | 10 | |
| PH02Gene03448 | C1+C2+ | PH02Gene31438 | 0.991213 | 0.945396 | bZIP | 6 | 10 | |
| PH02Gene03448 | C1+C2+ | PH02Gene31935 | 0.944215 | 0.94672 | bZIP | 6 | 10 | |
| PH02Gene03448 | C1+C2+ | PH02Gene32000 | 0.938021 | 0.979875 | bZIP | 6 | 10 | |
| PH02Gene03448 | C1+C2+ | PH02Gene32019 | 0.960019 | 0.979664 | bZIP | 6 | 10 | |
| PH02Gene03448 | C1+C2+ | PH02Gene32252 | 0.931774 | 0.939005 | bZIP | 6 | 10 | |
| PH02Gene03448 | C1+C2+ | PH02Gene32383 | 0.948659 | 0.940778 | bZIP | 6 | 10 | |
| PH02Gene03448 | C1+C2+ | PH02Gene32439 | 0.936314 | 0.971036 | bZIP | 6 | 10 | |
| PH02Gene03448 | C1+C2+ | PH02Gene32682 | 0.980253 | 0.952657 | bZIP | 6 | 10 | |
| PH02Gene03448 | C1+C2+ | PH02Gene33380 | 0.959556 | 0.960596 | bZIP | 6 | 10 | |
| PH02Gene03448 | C1+C2+ | PH02Gene34263 | 0.975007 | 0.941898 | bZIP | 6 | 10 | |
| PH02Gene03448 | C1+C2+ | PH02Gene34419 | 0.984656 | 0.949493 | bZIP | 6 | 10 | |
| PH02Gene03448 | C1+C2+ | PH02Gene34434 | 0.950989 | 0.94604 | bZIP | 6 | 10 | |
| PH02Gene03448 | C1+C2+ | PH02Gene35446 | 0.963793 | 0.97461 | bZIP | 6 | 10 | |
| PH02Gene03448 | C1+C2+ | PH02Gene35664 | 0.98962 | 0.990548 | bZIP | 6 | 10 | |
| PH02Gene03448 | C1+C2+ | PH02Gene36559 | 0.985714 | 0.983777 | bZIP | 6 | 10 | |
| PH02Gene03448 | C1+C2+ | PH02Gene38710 | 0.945425 | 0.945564 | bZIP | 6 | 10 | |
| PH02Gene03448 | C1+C2+ | PH02Gene38855 | 0.956618 | 0.954258 | bZIP | 6 | 10 | |
| PH02Gene03448 | C1+C2+ | PH02Gene38980 | 0.958678 | 0.944993 | bZIP | 6 | 10 | |
| PH02Gene03448 | C1+C2+ | PH02Gene39080 | 0.944895 | 0.992898 | bZIP | 6 | 10 | |
| PH02Gene03448 | C1+C2+ | PH02Gene39724 | 0.959668 | 0.966488 | bZIP | 6 | 10 | |
| PH02Gene03448 | C1+C2+ | PH02Gene39898 | 0.991337 | 0.950881 | bZIP | 6 | 10 | |
| PH02Gene03448 | C1+C2+ | PH02Gene39996 | 0.95493 | 0.946684 | bZIP | 6 | 10 | |
| PH02Gene03448 | C1+C2+ | PH02Gene40432 | 0.957792 | 0.9601 | bZIP | 6 | 10 | |
| PH02Gene03448 | C1+C2+ | PH02Gene40470 | 0.931086 | 0.977115 | bZIP | 6 | 10 | |
| PH02Gene03448 | C1+C2+ | PH02Gene41188 | 0.943775 | 0.972274 | bZIP | 6 | 10 | |
| PH02Gene03448 | C1+C2+ | PH02Gene41199 | 0.944448 | 0.930234 | bZIP | 6 | 10 | |
| PH02Gene03448 | C1+C2+ | PH02Gene41261 | 0.952836 | 0.98493 | bZIP | 6 | 10 | |
| PH02Gene03448 | C1+C2+ | PH02Gene41458 | 0.954137 | 0.948828 | bZIP | 6 | 10 | |
| PH02Gene03448 | C1+C2+ | PH02Gene41512 | 0.961019 | 0.985978 | bZIP | 6 | 10 | |
| PH02Gene03448 | C1+C2+ | PH02Gene42028 | 0.981435 | 0.968314 | bZIP | 6 | | 10 |
| PH02Gene03448 | C1+C2+ | PH02Gene42332 | 0.967262 | 0.935573 | bZIP | 6 | 10 | |
| PH02Gene03448 | C1+C2+ | PH02Gene42682 | 0.970205 | 0.960882 | bZIP | 6 | 10 | |
| PH02Gene03448 | C1+C2+ | PH02Gene44468 | 0.984093 | 0.98926 | bZIP | 6 | 10 | |
| PH02Gene03448 | C1+C2+ | PH02Gene44560 | 0.942127 | 0.983965 | bZIP | 6 | 10 | |
| PH02Gene03448 | C1+C2+ | PH02Gene44618 | 0.993473 | 0.983034 | bZIP | 6 | 10 | |
| PH02Gene03448 | C1+C2+ | PH02Gene44692 | 0.996384 | 0.9915 | bZIP | 6 | 10 | |
| PH02Gene03448 | C1+C2+ | PH02Gene44820 | 0.986882 | 0.973731 | bZIP | 6 | 10 | |
| PH02Gene03448 | C1+C2+ | PH02Gene45261 | 0.971145 | 0.951089 | bZIP | 6 | 10 | |
| PH02Gene03448 | C1+C2+ | PH02Gene45587 | 0.963674 | 0.948208 | bZIP | 6 | 10 | |
| PH02Gene03448 | C1+C2+ | PH02Gene46318 | 0.985748 | 0.97142 | bZIP | 6 | 10 | |
| PH02Gene03448 | C1+C2+ | PH02Gene46940 | 0.992229 | 0.944659 | bZIP | 6 | 10 | |
| PH02Gene03448 | C1+C2+ | PH02Gene48204 | 0.960362 | 0.965376 | bZIP | 6 | 10 | |
| PH02Gene03448 | C1+C2+ | PH02Gene48675 | 0.949192 | 0.936484 | bZIP | 6 | 10 | |
| PH02Gene03448 | C1+C2+ | PH02Gene49005 | 0.984643 | 0.944644 | bZIP | 6 | 10 | |
| PH02Gene03448 | C1+C2+ | PH02Gene49964 | 0.953443 | 0.978054 | bZIP | 6 | 10 | |
| PH02Gene03448 | C1+C2+ | PH02Gene50741 | 0.9705 | 0.960275 | bZIP | 6 | 10 | |
| PH02Gene03448 | C1+C2+ | PH02Gene51138 | 0.987444 | 0.973588 | bZIP | 6 | 10 | |
| PH02Gene03448 | C1+C2+ | novel.10334 | 0.958451 | 0.991884 | bZIP | 6 | 10 | |
| PH02Gene03448 | C1+C2+ | novel.10775 | 0.946462 | 0.992251 | bZIP | 6 | 10 | |
| PH02Gene03448 | C1+C2+ | novel.10788 | 0.981077 | 0.992171 | bZIP | 6 | 10 | |
| PH02Gene03448 | C1+C2+ | novel.11798 | 0.951757 | 0.966214 | bZIP | 6 | 10 | |
| PH02Gene03448 | C1+C2+ | novel.12153 | 0.98301 | 0.952849 | bZIP | 6 | 10 | |
| PH02Gene03448 | C1+C2+ | novel.13227 | 0.946982 | 0.943749 | bZIP | 6 | 10 | |
| PH02Gene03448 | C1+C2+ | novel.13649 | 0.973676 | 0.957311 | bZIP | 6 | 10 | |
| PH02Gene03448 | C1+C2+ | novel.14536 | 0.977832 | 0.934377 | bZIP | 6 | 10 | |
| PH02Gene03448 | C1+C2+ | novel.14601 | 0.961972 | 0.938141 | bZIP | 6 | 10 | |
| PH02Gene03448 | C1+C2+ | novel.15634 | 0.937657 | 0.972474 | bZIP | 6 | 10 | |
| PH02Gene03448 | C1+C2+ | novel.15807 | 0.973511 | 0.943355 | bZIP | 6 | 10 | |
| PH02Gene03448 | C1+C2+ | novel.2753 | 0.979995 | 0.948354 | bZIP | 6 | 10 | |
| PH02Gene03448 | C1+C2+ | novel.3002 | 0.992509 | 0.960716 | bZIP | 6 | 10 | |
| PH02Gene03448 | C1+C2+ | novel.391 | 0.970938 | 0.947022 | bZIP | 6 | 10 | |
| PH02Gene03448 | C1+C2+ | novel.4194 | 0.988501 | 0.974368 | bZIP | 6 | 10 | |
| PH02Gene03448 | C1+C2+ | novel.5423 | 0.985436 | 0.979385 | bZIP | 6 | 10 | |
| PH02Gene03448 | C1+C2+ | novel.5424 | 0.936433 | 0.988102 | bZIP | 6 | 10 | |
| PH02Gene03448 | C1+C2+ | novel.5967 | 0.975462 | 0.935295 | bZIP | 6 | 10 | |
| PH02Gene03448 | C1+C2+ | novel.6176 | 0.971496 | 0.958774 | bZIP | 6 | 10 | |
| PH02Gene03448 | C1+C2+ | novel.6691 | 0.943982 | 0.938415 | bZIP | 6 | 10 | |
| PH02Gene03448 | C1+C2+ | novel.6842 | 0.93006 | 0.976655 | bZIP | 6 | 10 | |
| PH02Gene03448 | C1+C2+ | novel.6964 | 0.935934 | 0.942583 | bZIP | 6 | 10 | |
| PH02Gene03448 | C1+C2+ | novel.7460 | 0.94149 | 0.965334 | bZIP | 6 | 10 | |
| PH02Gene03448 | C1+C2+ | novel.8848 | 0.952185 | 0.961307 | bZIP | 6 | 10 | |
| PH02Gene03448 | C1+C2+ | novel.8863 | 0.942478 | 0.961783 | bZIP | 6 | 10 | |
| PH02Gene03448 | C1+C2+ | novel.9824 | 0.950346 | 0.968488 | bZIP | 6 | 10 | |

C1 and C2 denote the *CmMYB4*-overexpression and the control plants, respectively. The “+” symbol indicates that the co-expressed gene pairs within the corresponding group show a positive correlation.

**Table S4.** Construction of the pigment biosynthesis subnetwork via BFS and PCC Filtering.

| **TF** | **Target** | **PCC1** | | **PCC2** | **Target Gene Type** | **TF Type** | **Target**  **Color** |
| --- | --- | --- | --- | --- | --- | --- | --- |
| C. morifolium 175740 | C. morifolium 56653 | 0.749365 | | 0.82162 |  | MYB |  |
| C. morifolium 175740 | C. morifolium 909161 | 0.847946 | | 0.952664 |  | MYB |  |
| C. morifolium 175740 | C. morifolium 1063670 | 0.402967 | | 0.429279 |  | MYB |  |
| C. morifolium 175740 | C. morifolium 11569117 | 0.467573 | | 0.568694 |  | MYB |  |
| C. morifolium 175740 | C. morifolium 113298 | 0.564702 | | 0.401976 |  | MYB |  |
| C. morifolium 175740 | C. morifolium 670288 | 0.43538 | | 0.9991 |  | MYB |  |
| C. morifolium 175740 | C. morifolium 1451103 | 0.481782 | | 0.446864 |  | MYB |  |
| C. morifolium 175740 | C. morifolium 878863 | 0.4036 | | 0.993883 |  | MYB |  |
| C. morifolium 175740 | C. morifolium 692109 | 0.719413 | | 0.801397 |  | MYB |  |
| C. morifolium 10190127 | C. morifolium asm7new1728 | 0.515837 | | 0.953867 |  | ERF |  |
| C. morifolium 10190127 | C. morifolium asm7new1726 | 0.660726 | | 0.933787 |  | ERF |  |
| C. morifolium 10190127 | C. morifolium 30301 | 0.472466 | | 0.816057 |  | ERF |  |
| C. morifolium 10190127 | C. morifolium 147018 | 0.583193 | | 0.56409 |  | ERF |  |
| C. morifolium 10190127 | C. morifolium 1697102 | 0.622868 | | 0.823813 |  | ERF |  |
| C. morifolium 10190127 | C. morifolium 361556 | 0.464184 | | 0.858309 |  | ERF |  |
| C. morifolium 10190127 | C. morifolium 3326180 | 0.524946 | | 0.94648 |  | ERF |  |
| C. morifolium 10190127 | C. morifolium 181219 | 0.553204 | | 0.963778 |  | ERF |  |
| C. morifolium 10190127 | C. morifolium 113298 | 0.806858 | | 0.821461 |  | ERF |  |
| C. morifolium 10190127 | C. morifolium 58291 | 0.435676 | | 0.77129 |  | ERF |  |
| C. morifolium 10190127 | C. morifolium 6893234 | 0.862539 | | 0.926939 |  | ERF |  |
| C. morifolium 10190127 | C. morifolium 75986 | 0.516569 | | 0.99106 |  | ERF |  |
| C. morifolium 10190127 | C. morifolium 182257 | 0.61213 | | 0.8107 |  | ERF |  |
| C. morifolium 10190127 | C. morifolium 28695 | 0.685857 | | 0.61589 |  | ERF |  |
| C. morifolium 10190127 | C. morifolium 1099236 | 0.626475 | | 0.666089 |  | ERF |  |
| C. morifolium 10190127 | C. morifolium 824957 | 0.426493 | | 0.763477 |  | ERF |  |
| C. morifolium 10190127 | C. morifolium 361788 | 0.696412 | | 0.684192 |  | ERF |  |
| C. morifolium 10190127 | C. morifolium 108296 | 0.489716 | | 0.890015 |  | ERF |  |
| C. morifolium 10190127 | C. morifolium 174265 | 0.81736 | | 0.961201 |  | ERF |  |
| C. morifolium 10190127 | C. morifolium 7419236 | 0.716408 | | 0.940759 |  | ERF |  |
| C. morifolium 10190127 | C. morifolium 106596 | 0.571308 | | 0.942312 |  | ERF |  |
| C. morifolium 10190127 | C. morifolium 422311 | 0.555961 | | 0.426854 |  | ERF |  |
| C. morifolium 10190127 | C. morifolium 78237 | 0.633742 | | 0.796529 |  | ERF |  |
| C. morifolium 10190127 | C. morifolium 1749435 | 0.673511 | | 0.982615 |  | ERF |  |
| C. morifolium 10190127 | C. morifolium 1749666 | 0.836611 | | 0.983919 |  | ERF |  |
| C. morifolium 10190127 | C. morifolium 770260 | 0.735478 | | 0.980133 |  | ERF |  |
| C. morifolium 10190127 | C. morifolium 764299 | 0.495845 | | 0.796938 |  | ERF |  |
| C. morifolium 10190127 | C. morifolium 1170588 | 0.717982 | | 0.488864 |  | ERF |  |
| C. morifolium 10190127 | C. morifolium 56646 | 0.667489 | | 0.970837 |  | ERF |  |
| C. morifolium 10190127 | C. morifolium 30186 | 0.585935 | | 0.925255 |  | ERF |  |
| C. morifolium 10190127 | C. morifolium 1474198 | 0.6715 | | 0.877146 |  | ERF |  |
| C. morifolium 10190127 | C. morifolium 10264300 | 0.759257 | | 0.442621 |  | ERF |  |
| C. morifolium 10190127 | C. morifolium 691689 | 0.784372 | | 0.961059 |  | ERF |  |
| C. morifolium 10190127 | C. morifolium 4568142 | 0.649623 | | 0.900303 |  | ERF |  |
| C. morifolium 10190127 | C. morifolium 598418 | 0.700163 | | 0.901121 |  | ERF |  |
| C. morifolium 10190127 | C. morifolium 808583 | 0.692953 | | 0.823801 |  | ERF |  |
| C. morifolium 10190127 | C. morifolium 1333588 | 0.73348 | | 0.713232 |  | ERF |  |
| C. morifolium 10190127 | C. morifolium 110403 | 0.559919 | | 0.932638 |  | ERF |  |
| C. morifolium 10190127 | C. morifolium 149253 | 0.770622 | | 0.968644 |  | ERF |  |
| C. morifolium 10190127 | C. morifolium 581227 | 0.709209 | | 0.984872 |  | ERF |  |
| C. morifolium 10190127 | C. morifolium 170964 | 0.500454 | | 0.946938 |  | ERF |  |
| C. morifolium 10190127 | C. morifolium 351636 | 0.935656 | | 0.641758 |  | ERF |  |
| C. morifolium 10190127 | C. morifolium 17652 | 0.785302 | | 0.526673 |  | ERF |  |
| C. morifolium 10190127 | C. morifolium 669838 | 0.785302 | | 0.526673 |  | ERF |  |
| C. morifolium 10190127 | C. morifolium 168245 | 0.698958 | | 0.521917 |  | ERF |  |
| C. morifolium 10190127 | C. morifolium 1248116 | 0.798654 | | 0.863129 |  | ERF |  |
| C. morifolium 10190127 | C. morifolium 249115 | 0.521713 | | 0.933884 |  | ERF |  |
| C. morifolium 10190127 | C. morifolium 1146479 | 0.631756 | | 0.95415 |  | ERF |  |
| C. morifolium 10190127 | C. morifolium 911187 | 0.511868 | | 0.871757 |  | ERF |  |
| C. morifolium 10190127 | C. morifolium 669837 | 0.746979 | | 0.665317 |  | ERF |  |
| C. morifolium 10190127 | C. morifolium 285338 | 0.726752 | | 0.790596 |  | ERF |  |
| C. morifolium 10190127 | C. morifolium 3601235 | 0.512512 | | 0.940124 |  | ERF |  |
| C. morifolium 10190127 | C. morifolium 798334 | 0.645814 | | 0.856324 |  | ERF |  |
| C. morifolium 10190127 | C. morifolium 994639 | 0.480187 | | 0.961879 |  | ERF |  |
| C. morifolium 10190127 | C. morifolium 105810 | 0.984171 | | 0.954814 |  | ERF |  |
| C. morifolium 10190127 | C. morifolium 1024382 | 0.523617 | | 0.887424 |  | ERF |  |
| C. morifolium 10190127 | C. morifolium 117390 | 0.462518 | | 0.435421 |  | ERF |  |
| C. morifolium 10190127 | C. morifolium 5424107 | 0.842221 | | 0.900374 |  | ERF |  |
| C. morifolium 10190127 | C. morifolium 4190216 | 0.651533 | | 0.584236 |  | ERF |  |
| C. morifolium 10190127 | C. morifolium 1146437 | 0.420092 | | 0.815549 |  | ERF |  |
| C. morifolium 10190127 | C. morifolium 1353 | 0.758787 | | 0.668653 |  | ERF |  |
| C. morifolium 10190127 | C. morifolium 1622283 | 0.42126 | | 0.892448 |  | ERF |  |
| C. morifolium 10190127 | C. morifolium 1806852 | 0.661512 | | 0.810716 |  | ERF |  |
| C. morifolium 10190127 | C. morifolium 1260337 | 0.697365 | | 0.560987 |  | ERF |  |
| C. morifolium 10190127 | C. morifolium 570336 | 0.672456 | | 0.829869 |  | ERF |  |
| C. morifolium 10190127 | C. morifolium 1659446 | 0.884461 | | 0.598708 |  | ERF |  |
| C. morifolium 10190127 | C. morifolium 10264116 | 0.734139 | | 0.918493 |  | ERF |  |
| C. morifolium 10190127 | C. morifolium 581199 | 0.707522 | | 0.955184 |  | ERF |  |
| C. morifolium 10190127 | C. morifolium 350101 | 0.451935 | | 0.48966 |  | ERF |  |
| C. morifolium 10190127 | C. morifolium 1660118 | 0.495532 | | 0.464859 |  | ERF |  |
| C. morifolium 10190127 | C. morifolium 40957 | 0.864361 | | 0.746517 |  | ERF |  |
| C. morifolium 10190127 | C. morifolium 141018 | 0.720946 | | 0.755976 |  | ERF |  |
| C. morifolium 10190127 | C. morifolium 33951 | 0.518892 | | 0.864263 |  | ERF |  |
| C. morifolium 10190127 | C. morifolium 1024380 | 0.735431 | | 0.78373 |  | ERF |  |
| C. morifolium 10190127 | C. morifolium 1026979 | 0.89507 | | 0.471931 |  | ERF |  |
| C. morifolium 10190127 | C. morifolium 925850 | 0.837806 | | 0.676178 |  | ERF |  |
| C. morifolium 10190127 | C. morifolium 1034690 | 0.483684 | | 0.616895 |  | ERF |  |
| C. morifolium 10190127 | C. morifolium 149246 | 0.587645 | | 0.781269 |  | ERF |  |
| C. morifolium 10190127 | C. morifolium 615892 | 0.542985 | | 0.942468 |  | ERF |  |
| C. morifolium 10190127 | C. morifolium 153724 | 0.740378 | | 0.743679 |  | ERF |  |
| C. morifolium 10190127 | C. morifolium 477584 | 0.715644 | | 0.648742 |  | ERF |  |
| C. morifolium 10190127 | C. morifolium 1749144 | 0.502346 | | 0.689327 |  | ERF |  |
| C. morifolium 10190127 | C. morifolium 330911 | 0.750392 | | 0.63309 |  | ERF |  |
| C. morifolium 10190127 | C. morifolium 25470 | 0.946944 | | 0.674601 |  | ERF |  |
| C. morifolium 10190127 | C. morifolium 670250 | 0.423635 | | 0.874299 |  | ERF |  |
| C. morifolium 10190127 | C. morifolium 25186 | 0.597953 | | 0.925866 |  | ERF |  |
| C. morifolium 10190127 | C. morifolium 95081 | 0.491119 | | 0.700338 |  | ERF |  |
| C. morifolium 10190127 | C. morifolium 279241 | 0.410942 | | 0.821662 |  | ERF |  |
| C. morifolium 10190127 | C. morifolium 2263 | 0.508756 | | 0.809768 |  | ERF |  |
| C. morifolium 10190127 | C. morifolium 9340601 | 0.592953 | | 0.598618 |  | ERF |  |
| C. morifolium 10190127 | C. morifolium 1156220 | 0.770002 | | 0.628562 |  | ERF |  |
| C. morifolium 56653 | C. morifolium 225621 PSY | 0.451069 | | 0.97301 | PSY | ERF | yellow |
| C. morifolium 909161 | C. morifolium 225621 PSY | 0.420306 | | 0.758135 | PSY | ARF | yellow |
| C. morifolium 1063670 | C. morifolium 839138 GSA1  (HEML1) | 0.512625 | | 0.467424 | GSA1  (HEML1) | HSF | green |
| C. morifolium 11569117 | C. morifolium 225621 PSY | 0.706984 | | 0.519352 | PSY | ERF | yellow |
| C. morifolium 11569117 | C. morifolium 140660 F3H | 0.652683 | | 0.511076 | F3H | ERF | purple |
| C. morifolium 11569117 | C. morifolium 14381001 F3H | 0.591018 | | 0.501982 | F3H | ERF | purple |
| C. morifolium 11569117 | C. morifolium 174438 CHS | 0.568646 | | 0.502355 | CHS | ERF | purple |
| C. morifolium 11569117 | C. morifolium 206 F3H | 0.638631 | | 0.492146 | F3H | ERF | purple |
| C. morifolium 11569117 | C. morifolium 36771 F3H | 0.647551 | | 0.487448 | F3H | ERF | purple |
| C. morifolium 11569117 | C. morifolium 38223 F3H | 0.692959 | | 0.512936 | F3H | ERF | purple |
| C. morifolium 11569117 | C. morifolium 46559 CHS | 0.500358 | | 0.489295 | CHS | ERF | purple |
| C. morifolium 11569117 | C. morifolium 934078 DFR | 0.624055 | | 0.520373 | DFR | ERF | purple |
| C. morifolium 11569117 | C. morifolium 1081892 3GT | 0.692987 | | 0.529797 | 3GT | ERF | purple |
| C. morifolium 11569117 | C. morifolium 201250 3GT | 0.620329 | | 0.51933 | 3GT | ERF | purple |
| C. morifolium 11569117 | C. morifolium 10628156 3MT | 0.847542 | | 0.514933 | 3MT | ERF | purple |
| C. morifolium 11569117 | C. morifolium 1266437 3MT | 0.832298 | | 0.609035 | 3MT | ERF | purple |
| C. morifolium 11569117 | C. morifolium 144657 3MT | 0.830551 | | 0.627593 | 3MT | ERF | purple |
| C. morifolium 11569117 | C. morifolium 37122 ANS2 | 0.787737 | | 0.515709 | ANS2 | ERF | purple |
| C. morifolium 113298 | C. morifolium 121984 PORA | 0.488904 | | 0.770628 | PORA | C2H2 | green |
| C. morifolium 113298 | C. morifolium 500315 CCD | | 0.413052 | 0.730638 | CCD | C2H2 | yellow |
| C. morifolium 670288 | C. morifolium 225621 PSY | 0.699932 | | 0.776161 | PSY | ERF | yellow |
| C. morifolium 1451103 | C. morifolium 839138 GSA1(HEML1) | 0.520861 | | 0.547105 | GSA1  (HEML1) | MYB | green |
| C. morifolium 1451103 | C. morifolium 1719125 GSA2(HEML2) | 0.73332 | | 0.77183 | GSA2  (HEML2) | MYB | green |
| C. morifolium 1451103 | C. morifolium 1659259 HEME2 | 0.702846 | | 0.423082 | HEME2 | MYB | green |
| C. morifolium 1451103 | C. morifolium 2224124 HEMF1 | 0.534389 | | 0.683059 | HEMF1 | MYB | green |
| C. morifolium 1451103 | C. morifolium 113088 F3'H | 0.851614 | | 0.554808 | F3'H | MYB | purple |
| C. morifolium 1451103 | C. morifolium 1139541 CHI | 0.853396 | | 0.560539 | CHI | MYB | purple |
| C. morifolium 1451103 | C. morifolium 114633 CHS | 0.853847 | | 0.578663 | CHS | MYB | purple |
| C. morifolium 1451103 | C. morifolium 140660 F3H | 0.702853 | | 0.477904 | F3H | MYB | purple |
| C. morifolium 1451103 | C. morifolium 14381001 F3H | 0.807766 | | 0.49198 | F3H | MYB | purple |
| C. morifolium 1451103 | C. morifolium 156055 CHS | 0.858618 | | 0.580172 | CHS | MYB | purple |
| C. morifolium 1451103 | C. morifolium 174438 CHS | 0.785802 | | 0.492009 | CHS | MYB | purple |
| C. morifolium 1451103 | C. morifolium 206 F3H | 0.686673 | | 0.502659 | F3H | MYB | purple |
| C. morifolium 1451103 | C. morifolium 36771 F3H | 0.681107 | | 0.510502 | F3H | MYB | purple |
| C. morifolium 1451103 | C. morifolium 38223 F3H | 0.721648 | | 0.494677 | F3H | MYB | purple |
| C. morifolium 1451103 | C. morifolium 46559 CHS | 0.643298 | | 0.535418 | CHS | MYB | purple |
| C. morifolium 1451103 | C. morifolium 559631 F3'H | 0.840499 | | 0.555064 | F3'H | MYB | purple |
| C. morifolium 1451103 | C. morifolium 9340138 CHS | 0.847207 | | 0.584977 | CHS | MYB | purple |
| C. morifolium 1451103 | C. morifolium 934078 DFR | 0.59908 | | 0.528027 | DFR | MYB | purple |
| C. morifolium 1451103 | C. morifolium asm16new307 F3'H | 0.840499 | | 0.555064 | F3'H | MYB | purple |
| C. morifolium 1451103 | C. morifolium 1081892 3GT | 0.602721 | | 0.546163 | 3GT | MYB | purple |
| C. morifolium 1451103 | C. morifolium 201250 3GT | 0.616268 | | 0.540149 | 3GT | MYB | purple |
| C. morifolium 1451103 | C. morifolium 37122 ANS2 | 0.494774 | | 0.51238 | ANS2 | MYB | purple |
| C. morifolium 878863 | C. morifolium 225621 PSY | 0.709547 | | 0.831582 | PSY | ERF | yellow |
| C. morifolium 692109 | C. morifolium 225621 PSY | 0.451288 | | 0.548607 | PSY | ERF | yellow |
| C. morifolium asm7new1728 | C. morifolium 121984 PORA | 0.57056 | | 0.650126 | PORA | M-type_MADS | green |
| C. morifolium asm7new1726 | C. morifolium 121984 PORA | 0.792889 | | 0.491636 | PORA | M-type_MADS | green |
| C. morifolium 30301 | C. morifolium 500315 CCD | 0.42669 | | 0.855989 | CCD | VOZ | yellow |
| C. morifolium 147018 | C. morifolium asm13new2404 3GT | 0.731013 | | 0.585729 | 3GT | GRAS | purple |
| C. morifolium 1697102 | C. morifolium 500315 CCD | 0.485973 | | 0.61247 | CCD | WRKY | yellow |
| C. morifolium 361556 | C. morifolium 121984 PORA | 0.870524 | | 0.67717 | PORA | WRKY | green |
| C. morifolium 361556 | C. morifolium 1062827 PORC | 0.494561 | | 0.544249 | PORC | WRKY | green |
| C. morifolium 361556 | C. morifolium 500315 CCD | 0.623531 | | 0.819874 | CCD | WRKY | yellow |
| C. morifolium 3326180 | C. morifolium 121984 PORA | 0.836877 | | 0.499776 | PORA | ERF | green |
| C. morifolium 3326180 | C. morifolium 1062827 PORC | 0.456908 | | 0.791724 | PORC | ERF | green |
| C. morifolium 3326180 | C. morifolium 500315 CCD | 0.526481 | | 0.818354 | CCD | ERF | yellow |
| C. morifolium 181219 | C. morifolium 121984 PORA | 0.726924 | | 0.480761 | PORA | WRKY | green |
| C. morifolium 113298 | C. morifolium 121984 PORA | 0.488904 | | 0.770628 | PORA | C2H2 | green |
| C. morifolium 113298 | C. morifolium 500315 CCD | 0.413052 | | 0.730638 | CCD | C2H2 | yellow |
| C. morifolium 58291 | C. morifolium 121984 PORA | 0.958704 | | 0.588601 | PORA | MIKC_MADS | green |
| C. morifolium 58291 | C. morifolium 804022 PDS | 0.940906 | | 0.50858 | PDS | MIKC_MADS | yellow |
| C. morifolium 58291 | C. morifolium 539433 ZDS | 0.917746 | | 0.412624 | ZDS | MIKC_MADS | yellow |
| C. morifolium 58291 | C. morifolium 500315 CCD | 0.756536 | | 0.87455 | CCD | MIKC_MADS | yellow |
| C. morifolium 6893234 | C. morifolium 121984 PORA | 0.559813 | | 0.423456 | PORA | C2H2 | green |
| C. morifolium 75986 | C. morifolium 121984 PORA | 0.479694 | | 0.662633 | PORA | TCP | green |
| C. morifolium 75986 | C. morifolium 1062827 PORC | 0.515669 | | 0.6535 | PORC | TCP | green |
| C. morifolium 182257 | C. morifolium 121984 PORA | 0.544437 | | 0.495922 | PORA | WRKY | green |
| C. morifolium 182257 | C. morifolium 1062827 PORC | 0.493232 | | 0.691228 | PORC | WRKY | green |
| C. morifolium 182257 | C. morifolium 804022 PDS | 0.570276 | | 0.468925 | PDS | WRKY | yellow |
| C. morifolium 28695 | C. morifolium 121984 PORA | 0.678387 | | 0.902846 | PORA | ERF | green |
| C. morifolium 28695 | C. morifolium 225621 PSY | 0.736184 | | 0.533344 | PSY | ERF | yellow |
| C. morifolium 1099236 | C. morifolium 121984 PORA | 0.738467 | | 0.99706 | PORA | MYB | green |
| C. morifolium 1099236 | C. morifolium 225621 PSY | 0.712741 | | 0.471894 | PSY | MYB | yellow |
| C. morifolium 824957 | C. morifolium 113088 F3'H | 0.744454 | | 0.41494 | F3'H | ERF | purple |
| C. morifolium 824957 | C. morifolium 1139541 CHI | 0.678868 | | 0.431474 | CHI | ERF | purple |
| C. morifolium 824957 | C. morifolium 114633 CHS | 0.615822 | | 0.404214 | CHS | ERF | purple |
| C. morifolium 824957 | C. morifolium 156055 CHS | 0.70423 | | 0.411939 | CHS | ERF | purple |
| C. morifolium 824957 | C. morifolium 46559 CHS | 0.988631 | | 0.41236 | CHS | ERF | purple |
| C. morifolium 824957 | C. morifolium 559631 F3'H | 0.539347 | | 0.414743 | F3'H | ERF | purple |
| C. morifolium 824957 | C. morifolium asm16new307 F3'H | 0.539347 | | 0.414743 | F3'H | ERF | purple |
| C. morifolium 361788 | C. morifolium 121984 PORA | 0.843864 | | 0.799586 | PORA | ERF | green |
| C. morifolium 361788 | C. morifolium 804022 PDS | 0.79574 | | 0.418336 | PDS | ERF | yellow |
| C. morifolium 361788 | C. morifolium 539433 ZDS | 0.882702 | | 0.471478 | ZDS | ERF | yellow |
| C. morifolium 361788 | C. morifolium 500315 CCD | 0.547153 | | 0.632193 | CCD | ERF | yellow |
| C. morifolium 108296 | C. morifolium 121984 PORA | 0.934591 | | 0.717868 | PORA | ERF | green |
| C. morifolium 108296 | C. morifolium 500315 CCD | 0.635854 | | 0.782638 | CCD | ERF | yellow |
| C. morifolium 174265 | C. morifolium 121984 PORA | 0.697939 | | 0.839735 | PORA | MYB | green |
| C. morifolium 7419236 | C. morifolium 121984 PORA | 0.624295 | | 0.501097 | PORA | ERF | green |
| C. morifolium 106596 | C. morifolium 121984 PORA | 0.729598 | | 0.418091 | PORA | WRKY | green |
| C. morifolium 422311 | C. morifolium 121984 PORA | 0.667571 | | 0.897261 | PORA | bHLH | green |
| C. morifolium 422311 | C. morifolium 225621 PSY | 0.667604 | | 0.73028 | PSY | bHLH | yellow |
| C. morifolium 78237 | C. morifolium 121984 PORA | 0.673654 | | 0.774017 | PORA | MYB_related | green |
| C. morifolium 78237 | C. morifolium 500315 CCD | 0.462118 | | 0.720328 | CCD | MYB_related | yellow |
| C. morifolium 1749435 | C. morifolium 121984 PORA | 0.724499 | | 0.715785 | PORA | C2H2 | green |
| C. morifolium 1749435 | C. morifolium 500315 CCD | 0.565253 | | 0.69748 | CCD | C2H2 | yellow |
| C. morifolium 1749666 | C. morifolium 121984 PORA | 0.571776 | | 0.810092 | PORA | C2H2 | green |
| C. morifolium 770260 | C. morifolium 121984 PORA | 0.822718 | | 0.809838 | PORA | C2H2 | green |
| C. morifolium 770260 | C. morifolium 500315 CCD | 0.616718 | | 0.579396 | CCD | C2H2 | yellow |
| C. morifolium 764299 | C. morifolium 1719125 GSA2(HEML2) | 0.484581 | | 0.668231 | GSA2  (HEML2) | MYB | green |
| C. morifolium 764299 | C. morifolium 1659259 HEME2 | 0.45367 | | 0.87487 | HEME2 | MYB | green |
| C. morifolium 1170588 | C. morifolium 121984 PORA | 0.610447 | | 0.774987 | PORA | ERF | green |
| C. morifolium 1170588 | C. morifolium 225621 PSY | 0.656092 | | 0.569445 | PSY | ERF | yellow |
| C. morifolium 56646 | C. morifolium 121984 PORA | 0.619655 | | 0.813906 | PORA | ERF | green |
| C. morifolium 30186 | C. morifolium 121984 PORA | 0.662501 | | 0.764349 | PORA | WRKY | green |
| C. morifolium 1474198 | C. morifolium 121984 PORA | 0.60078 | | 0.43785 | PORA | HD-ZIP | green |
| C. morifolium 10264300 | C. morifolium asm13new2404 3GT | 0.872827 | | 0.662764 | 3GT | ERF | purple |
| C. morifolium 691689 | C. morifolium 121984 PORA | 0.52775 | | 0.498798 | PORA | ERF | green |
| C. morifolium 4568142 | C. morifolium 121984 PORA | 0.697592 | | 0.573201 | PORA | HD-ZIP | green |
| C. morifolium 598418 | C. morifolium 121984 PORA | 0.64801 | | 0.935794 | PORA | ERF | green |
| C. morifolium 808583 | C. morifolium 121984 PORA | 0.625036 | | 0.550303 | PORA | C3H | green |
| C. morifolium 1333588 | C. morifolium 121984 PORA | 0.716629 | | 0.529553 | PORA | ERF | green |
| C. morifolium 110403 | C. morifolium 121984 PORA | 0.6339 | | 0.425512 | PORA | WRKY | green |
| C. morifolium 149253 | C. morifolium 121984 PORA | 0.420747 | | 0.496087 | PORA | WRKY | green |
| C. morifolium 581227 | C. morifolium 121984 PORA | 0.58797 | | 0.632363 | PORA | C3H | green |
| C. morifolium 170964 | C. morifolium 121984 PORA | 0.750804 | | 0.441866 | PORA | MYB | green |
| C. morifolium 170964 | C. morifolium 1062827 PORC | 0.531053 | | 0.82535 | PORC | MYB | green |
| C. morifolium 170964 | C. morifolium 500315 CCD | 0.415643 | | 0.762171 | CCD | MYB | yellow |
| C. morifolium 351636 | C. morifolium 140660 F3H | 0.450988 | | 0.428327 | F3H | C2H2 | purple |
| C. morifolium 351636 | C. morifolium 174438 CHS | 0.482526 | | 0.465709 | CHS | C2H2 | purple |
| C. morifolium 351636 | C. morifolium 206 F3H | 0.487572 | | 0.498461 | F3H | C2H2 | purple |
| C. morifolium 351636 | C. morifolium 36771 F3H | 0.467343 | | 0.518535 | F3H | C2H2 | purple |
| C. morifolium 351636 | C. morifolium 38223 F3H | 0.579152 | | 0.455946 | F3H | C2H2 | purple |
| C. morifolium 351636 | C. morifolium 46559 CHS | 0.542577 | | 0.559334 | CHS | C2H2 | purple |
| C. morifolium 351636 | C. morifolium 934078 DFR | 0.516221 | | 0.505009 | DFR | C2H2 | purple |
| C. morifolium 351636 | C. morifolium 1081892 3GT | 0.567103 | | 0.524076 | 3GT | C2H2 | purple |
| C. morifolium 351636 | C. morifolium 201250 3GT | 0.547457 | | 0.527659 | 3GT | C2H2 | purple |
| C. morifolium 351636 | C. morifolium asm13new2404 3GT | 0.745222 | | 0.524076 | 3GT | C2H2 | purple |
| C. morifolium 351636 | C. morifolium 37122 ANS2 | 0.469259 | | 0.483702 | ANS2 | C2H2 | purple |
| C. morifolium 17652 | C. morifolium asm13new2404 3GT | 0.961613 | | 0.614717 | 3GT | ERF | purple |
| C. morifolium 669838 | C. morifolium asm13new2404 3GT | 0.961613 | | 0.614717 | 3GT | ERF | purple |
| C. morifolium 168245 | C. morifolium 121984 PORA | 0.665488 | | 0.473085 | PORA | GRAS | green |
| C. morifolium 1248116 | C. morifolium 121984 PORA | 0.584097 | | 0.917044 | PORA | ERF | green |
| C. morifolium 249115 | C. morifolium 121984 PORA | 0.819824 | | 0.457352 | PORA | ERF | green |
| C. morifolium 249115 | C. morifolium 1062827 PORC | 0.471817 | | 0.820435 | PORC | ERF | green |
| C. morifolium 249115 | C. morifolium 500315 CCD | 0.5011 | | 0.830309 | CCD | ERF | yellow |
| C. morifolium 1146479 | C. morifolium 121984 PORA | 0.72708 | | 0.505496 | PORA | MYB_related | green |
| C. morifolium 911187 | C. morifolium 121984 PORA | 0.892217 | | 0.619773 | PORA | ERF | green |
| C. morifolium 911187 | C. morifolium 1062827 PORC | 0.419355 | | 0.621041 | PORC | ERF | green |
| C. morifolium 911187 | C. morifolium 500315 CCD | 0.642314 | | 0.856678 | CCD | ERF | yellow |
| C. morifolium 669837 | C. morifolium asm13new2404 3GT | 0.935018 | | 0.45922 | 3GT | ERF | purple |
| C. morifolium 285338 | C. morifolium 121984 PORA | 0.835375 | | 0.985956 | PORA | Nin-like | green |
| C. morifolium 3601235 | C. morifolium 121984 PORA | 0.744984 | | 0.563797 | PORA | MYB | green |
| C. morifolium 3601235 | C. morifolium 1062827 PORC | 0.508796 | | 0.733426 | PORC | MYB | green |
| C. morifolium 798334 | C. morifolium 121984 PORA | 0.710417 | | 0.512596 | PORA | HD-ZIP | green |
| C. morifolium 994639 | C. morifolium 121984 PORA | 0.836035 | | 0.862829 | PORA | NAC | green |
| C. morifolium 994639 | C. morifolium 500315 CCD | 0.528506 | | 0.436616 | CCD | NAC | yellow |
| C. morifolium 105810 | C. morifolium 121984 PORA | 0.405958 | | 0.528307 | PORA | ERF | green |
| C. morifolium 1024382 | C. morifolium 121984 PORA | 0.908744 | | 0.656857 | PORA | ERF | green |
| C. morifolium 1024382 | C. morifolium 500315 CCD | 0.6269 | | 0.829501 | CCD | ERF | yellow |
| C. morifolium 117390 | C. morifolium 121984 PORA | 0.977969 | | 0.58963 | PORA | MYB | green |
| C. morifolium 117390 | C. morifolium 804022 PDS | 0.850304 | | 0.687679 | PDS | MYB | yellow |
| C. morifolium 117390 | C. morifolium 539433 ZDS | 0.831089 | | 0.740893 | ZDS | MYB | yellow |
| C. morifolium 117390 | C. morifolium 3636191 CRTISO | 0.900642 | | 0.408152 | CRTISO | MYB | yellow |
| C. morifolium 117390 | C. morifolium 500315 CCD | 0.724484 | | 0.665593 | CCD | MYB | yellow |
| C. morifolium 5424107 | C. morifolium 121984 PORA | 0.643333 | | 0.71519 | PORA | GRAS | green |
| C. morifolium 4190216 | C. morifolium 121984 PORA | 0.748857 | | 0.433141 | PORA | ERF | green |
| C. morifolium 4190216 | C. morifolium 804022 PDS | 0.477699 | | 0.713625 | PDS | ERF | yellow |
| C. morifolium 4190216 | C. morifolium 539433 ZDS | 0.581688 | | 0.618787 | ZDS | ERF | yellow |
| C. morifolium 4190216 | C. morifolium 500315 CCD | 0.42214 | | 0.900405 | CCD | ERF | yellow |
| C. morifolium 1146437 | C. morifolium 804022 PDS | 0.457942 | | 0.424136 | PDS | LBD | yellow |
| C. morifolium 1146437 | C. morifolium 500315 CCD | 0.57186 | | 0.951955 | CCD | LBD | yellow |
| C. morifolium 1353 | C. morifolium 36771 F3H | 0.654828 | | 0.404512 | F3H | ARR-B | purple |
| C. morifolium 1353 | C. morifolium 46559 CHS | 0.571065 | | 0.441652 | CHS | ARR-B | purple |
| C. morifolium 1353 | C. morifolium 934078 DFR | 0.689917 | | 0.410319 | DFR | ARR-B | purple |
| C. morifolium 1353 | C. morifolium 1081892 3GT | 0.74208 | | 0.432126 | 3GT | ARR-B | purple |
| C. morifolium 1353 | C. morifolium 201250 3GT | 0.681441 | | 0.429584 | 3GT | ARR-B | purple |
| C. morifolium 1353 | C. morifolium 1266437 3MT | 0.852008 | | 0.420036 | 3MT | ARR-B | purple |
| C. morifolium 1353 | C. morifolium 144657 3MT | 0.85864 | | 0.427018 | 3MT | ARR-B | purple |
| C. morifolium 1622283 | C. morifolium 500315 CCD | 0.491146 | | 0.85727 | CCD | NAC | yellow |
| C. morifolium 1806852 | C. morifolium 121984 PORA | 0.517878 | | 0.733299 | PORA | DBB | green |
| C. morifolium 1806852 | C. morifolium 500315 CCD | 0.498554 | | 0.766722 | CCD | DBB | yellow |
| C. morifolium 1260337 | C. morifolium 1081892 3GT | 0.412753 | | 0.591759 | 3GT | bZIP | purple |
| C. morifolium 1260337 | C. morifolium 10628156 3MT | 0.69508 | | 0.559924 | 3MT | bZIP | purple |
| C. morifolium 1260337 | C. morifolium 1266437 3MT | 0.697515 | | 0.520255 | 3MT | bZIP | purple |
| C. morifolium 1260337 | C. morifolium 144657 3MT | 0.685366 | | 0.513495 | 3MT | bZIP | purple |
| C. morifolium 1260337 | C. morifolium 37122 ANS2 | 0.573713 | | 0.55477 | ANS2 | bZIP | purple |
| C. morifolium 570336 | C. morifolium 121984 PORA | 0.588314 | | 0.592911 | PORA | ERF | green |
| C. morifolium 1659446 | C. morifolium asm13new2404 3GT | 0.940734 | | 0.581667 | 3GT | C3H | purple |
| C. morifolium 10264116 | C. morifolium 121984 PORA | 0.840947 | | 0.910346 | PORA | C2H2 | green |
| C. morifolium 10264116 | C. morifolium 500315 CCD | 0.621908 | | 0.484397 | CCD | C2H2 | yellow |
| C. morifolium 581199 | C. morifolium 121984 PORA | 0.59266 | | 0.728687 | PORA | C3H | green |
| C. morifolium 350101 | C. morifolium 804022 PDS | 0.794975 | | 0.792208 | PDS | DBB | yellow |
| C. morifolium 350101 | C. morifolium 539433 ZDS | 0.725073 | | 0.688542 | ZDS | DBB | yellow |
| C. morifolium 350101 | C. morifolium 500315 CCD | 0.583297 | | 0.905062 | CCD | DBB | yellow |
| C. morifolium 1660118 | C. morifolium 121984 PORA | 0.972185 | | 0.798906 | PORA | ARF | green |
| C. morifolium 1660118 | C. morifolium 804022 PDS | 0.900804 | | 0.437314 | PDS | ARF | yellow |
| C. morifolium 1660118 | C. morifolium 539433 ZDS | 0.924233 | | 0.591944 | ZDS | ARF | yellow |
| C. morifolium 1660118 | C. morifolium 500315 CCD | 0.780672 | | 0.426973 | CCD | ARF | yellow |
| C. morifolium 40957 | C. morifolium 121984 PORA | 0.720356 | | 0.413695 | PORA | LBD | green |
| C. morifolium 40957 | C. morifolium 804022 PDS | 0.624548 | | 0.553768 | PDS | LBD | yellow |
| C. morifolium 40957 | C. morifolium 500315 CCD | 0.404772 | | 0.957689 | CCD | LBD | yellow |
| C. morifolium 141018 | C. morifolium 804022 PDS | 0.462873 | | 0.54405 | PDS | ERF | yellow |
| C. morifolium 141018 | C. morifolium 539433 ZDS | 0.761614 | | 0.410756 | ZDS | ERF | yellow |
| C. morifolium 33951 | C. morifolium 121984 PORA | 0.933185 | | 0.59809 | PORA | ERF | green |
| C. morifolium 33951 | C. morifolium 500315 CCD | 0.719597 | | 0.870561 | CCD | ERF | yellow |
| C. morifolium 1024380 | C. morifolium 121984 PORA | 0.74778 | | 0.927209 | PORA | ERF | green |
| C. morifolium 1024380 | C. morifolium 500315 CCD | 0.505201 | | 0.484112 | CCD | ERF | yellow |
| C. morifolium 1026979 | C. morifolium asm13new2404 3GT | 0.947556 | | 0.696541 | 3GT | C3H | purple |
| C. morifolium 925850 | C. morifolium 113088 F3'H | 0.43382 | | 0.519147 | F3'H | C2H2 | purple |
| C. morifolium 925850 | C. morifolium 14381001 F3H | 0.671591 | | 0.426315 | F3H | C2H2 | purple |
| C. morifolium 925850 | C. morifolium 156055 CHS | 0.404179 | | 0.508368 | CHS | C2H2 | purple |
| C. morifolium 925850 | C. morifolium 174438 CHS | 0.745254 | | 0.425852 | CHS | C2H2 | purple |
| C. morifolium 925850 | C. morifolium 206 F3H | 0.746559 | | 0.459202 | F3H | C2H2 | purple |
| C. morifolium 925850 | C. morifolium 36771 F3H | 0.729403 | | 0.479707 | F3H | C2H2 | purple |
| C. morifolium 925850 | C. morifolium 38223 F3H | 0.811006 | | 0.416031 | F3H | C2H2 | purple |
| C. morifolium 925850 | C. morifolium 46559 CHS | 0.791548 | | 0.521618 | CHS | C2H2 | purple |
| C. morifolium 925850 | C. morifolium 9340138 CHS | 0.460848 | | 0.486586 | CHS | C2H2 | purple |
| C. morifolium 925850 | C. morifolium 934078 DFR | 0.765709 | | 0.466276 | DFR | C2H2 | purple |
| C. morifolium 925850 | C. morifolium 1081892 3GT | 0.798041 | | 0.485948 | 3GT | C2H2 | purple |
| C. morifolium 925850 | C. morifolium 201250 3GT | 0.790734 | | 0.489471 | 3GT | C2H2 | purple |
| C. morifolium 925850 | C. morifolium asm13new2404 3GT | 0.520083 | | 0.485948 | 3GT | C2H2 | purple |
| C. morifolium 925850 | C. morifolium 10628156 3MT | 0.510093 | | 0.449845 | 3MT | C2H2 | purple |
| C. morifolium 925850 | C. morifolium 1266437 3MT | 0.47796 | | 0.420052 | 3MT | C2H2 | purple |
| C. morifolium 925850 | C. morifolium 144657 3MT | 0.516667 | | 0.415697 | 3MT | C2H2 | purple |
| C. morifolium 925850 | C. morifolium 37122 ANS2 | 0.693708 | | 0.444414 | ANS2 | C2H2 | purple |
| C. morifolium 1034690 | C. morifolium 1062827 PORC | 0.481675 | | 0.75238 | PORC | GRAS | green |
| C. morifolium 1034690 | C. morifolium 804022 PDS | 0.828904 | | 0.672235 | PDS | GRAS | yellow |
| C. morifolium 1034690 | C. morifolium 539433 ZDS | 0.784024 | | 0.463193 | ZDS | GRAS | yellow |
| C. morifolium 1034690 | C. morifolium 500315 CCD | 0.523601 | | 0.992584 | CCD | GRAS | yellow |
| C. morifolium 149246 | C. morifolium 121984 PORA | 0.864407 | | 0.513724 | PORA | LSD | green |
| C. morifolium 149246 | C. morifolium 804022 PDS | 0.749735 | | 0.511096 | PDS | LSD | yellow |
| C. morifolium 149246 | C. morifolium 500315 CCD | 0.73863 | | 0.918778 | CCD | LSD | yellow |
| C. morifolium 615892 | C. morifolium 121984 PORA | 0.842231 | | 0.542597 | PORA | ERF | green |
| C. morifolium 615892 | C. morifolium 1062827 PORC | 0.455915 | | 0.754215 | PORC | ERF | green |
| C. morifolium 615892 | C. morifolium 500315 CCD | 0.530866 | | 0.832656 | CCD | ERF | yellow |
| C. morifolium 153724 | C. morifolium 121984 PORA | 0.819778 | | 0.587021 | PORA | GATA | green |
| C. morifolium 153724 | C. morifolium 804022 PDS | 0.774936 | | 0.538118 | PDS | GATA | yellow |
| C. morifolium 153724 | C. morifolium 539433 ZDS | 0.910707 | | 0.452888 | ZDS | GATA | yellow |
| C. morifolium 153724 | C. morifolium 500315 CCD | 0.56226 | | 0.86858 | CCD | GATA | yellow |
| C. morifolium 477584 | C. morifolium 121984 PORA | 0.673274 | | 0.479605 | PORA | MYB | green |
| C. morifolium 477584 | C. morifolium 804022 PDS | 0.438327 | | 0.654669 | PDS | MYB | yellow |
| C. morifolium 477584 | C. morifolium 539433 ZDS | 0.47985 | | 0.555356 | ZDS | MYB | yellow |
| C. morifolium 1749144 | C. morifolium 121984 PORA | 0.928202 | | 0.999143 | PORA | WRKY | green |
| C. morifolium 1749144 | C. morifolium 225621 PSY | 0.715263 | | 0.453194 | PSY | WRKY | yellow |
| C. morifolium 330911 | C. morifolium asm13new2404 3GT | 0.862448 | | 0.543757 | 3GT | ERF | purple |
| C. morifolium 25470 | C. morifolium 121984 PORA | 0.464822 | | 0.841876 | PORA | ERF | green |
| C. morifolium 25470 | C. morifolium 225621 PSY | 0.676315 | | 0.408814 | PSY | ERF | yellow |
| C. morifolium 670250 | C. morifolium 500315 CCD | 0.863621 | | 0.464226 | CCD | TALE | yellow |
| C. morifolium 25186 | C. morifolium 121984 PORA | 0.852544 | | 0.760974 | PORA | bHLH | green |
| C. morifolium 25186 | C. morifolium 500315 CCD | 0.710136 | | 0.728997 | CCD | bHLH | yellow |
| C. morifolium 95081 | C. morifolium 839138 GSA1(HEML1) | 0.824979 | | 0.932836 | GSA1  (HEML1) | DBB | green |
| C. morifolium 95081 | C. morifolium 1719125 GSA2(HEML2) | 0.961805 | | 0.656175 | GSA2  (HEML2) | DBB | green |
| C. morifolium 95081 | C. morifolium 66299 HEME1 | 0.807943 | | 0.798092 | HEME1 | DBB | green |
| C. morifolium 95081 | C. morifolium 2224124 HEMF1 | 0.872088 | | 0.573567 | HEMF1 | DBB | green |
| C. morifolium 95081 | C. morifolium 53571 CHL12 | 0.432991 | | 0.818173 | CHL12 | DBB | green |
| C. morifolium 95081 | C. morifolium 6552 CHLD | 0.457481 | | 0.728833 | CHLD | DBB | green |
| C. morifolium 95081 | C. morifolium 466135 CHLG | 0.863174 | | 0.524513 | CHLG | DBB | green |
| C. morifolium 95081 | C. morifolium 1058101 LCY-B | 0.627362 | | 0.770696 | LCY-B | DBB | yellow |
| C. morifolium 95081 | C. morifolium 113088 F3'H | 0.887324 | | 0.825789 | F3'H | DBB | purple |
| C. morifolium 95081 | C. morifolium 1139541 CHI | 0.90662 | | 0.814234 | CHI | DBB | purple |
| C. morifolium 95081 | C. morifolium 114633 CHS | 0.954299 | | 0.851964 | CHS | DBB | purple |
| C. morifolium 95081 | C. morifolium 140660 F3H | 0.545317 | | 0.887502 | F3H | DBB | purple |
| C. morifolium 95081 | C. morifolium 14381001 F3H | 0.6776 | | 0.867334 | F3H | DBB | purple |
| C. morifolium 95081 | C. morifolium 156055 CHS | 0.905772 | | 0.846158 | CHS | DBB | purple |
| C. morifolium 95081 | C. morifolium 174438 CHS | 0.61052 | | 0.867689 | CHS | DBB | purple |
| C. morifolium 95081 | C. morifolium 206 F3H | 0.510335 | | 0.847847 | F3H | DBB | purple |
| C. morifolium 95081 | C. morifolium 36771 F3H | 0.51631 | | 0.835574 | F3H | DBB | purple |
| C. morifolium 95081 | C. morifolium 38223 F3H | 0.477202 | | 0.876101 | F3H | DBB | purple |
| C. morifolium 95081 | C. morifolium 46559 CHS | 0.447666 | | 0.813589 | CHS | DBB | purple |
| C. morifolium 95081 | C. morifolium 559631 F3'H | 0.97197 | | 0.826162 | F3'H | DBB | purple |
| C. morifolium 95081 | C. morifolium 9340138 CHS | 0.869428 | | 0.864511 | CHS | DBB | purple |
| C. morifolium 95081 | C. morifolium 934078 DFR | 0.411629 | | 0.855191 | DFR | DBB | purple |
| C. morifolium 95081 | C. morifolium asm16new307 F3'H | 0.97197 | | 0.826162 | F3'H | DBB | purple |
| C. morifolium 95081 | C. morifolium 201250 3GT | 0.408613 | | 0.842925 | 3GT | DBB | purple |
| C. morifolium 279241 | C. morifolium 121984 PORA | 0.564046 | | 0.913078 | PORA | DBB | green |
| C. morifolium 279241 | C. morifolium 500315 CCD | 0.627643 | | 0.52471 | CCD | DBB | yellow |
| C. morifolium 2263 | C. morifolium 121984 PORA | 0.617908 | | 0.62673 | PORA | bHLH | green |
| C. morifolium 2263 | C. morifolium 1062827 PORC | 0.43889 | | 0.631056 | PORC | bHLH | green |
| C. morifolium 2263 | C. morifolium 500315 CCD | 0.936224 | | 0.847031 | CCD | bHLH | yellow |
| C. morifolium 9340601 | C. morifolium 121984 PORA | 0.891488 | | 0.845511 | PORA | MYB | green |
| C. morifolium 9340601 | C. morifolium 539433 ZDS | 0.717908 | | 0.49153 | ZDS | MYB | yellow |
| C. morifolium 9340601 | C. morifolium 500315 CCD | 0.503882 | | 0.495117 | CCD | MYB | yellow |
| C. morifolium 1156220 | C. morifolium 121984 PORA | 0.649335 | | 0.471233 | PORA | MYB | green |
| C. morifolium 1156220 | C. morifolium 539433 ZDS | 0.479871 | | 0.577712 | ZDS | MYB | yellow |

**Table S5.** Information on candidate upstream regulatory proteins identified by Y1H screening using the *CmMYB4* promoter as bait.

| **Homologous Protein** | **Species** | **Accession Number** | **Identity** |
| --- | --- | --- | --- |
| serine/arginine-rich splicing factor SR34A-like | *Tanacetum cinerariifolium* | GEY08494.1 | 94% |
| chlorophyll a-b binding protein | *Artemisia annua* | PWA97683.1 | 94% |
| adenosylhomocysteinase 1 | *Tanacetum cinerariifolium* | GEU50567.1 | 99% |
| VAN3-binding protein | *Cynara cardunculus*  var. *scolymus* | XP_024968591.1 | 88% |
| auxin response factor 4-like | *Tanacetum cinerariifolium* | GEZ89305.1 | 80% |
| ABC transporter E family member 2 | *Tanacetum cinerariifolium* | GEU79582.1 | 99% |
| GDSL esterase/lipase At5g45960-like | *Erigeron canadensis* | XP_043620824.1 | 82% |
| Glutamate-cysteine ligase protein | *Artemisia annua* | PWA60569.1 | 97% |
| Pectinesterase inhibitor | *Artemisia annua* | PWA45020.1 | 93% |
| transcription factor bHLH47 | *Helianthus annuus* | XP_021983323.1 | 72% |
| CBL-interacting serine/threonine-protein kinase 6-like | *Cynara cardunculus*  var. *scolymus* | XP_024961027.1 | 96% |
| transcription repressor MYB4 | *Artemisia annua* | PWA43034.1 | 97% |
| photosystem II 22 kDa protein, chloroplastic | *Helianthus annuus* | XP_022024879.1 | 86% |
| light-induced protein, chloroplastic-like | *Tanacetum cinerariifolium* | GEW27671.1 | 93% |
| SAM synthetase | *Artemisia annua* | AGH17770.1 | 98% |
| PB1 domain, Auxin response factor, DNA-binding pseudobarrel domain protein | *Artemisia annua* | PWA70953.1 | 89% |
| basic helix-loop-helix (bHLH) domain-containing protein | *Artemisia annua* | PWA77805.1 | 95% |
| FT-interacting protein 1 | *Tanacetum cinerariifolium* | GEX46028.1 | 99% |
| UBA-like protein | *Artemisia annua* | PWA57504.1 | 91% |
| ethylene-responsive transcription factor 3 | *Artemisia annua* | PWA85016.1 | 95% |
